# Evaluating metagenomic assembly approaches for biome-specific gene catalogues

**DOI:** 10.1186/s40168-022-01259-2

**Published:** 2022-05-06

**Authors:** Luis Fernando Delgado, Anders F. Andersson

**Affiliations:** Department of Gene Technology, Science for Life Laboratory, School of Engineering Sciences in Chemistry, Biotechnology and Health, KTH Royal Institute of Technology, Stockholm, Sweden

**Keywords:** Gene catalogue, Brackish water, Metagenomics, Assembly approach, Mix assembly, Baltic Sea

## Abstract

**Background:**

For many environments, biome-specific microbial gene catalogues are being recovered using shotgun metagenomics followed by assembly and gene calling on the assembled contigs. The assembly is typically conducted either by individually assembling each sample or by co-assembling reads from all the samples. The co-assembly approach can potentially recover genes that display too low abundance to be assembled from individual samples. On the other hand, combining samples increases the risk of mixing data from closely related strains, which can hamper the assembly process. In this respect, assembly on individual samples followed by clustering of (near) identical genes is preferable. Thus, both approaches have potential pros and cons, but it remains to be evaluated which assembly strategy is most effective. Here, we have evaluated three assembly strategies for generating gene catalogues from metagenomes using a dataset of 124 samples from the Baltic Sea: (1) assembly on individual samples followed by clustering of the resulting genes, (2) co-assembly on all samples, and (3) mix assembly, combining individual and co-assembly.

**Results:**

The mix-assembly approach resulted in a more extensive nonredundant gene set than the other approaches and with more genes predicted to be complete and that could be functionally annotated. The mix assembly consists of 67 million genes (Baltic Sea gene set, BAGS) that have been functionally and taxonomically annotated. The majority of the BAGS genes are dissimilar (< 95% amino acid identity) to the Tara Oceans gene dataset, and hence, BAGS represents a valuable resource for brackish water research.

**Conclusion:**

The mix-assembly approach represents a feasible approach to increase the information obtained from metagenomic samples.

**Video abstract**

**Supplementary Information:**

The online version contains supplementary material available at 10.1186/s40168-022-01259-2.

## Background

High-throughput sequencing has led to the establishment of the field of metagenomics, allowing the direct analysis of genetic material contained within an environmental sample [1]. This approach offers a detailed characterization of complex microbial communities without the need for cultivation. It can be used to address questions like *which* microorganisms are present, *what* are they capable of doing, and *how* do they interact. Metagenomics has been used for studying several ecosystem types, such as soils, human guts, and oceans [2–4].

For many environments, biome-specific gene catalogues have been recovered using shotgun metagenomics, followed by assembly and gene calling on the assembled contigs. Examples are the integrated reference catalogue of the human microbiome [4] and the Tara Oceans gene catalogue [2]. Gene catalogues facilitate the discovery of novel gene functions and gene variants. Annotated gene catalogues can also serve as genomic backbones onto which sequencing reads from metagenomes and metatranscriptomes, as well as mass-spectrometry spectra from metaproteomics, can be mapped, which enables fast and accurate taxonomic and functional profiling with such datasets.

The assembly can be carried out either by co-assembling reads from all the samples (or groups of samples) or individually assembling reads from each sample. The co-assembly approach has the advantage that some genes displaying too low abundance to be assembled from individual samples may reach enough coverage to be recovered. However, combining data from many samples often means mixing data from a diversity of closely related strains (from the same species). This fine-scale genomic variation can compromise the assembly process because the de Bruijn graph will include many alternative paths. Consequently, the assembler may decide to break the graph into smaller pieces, which can result in fragmentation of genes.

An alternative approach is to perform assembly on each sample individually. The individually assembled samples approach will minimize the mixing of data from different strains and therefore potentially result in more completely assembled genes, at least for fairly abundant genomes. However, another problem arises, which is that (more or less) identical genes from multiple samples will be reconstructed. To serve as a reference dataset, it is desirable to have a nonredundant set of genes. Sequence redundancy removal can be achieved by clustering the gene sequences (or their protein translations [5]) resulting from the different assemblies based on sequence similarity, using some cutoff criteria. For each gene cluster, a representative sequence is then chosen based on, e.g. gene completeness, centrality in the cluster, or abundance in the dataset.

Recently, a Baltic Sea specific gene catalogue with 6.8 million genes was constructed based on the metagenomic data from 81 water samples spanning the spatiotemporal gradients of the Baltic Sea [6]. For the construction of the Baltic Sea specific gene catalogue, all the 2.6 billion (i.e. 10^9^) reads were co-assembled, and genes called on all contigs > 1000 bp. While this gene catalogue has established itself as a useful resource for analysing metagenome and metatranscriptome datasets from brackish environments [7–11], only ca 10% of the shotgun reads from a typical Baltic Sea metagenome sample are mapping to genes with a functional annotation [6]. A reason for the seemingly low coverage could be that the co-assembly approach has resulted in a fragmented assembly. A more comprehensive reference gene catalogue would hence be desirable for this environment. In this study, we conduct an extensive comparison of three assembly approaches on an expanded set of metagenome samples from the Baltic Sea and present an updated gene catalogue for the Baltic Sea microbiome.

## Methods

### Metagenome samples

Five previously published sample sets [6, 7, 12] were used in this study. The sampling locations are shown in Additional file 1, and a brief description of sample retrieval and sequencing is given in Additional file 2; for further details, we refer to the original publications. Sequencing of all sample sets was conducted using Illumina HiSeq 2500.

### Preprocessing of reads

Removal of low-quality bases was performed earlier [7] using Cutadapt [13] (parameters-q 15, 15) followed by adapter removal (parameters-n 3 — minimum length 31). The resulting read files were thereafter screened for PCR duplicates using FastUniq [14] with default parameters.

### Assembly

Individual assemblies on the 124 samples were performed earlier [7], using MEGAHIT [15] v.1.1.2 with the “--presets meta-sensitive” option. For the co-assembly conducted here, all preprocessed reads were first combined and normalized using BBnorm of BBmap v.38.08 (https://sourceforge.net/projects/bbmap/) with the following parameters: target = 70, mindepth = 2, and prefilter = t. Also, the normalized read set was too extensive to allow co-assembly with the tag “presets –meta-sensitive” with MEGAHIT. Therefore, they were assembled with “--presets meta-large” (using MEGAHIT v.1.1.2), as recommended for complex metagenomes in the MEGAHIT documentation.

### Gene prediction

Genes were predicted on contigs (from the co-assembly and from the individual assemblies) using Prodigal [16] v.2.6.3 with the -p meta option. Gene completeness is based on Prodigal gene prediction. Complete genes refer to predicted genes having a predicted start and a stop codon (Prodigal indicator “00”); partial genes are predicted genes with either no start or stop codon (Prodigal indicator “01” or “10”), typically due to that the gene runs off the edge of a contig; and incomplete genes are predicted genes without a start and a stop codon (Prodigal indicator “11”).

### Protein clustering

Clustering of the proteins stemming from the different samples for the individual assembly, and from the co-assembly for the mix-assembly strategy, was performed using MMseqs2 [17] v9.d36de using the cascaded clustering mode (MMseqs2 cluster, https://mmseqs.com/latest/userguide.pdf). Clustering was first performed on the proteins from the individual assemblies, and the cluster-representative proteins were subsequently clustered with the co-assembly proteins. The following parameters were used in the two MMseqs2 runs: -c 0.95, --min-seq-id 0.95, --cov-mod 1, and --clust-mod 2. This means proteins displaying ≥ 95% amino acid identity were clustered. Strains belonging to the same prokaryotic species generally display > 95% average amino acid identity [18]. As recommended in the MMseq2 user guide, -cov-mod 1 was used, since it allows clustering of fragmented proteins (as often occurs in metagenomic datasets). With --cov-mode 1 only, sequences are clustered that have a sequence length overlap greater than the percentage specified by -c (i.e. 95% with -c 0.95) of the target sequence. In MMseqs2, the query is seen as the representative sequence, and the target is a member sequence. To lower the risk for fragmented proteins becoming cluster-representative sequences, -cluster-mode 2 was used, again following the recommendations of the MMseq2 user guide. It sorts sequences by length and in each clustering step forms a cluster containing the longest sequence and the sequences that it matches.

### Read mapping and counting

To reduce the computational burden of the read mapping, random subsets of 10,000 non-normalized forward reads per sample were created using seqtk v.1.2-r101-dirty (https://github.com/lh3/seqtk), with seed 100 (-s 100). These reads (12.4 million in total) were mapped to the representative gene sequences from the individual, co-, and mix assembly, respectively, using Bowtie2 v.2.3.4.3 [19], with the parameter “--local.” The resulting SAM files were converted to BAM with SAMtools v.1.9 [20]. The htseq-count script from HTSeq [21] v.0.11.2 was used to obtain raw counts per gene, with the parameters “-f bam -r pos -t CDS -i ID -s no -a 0”. For the counting, GFF input files were used, created using the script create_gff.py available at https://github.com/EnvGen/toolbox/tree/master/scripts. In order to estimate read depth coverage of the genes in the total metagenome, we multiplied the counts per gene by the average read-pair length divided by the length of the gene and multiplied this number with the total number of read pairs in the whole dataset divided by the total number of randomly sampled forward reads. This is a rough estimation of the coverage of each gene in the total metagenome; however, after normalisation with BBnorm, high coverage genes will get a lower coverage.

### Functional annotations

Functional annotation of proteins was conducted using EggNOG [22], Pfam [23], and dbCAN [24]. Annotations against Pfam v.31.0 and dbCAN v.5.0 were conducted with hmmsearch and hmmscan [25], respectively, in HMMER v.3.2.1, selecting hits with *E*-value < 0.001. Annotations against EggNOG v.4.5.1 were performed using eggNOG-mapper v.1.0.3 [26], using accelerated profile HMM searches [27], following the recommendation for setting up large annotation jobs.

### Taxonomic affiliation

MMseqs2 (v13.45111) taxonomy [28], with parameters “--orf-filter 0 --tax-lineage 1”, was used to assign taxonomic labels to contigs from which representative genes were predicted. MMseqs2 taxonomy uses an approximate 2bLCA (lowest common ancestor, LCA) approach. GTDB [29, 30] v.202 was used as a reference database for bacteria and archaea and Uniprot90 [31] (downloaded on June 4, 2021) for eukaryotes and viruses. An interactive chart for the gene set’s taxonomic information was generated using Krona (Ondov et al. 2011) (see Additional file 3).

### RNA gene screening

Barrnap v.0.9 [32], using default parameters, was used to identify potential rRNA genes, and identification of rRNA and other potential RNA genes in the mix-assembly gene set was conducted using the Rfam v.14.6 [33] database, with hmmsearch [25], in HMMER v.3.3.2, with flag “--cut_ga”. The union of genes identified as rRNA by Barnap and Rfam/hmmsearch was removed from the final gene set.

## Results

We used a set of 124 metagenome samples from the Baltic Sea ([6, 7, 12]; see Additional file 1) to evaluate three assembly approaches for generating a nonredundant gene catalogue: co-assembly on all samples (“co-assembly”), assembly on individual samples (“individual assembly”), and a combination of the previous two (“mix assembly”). For the co-assembly, due to the complexity of the dataset, direct co-assembly of all reads was not possible, even on a server with 1 TB of memory. Therefore, the reads were first normalized such that reads stemming from highly abundant genomes (with high-frequency *k*-mers) were downsampled (to a depth of 70× coverage), and those presumably derived from errors (with a depth below 2×) were removed. This reduced the total number of read pairs from 5.4 to 2.9 billion.

Since the contigs of the co-assembly are derived from reads from all samples, it will result in a nonredundant set of genes. In contrast, genes from the individually assembled samples may overlap between samples. To reduce this redundancy, clustering was conducted on the encoded proteins [17]. We used a cutoff of 95% amino acid identity, conforming to that strains belonging to the same species typically display more than 95% average amino acid identity [18]. This reduced the number of individual-assembly genes from 134 to 50 million. Likewise, clustering was conducted on the co-assembly proteins together with the nonredundant set of individual-assembly proteins, to generate the mix-assembly gene set.

The mix-assembly approach resulted in the largest number of nonredundant genes (67 M), followed by individual assembly (50 M) and co-assembly (45 M; Table 1). Mix assembly also had the largest number of genes predicted to be complete (12 M) followed closely by co-assembly (11 M) but twice as many as individual assembly (6 M; Table 1).

**Table 1:** Summary statistics for the different assembly approaches

Assembly approach	Total bps	Number of genes	Num. of genes ≥ 100 bp	Num. of complete genes	Num. of partial genes	No. of incompletegenes
*Individual*	18,770,879,205	50,045,582	45,859,319	6,258,868	27,073,554	16,713,160
*Co*	20,347,887,912	45,455,222	42,278,556	11,443,584	23,815,733	10,195,905
*Mix*	27,043,772,505	67,583,055	61,576,531	12,690,647	37,345,617	17,546,791

**Fig. 1:**
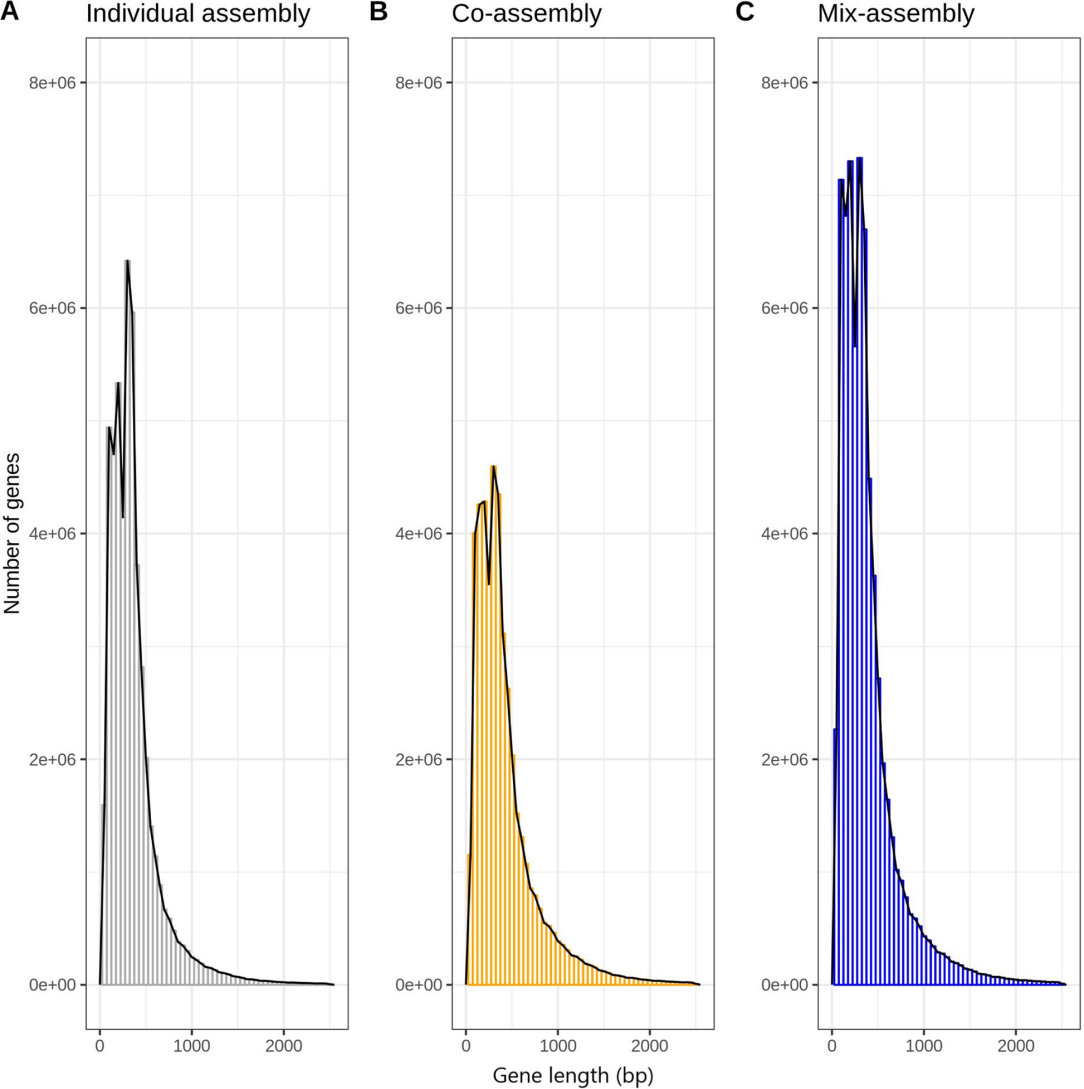
Gene length distributions of the three assembly approaches. **a** Co-assembly. **b** Individual assembly. **c** Mix assembly. Only genes ≤ 2500 bp are included in the histograms

**Fig. 2:**
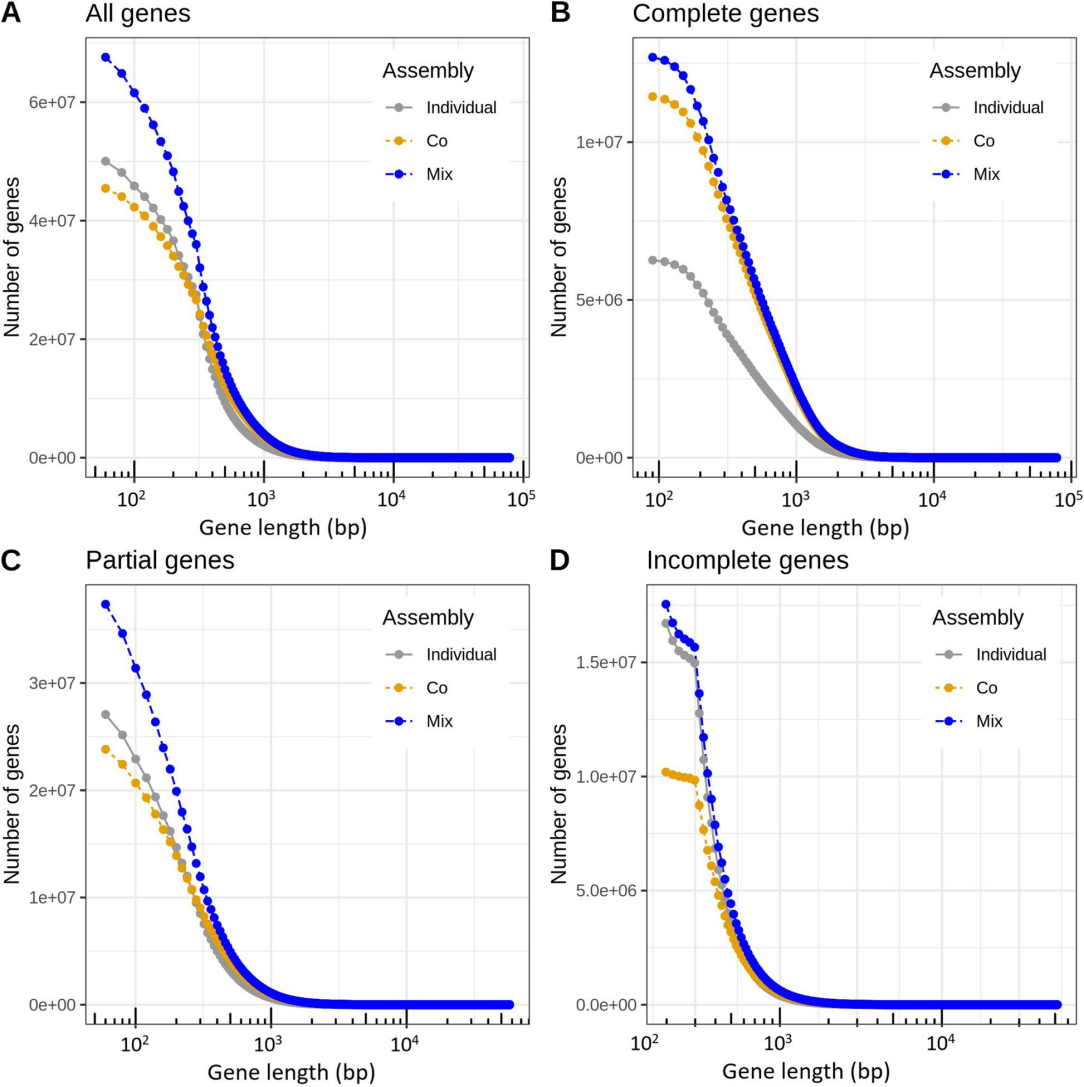
Cumulative distribution of gene lengths for the three assembly approaches. **a** All genes. **b** Complete genes. **c** Partial genes. **d** Incomplete genes. Complete genes refers to genes predicted to be complete (having a predicted start codon and a stop codon), partial genes to genes that lack either a start or a stop, and incomplete genes to genes that lack both start and stop. Gene length is given in logarithmic scale

The gene length distributions were fairly similar for the three approaches (Fig. 1), with peaks in the distributions between 300 and 350 bp. Co-assembly had the largest median gene length (336 bp), although mix assembly had the largest number of genes along the full range of gene lengths (Fig. 2).

Annotating the proteins against Pfam [23] gave the largest number of annotated genes for mix assembly (15 M) followed by co-assembly (13 M) and individual assembly (12 M), despite that co-assembly had a higher proportion of genes with annotation (29.4%) compared to the other two (23.0% for mix assembly, 23.8% for individual assembly; Table 2).

**Table 2:** Pfam annotation statistics for the different assembly approaches

Assembly approach	Total number of annotated genes	Number of annotated complete genes	Number of annotated partial genes	Number of annotated incomplete genes
*Individual*	11,930,617	2,422,526	4,751,188	4,756,903
*Co*	13,343,858	4,514,607	5,128,252	3,700,999
*Mix*	15,566,195	4,584,290	5,751,705	5,230,200

**Fig. 3:**
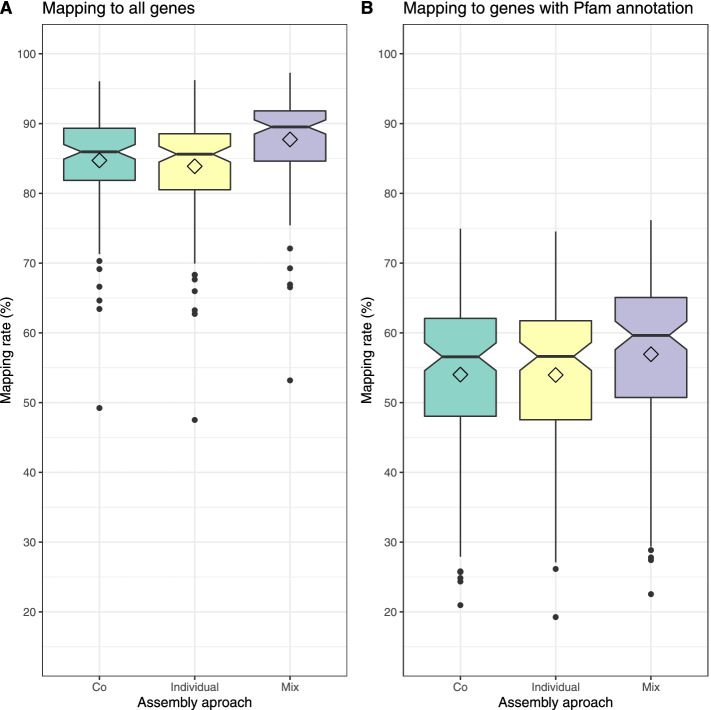
Read mapping rates to genes from the three assembly approaches. The boxplots show the distribution of mapping rate (% of reads) for the 124 samples, based on a random subset of 10,000 forward reads per sample. **a** When mapping to all genes. **b** When mapping to genes with Pfam annotation

**Fig. 4:**
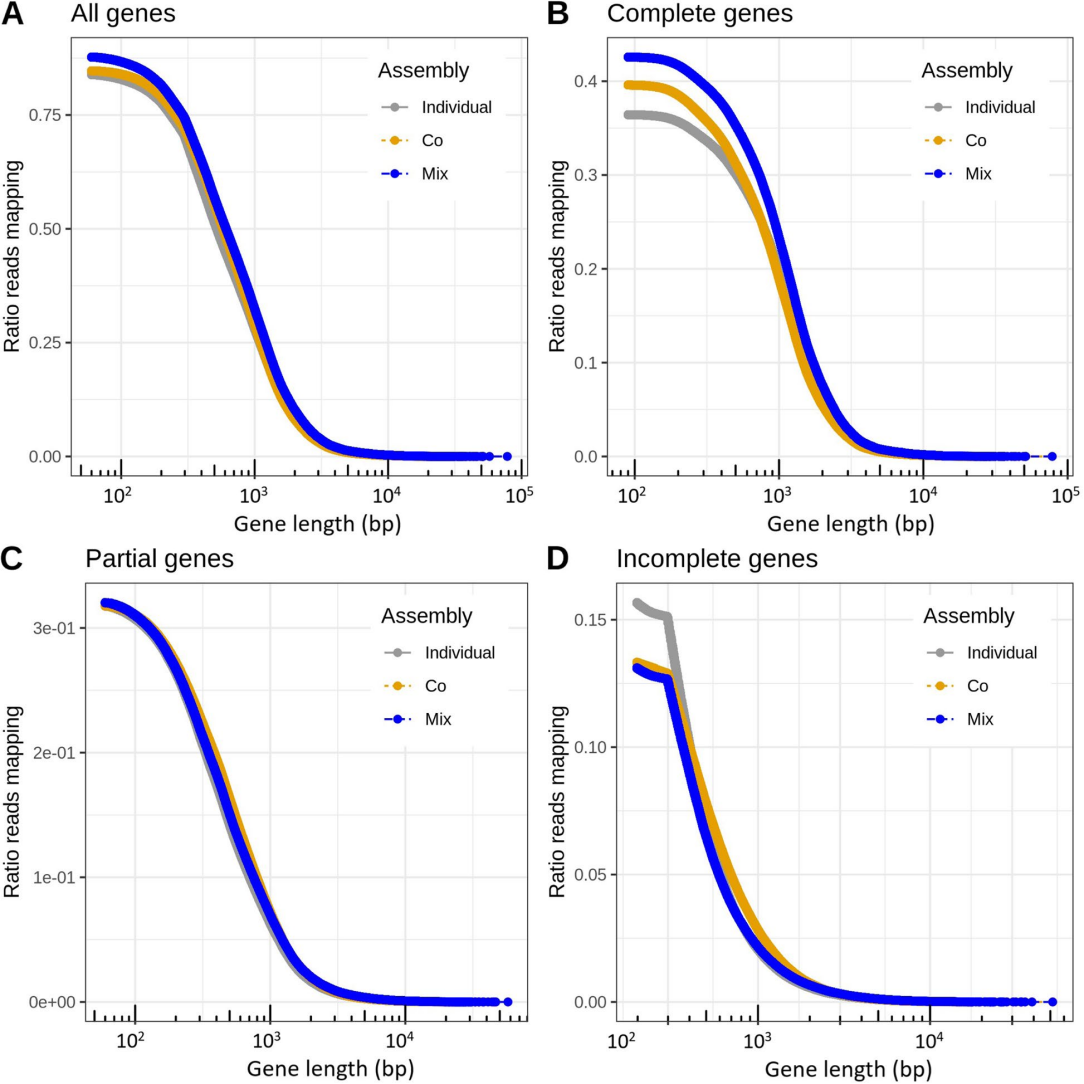
Read mapping rate as a function of gene length cutoff. The plots show the proportion of reads mapping at different cutoffs on minimum gene length. **a** All genes. **b** Complete genes. **c** Partial genes. **d** Incomplete genes. Complete genes refer to genes predicted to be complete (having a predicted start codon and a stop codon), partial genes to genes that lack either a start or a stop, and incomplete genes to genes that lack both start and stop. Gene lengths are given in logarithmic scale

Since biome-specific gene catalogues are often used as reference sequences for mapping of shotgun reads from metagenomes or transcriptomes, we further evaluated the gene sets by mapping reads from the metagenome samples to them. The average mapping rates for the 124 samples were 83.9, 84.7, and 87.7% for individual-, co-, and mix assembly, respectively, with numbers ranging from 47.5, 49.2, and 53.2% to 96.2, 96.1, and 97.3% for individual-, co-, and mix assembly. The mix-assembly read-mapping rate was significantly higher than the individual- (Wilcoxon signed-rank test, *P* < 10^−21^) and co-assembly (*P* < 10^−21^) rates (Fig. 3a). Figure 4 presents the cumulative mapping rate by gene length, showing the proportion of reads mapping at different gene length cutoffs. For all three assembly strategies, the highest fraction of reads mapping corresponds to complete genes, followed by partial genes. Of the three, mix assembly had the highest fraction of mapping reads mapping to complete genes (42.6%) and the lowest to partial (32.0%) and incomplete (13.1%) genes (see ‘Methods’ for definitions of partial and incomplete genes). Mix assembly also had the highest proportion of reads mapping to genes with a Pfam annotation (56.9%, *P* < 10^−21^), followed by co-assembly (54.0%) and individual assembly (54.0%) (Fig. 3b).

**Fig. 5:**
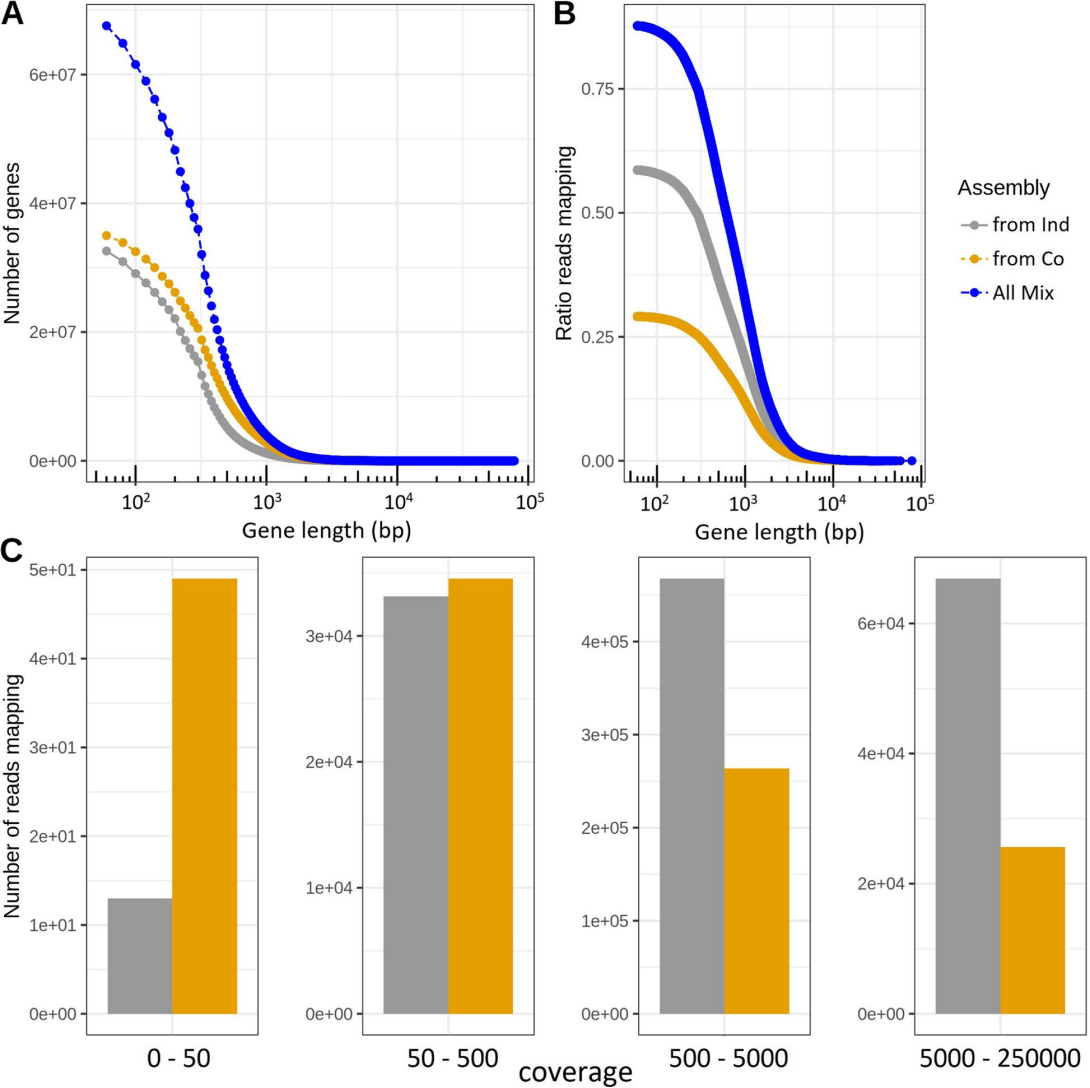
Contribution of genes from individual assembly and co-assembly to the mix-assembly gene set. **a** Cumulative distribution of gene lengths for the mix-assembly genes: for all (“All mix”) and for those derived from individual-assembly (“from Ind”) and co-assembly (“from Co”). Gene length is given in logarithmic scale. **b** Read mapping rate as a function of gene length cutoff. **c** Total number of reads mapping to mix-assembly genes derived from either individual assembly or co-assembly, for four bins of genes binned by their estimated coverage in the total metagenome (see “Methods”): low (0–50 ×), median (50–500 ×), high (500–5000 ×), and very high (5000–250,000 ×) read depth coverage

The contribution of genes from the individual- and co-assembly to the mix-assembly set of genes is shown in Fig. 5. A majority (52%) of the mix-assembly genes originates from co-assembly genes (Fig. 5a), representing 67% of the complete and 50% and 45% of the partial and incomplete genes, respectively (data not shown). However, among the reads that map to the mix-assembly genes, a larger fraction of reads map to genes derived from the individual assembly than to genes derived from the co-assembly (Fig. 5b). These seemingly conflicting results may reflect that mix-assembly genes derived from the individual assembly tend to be of higher abundance in the microbial communities than those from the co-assembly. This was confirmed by grouping the mix-assembly genes in low, median, and high coverage genes, where the majority of mapping reads mapped to genes derived from co-assembly for low coverage genes, but to genes derived from individual assembly for high coverage genes (Fig. 5c).

The mix-assembly gene set is significantly more extensive than the previously published Baltic Sea gene catalogue (BARM [6];) and may serve as a valuable resource for brackish water research. We compared the mix-assembly protein set with the Tara Ocean Microbial Reference Gene Catalog (OM-RGC.v2 [34]). Of the 67.5 M mix-assembly proteins, only 1.4 M were > 95% identical to Tara proteins, and, vice versa, of the 46.7 M Tara proteins, 1.3 M were > 95% identical to the mix-assembly proteins. Hence, the vast majority of the mix-assembly gene sequences are distinct from Tara genes. To increase the usefulness of the mix-assembly gene set, we removed genes potentially encoding ribosomal RNA and thus falsely predicted as protein coding (*n* = 16,804) and conducted taxonomic and functional annotation on the remaining genes. A subset of the genes (*n* = 70,223) was predicted to include encodings of other structural RNAs (in Rfam [33]), but we decided to keep these since they may also encode important protein-coding regions. The resulting gene set, which we call BAltic Gene Set (BAGS.v1), encompasses 67,566,251 genes, of which 31.0 M have a taxonomic affiliation (see Additional file 3) and 23.4 M have at least one type of functional annotation: 15.5 M with Pfam, 21.5 M with EggNOG [22], and 1.5 M with dbCAN [24] annotation (Table 3).

**Table 3:** Number of mix-assembly representative genes annotated using different databases

Gene completeness	dbCAN	EggNOG	Pfam
*Complete*	420,422	5,354,169	4,582,506
*Partial*	562,445	8,374,034	5,751,622
*Incomplete*	603,580	7,865,395	5,230,173
Total	1,586,447	21,593,598	15,564,301

Twenty-seven percent of the BAGS.v1 genes were predicted to be of eukaryotic origin. It should however be noted that the gene predictions were conducted with a gene caller for prokaryotic genes (Prodigal), and that a fraction of the eukaryotic genes has likely been imperfectly predicted.

## Discussion

Metagenome assembly is commonly carried out either by individually assembling reads from each sample [35] or by co-assembling reads from all the samples of a dataset [2, 6]. Here, the performance of these assembly approaches was compared. Although the number of genes was lower for the co-assembly, the total length (in number of base pairs) was higher than for the individual assembly. The two gene sets reported a similar mapping rate, although the co-assembly set had a higher number of genes predicted to be complete and a lower number of partial and incomplete genes than the individual-assembly set. In this study, we also proposed a new approach for assembly, aiming to combine the advantages of the individual- and co-assembly approaches, referred to as mix assembly. The mix-assembly strategy resulted in significantly more genes than the other approaches and also in the largest number of complete genes. It further gave the highest mapping rates and the greatest number of genes with a Pfam annotation. The reason why not only the number of genes but also the number of complete genes increased compared to the other approaches is likely because in the protein clustering process, the longest proteins were selected to form cluster seeds. Thus, if for example, an incomplete or partial protein from the co-assembly set forms a cluster with a complete protein from the individual assembly, the complete protein will likely represent this cluster in the mix assembly, since it is longer. Thereby, the clustering step that combines the two gene sets enriches for complete proteins. However, it may also to some extent enrich for artificially long proteins that may stem from sequencing, assembly or gene calling errors.

Analysing the contribution of individual- and co-assembly genes in the set of mix-assembly genes showed that genes with relatively low coverage (low number of mapping reads) in the samples were mainly stemming from the co-assembly. This likely reflects that co-assembly sometimes is able to recover genes that display too low coverage to be assembled from individual samples. On the other hand, genes with relatively high coverage were mostly originating from the individual assembly, which may be caused by the co-assembly sometimes breaking in such genes due to strain variation. If strain variation for such a gene is less pronounced in at least one of the individual samples, a longer fraction of the gene could be recovered in the individual assembly.

The 67 million genes of the mix assembly are based on 124 metagenome samples that span the salinity and oxygen gradients of the Baltic Sea and also capture seasonal dynamics at two locations [7]. This dataset (BAGS.v1) is a tenfold expansion compared to our previous gene set [6] and has the potential to serve as an important resource for exploring gene functions and serve as a backbone for mapping of meta-omics data from brackish environments. Consistent with our earlier study showing that the prokaryotes of the Baltic Sea are closely related to but genetically distinct from freshwater and marine relatives [35], only a small fraction of the mix-assembly genes displayed > 95% amino acid similarity to genes of the Tara Ocean gene catalogue. This implies that the Tara Ocean catalogue is not suitable for mapping of meta-omics data from the Baltic Sea and emphasizes the need for a brackish water microbiome reference gene catalogue. The gene catalogue BAGS.v1, including gene and protein sequences, and taxonomic and functional annotations, is publicly available at the SciLifeLab Data Repository, 10.17044/scilifelab.16677252.

## Conclusion

In this study, we have evaluated three metagenome assembly approaches for biome-specific gene catalogues. The mix-assembly approach, which combines assembly on individual samples with co-assembly on all samples, outperformed the other two approaches in terms of number of nonredundant genes, number of complete genes, mapping rates, and number of genes with a Pfam annotation. Hence, the mix-assembly approach represents a feasible approach to increase the information gained from metagenomic samples.

## Supplementary Information

**Additional file 1.** Map with sampling locations. The marker colour shows the salinity of the water sample and its size, the sampling depth. The contour lines indicate depth with 50 m intervals.

**Additional file 2.** Table with brief description of sampling and sequencing. For detailed descriptions, see references in the table.

**Additional file 3.** Interactive chart of the BAGS gene set taxonomic affiliations.

## Data Availability

The shotgun reads and individual sample assemblies have been published earlier [6, 7, 12]. The co-assembly contigs and the mix-assembly gene set (BAGS) together with annotations are available at the SciLifeLab Data Repository powered by Figshare, 10.17044/scilifelab.16677252. The contigs for the individual assemblies were published earlier [7] and are available at ENA hosted by EMBL-EBI under the study accession number PRJEB34883. When using the BAGS gene set in your work, please cite Alneberg et al. (2020) [7] in addition to this study.

## Abbreviations

BAGS: Baltic Sea gene set
LCA: Lowest common ancestor
