# Supplementary figures and images for: Evaluating metagenomic assembly approaches for biome-specific gene catalogues
Source: Microbiome. 2022 May 6;10:72. doi: 10.1186/s40168-022-01259-2 (PMC9074274; doi:10.1186/s40168-022-01259-2)

# Sample group

- Transect 2014
- Coastal 2015
- △ Redoxcline 2014
- ⊠ Asko 2011
- ◈ LMO 2013–2014

# Depth

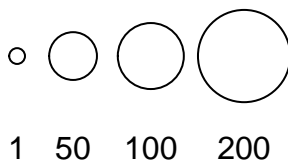

# Salinity

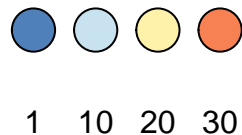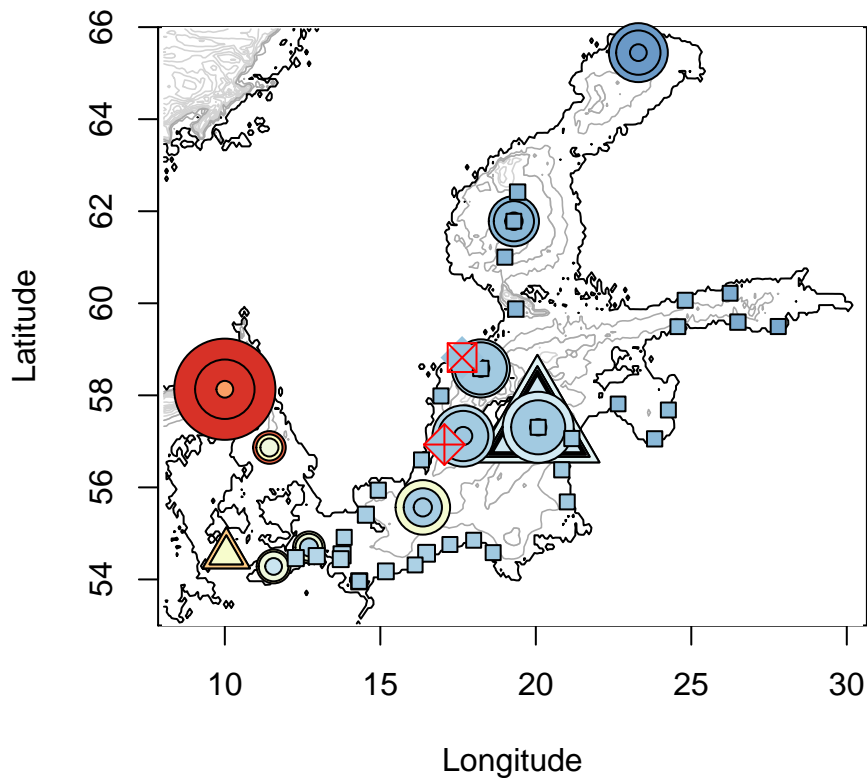

Supplement: Supplementary file 2 — Additional file 1. Map with sampling locations. The marker colour shows the salinity of the water sample and its size, the sampling depth. The contour lines indicate depth with 50 m intervals. [file 40168_2022_1259_MOESM2_ESM.pdf]
