# Supplementary material for: Evaluating metagenomic assembly approaches for biome-specific gene catalogues
Source: Microbiome. 2022 May 6;10:72. doi: 10.1186/s40168-022-01259-2 (PMC9074274; doi:10.1186/s40168-022-01259-2)
Supplement: Supplementary file 3 — Additional file 2. Table with brief description of sampling and sequencing. For detailed descriptions, see references in the table. [file 40168_2022_1259_MOESM3_ESM.pdf]

Additional file 2. Sample retrieval and sequencing description
(further sample description in references)

Sample set name	Sampling period	Number of samples	Pre-treatment	Average Reads pairs (10^6) (after pre-processing)	Ref.
Askö 2011	Surface water samples collected on six occasions between June 14 and August 30, 2011.	24	All samples were sequentially filtered through 200, 3, 0.8 and 0.1 µm. The four-size fraction obtained were sequenced separately.	36.8 (884,286,698 read-pairs)	(12)
Redoxcline 2014	Samples collected on October 18 (2 samples, Gotland deep), October 26 (8 samples) and, September 23 (4 samples) 2014, from the Boknis Eck station.	14	Two samples collected Oct 18 were captured on 0.2 µm filter without pre-filtration. 6 samples were filtered on 3.0 µm without pre-filtration and 6 samples were filtered on 0.2µm filter using 3.0µm filter for pre-filtration.	19.9 (277,915,293 reads-pairs)	(6)
Transect 2014	Samples collected from June 4 to June 17, 2014 at 3 depths at ten stations from oxic zones (from 2 to 242 m depth), within the salinity gradient of the Baltic Sea.	30	Samples filtered on 0.2 µm filter without pre-filtration.	58.7 (1,780,576,171- read-pairs)	(6)
LMO time series 2013-2014	Surface (2 m) water samples collected at the Linnaeus Microbial Observatory (LMO) station east of Öland from January 23, 2013 to December 16, 2014.	22	Samples filtered on 0.2 µm filter after pre-filtered on 3.0 µm filter.	26.2 (576,748,368 read-pairs)	(7)
Coastal Transect 2015	Surface water (1.7 - 4 m) Samples collected in August/September 2015.	34	Samples filtered on a 0.2 µm without prefiltration.	56.0 (1,903,982,192 read-pairs)	(7)
