# Supplementary material for: Evaluating metagenomic assembly approaches for biome-specific gene catalogues
Source: Microbiome. 2022 May 6;10:72. doi: 10.1186/s40168-022-01259-2 (PMC9074274; doi:10.1186/s40168-022-01259-2)
Supplement: Supplementary file 4 — Additional file 3. Interactive chart of the BAGS gene set taxonomic affiliations. [file 40168_2022_1259_MOESM4_ESM.html]

Javascript must be enabled to view this page.

magnitude
magnitudeUnassigned

file\_for\_krona\_clean

67566251

4781141
18433258

50

50

4
50

20

23

3

3

84984
1973948

7351
642340

3953
613437

82

82

72

1
72

68

1

2

10

10
1

9

602851
12286

3451
7

1
3367

4

4

4

4

4

4

3362

3361
4

3351

7
3351

2722
3342

620

1

1

1

6

6

3

3

1

1

1

1

50
6

9

9

9

9

9

1
24

2
13

2

1

1

1

1

1

1

1

1

1

1

3

1
3

2

5

1

1

1

4

4

4

4

10
2

2

2

2

2

5

1

1

1

1

1

1

3

3

3

1

1

1

1

11

10

9

4
7

1

2

1

1

1

1

1

1

1

1

1

1

1

27

21

21

21

21

21

6

6

6

6

5

1

34
2

3

3

3

3

3

29
2

12

12

12

12

12

15

15

15

15
5

1

9

577545
3276

2982
3

10

10

4
2

2

2

1
6

1
4

3

1

1

2964

2964

2964
13

2929

2

1

1

3

5

1

1

8

5

5

5
3

1

1

144
571287

365
6

227
41

4

3

3

3

1

1

1

8

6

6

6

2

2

2

3

3

23

16

15

1

14

1

1

7
2

4
5

1

139
13

5

5

32

32
4

5

5

5

2

2

2

4

1

1

3

3

3

3

4

2

2

2

2

2
13

11
7

1

1

3

1

1

1

1

82
56

3

3

3

10

10

10

10

1

1

1

9

7

4
1

1

2

3

3

3

2

2

3

3

3

1

2

6

2

2
1

1

1

1

3

2
3

1

1

1

8

8

3
1

1

1

1

1

5

5

5

1

1

1

1

1

9

9

9

9
3

3

3

122

116

116
1

111

111

4

4

6

6
1

1

1

1

4

4

3

1

570778
3792

10347
564727

7787

7783

7
7783

7776

4

4

4

7

7

1

1

6

4

2

1112

1112

1112

1112

43582
545474

669
65216

2

2

2

2

896
60732

516
54752

2406

2405
505

592

589

589

589

3

3

3

3

1
1308

1307
3

1304

1304

1304

1

1

1

5573

2
1

1

1

4

4

4

4

4
1

1

1

2
1

1

5563
640

399
1342

8
528

2

518

2

2

413

3581

3581

244
46257

4

1

1

1

1

2

2

43960
2837

614
22806

13196
372

1660

1658

1658

3

1655

2

2

32

1
32

4

4

4

2

2

2

4

4

1

1

2

23

22

22

22

1

1

1

11132

11132
1263

7243
1161

1

1

3

3

3225

2

1

3222
614

935

1673

2853
773

562

477
1

476

532

509

2625
1

4

1

3

2620

23
2620

2597

1

1

1

41
8

4

4

4

4

1

3

29

29

29
1

1

1

1
27

1

25

8955

8955

8955
509

923

923

2738
7523

875

875

323

190

344

1

369

1

299

321

858

605

599

18316
700

4

4

1

1

3

3

10194
503

6

6

6

6

4175

4175
293

1

1

1

26

26

26

8

17

1

2

2

2

2590
1

7
2589

2581

1

1263

1263

1262

1

5510

5510
457

684

684

684

3

3

3

1925

2

2

768
1923

496

1

658

1

1

1

1

2440

361
2440

1094

1094
801

293

985

2

2

2

2

5392

5392

5392

5392

2024
3

85

85

84

82
16

66

2

1

1

1935

1935

1935

1932

3

1

1

1

1

1

1

16

16
5

2

2

1

1

1

1

1

1

1

8

2
8

1

4

1

1

1

1

1

1

2

2

1

1

1

2027

2027

2027

2027
1213

814

4

3

3

1

1

3
931

1

1

1

927

1
927

923

923

3

5

3

1

1

2

2

1
2

1

1

26
4125

12

3

3

3

3

9

8

8

1

1

24

15
8

5

1

3
4

1

1

1

1

1

9

9

1
9

5

2

1

2

2

2

1
1131

1
8

3

3

1

1

1

1

2

2

1117

1117

1117

4

4

4

1

1

2

1

1

1

1

13

7

6

6

1

5

1

1

1

6

6

6

1

2

3

2915
108

17
14

1

1

1

1

1

1

1

1

1

1

2

2

2

797

797
3

794

257
1989

12

12

1

1

1

9

20

8

1

1

7

7

12

1
12

1

8

2

1074

1074

1074
6

1068

3

3

3

3

623

623

2

2

621
3

618

2

1

1

1

1

1

23

17

1
10

2

1

1

1

2

3

1

1

2
3

1

3

3

5

1

1

2

1

1

1

1

1

1

3813
27

4

3

3

1

1

2

2

2

2442
79

1285

1285

1285

14

3

3

3

8

8

8

3

3

3

2

2

2

1062

1062

1062

1

1061

2
3

1

1

11

11

11

1318

1318

1318

6
2

4

4

997

1
14

3

1

1

1
2

1

5

1

1

1

1

2

2

2

1

1

5

5

5

969

964
9

3
955

952

952

4

4

4

1

1

1

14

8

8
7

1

1

1

2

2

2

4

2
4

1

1

74
5164

1

1

1

1

1

1

1

1362
1

6

1

1

1

5

4

4

1

1

392
1355

497

497

466

1

465

2672

1
12

2
8

1

1

4

3

3

2

2

2

2658
80

2083

2083

1

1

1

1

493

493

3

2

2

1

1

1

1

1

1051

1051

1051

780

777

777

777

2

2

1

1

1

2

2

429727

429727
4

2

1

1

1

1

1

1

429721
29097

254040
7560

733
45414

1

1

1

1

18353

2
4

1

1

1

1

18342
602

546

546

546

5660

5660

5660
260

4983

417

1

1

1

9973
231

7635
1809

168

343

63

24

60

28

210

4055

183

25

41

33

38

60

48

42

304

101

2107

2107

236
1560

875

875

449

449

3

3

3

2

2

2

2

2

2

5058
35

1

1

1

1

1

7
1

1

1

1

1

5
4

1

3145

3145
660

428

428

428

1371

1371
1

3

1366

1

686

686

686

686

3
1870

1866

1866

1

1

2680
76

1
1185

2

2

2

1
1182

1181
762

4

184

127

2

101

1

2
672

3

3

667

667

1

1

737
5

6

6

5

5

721

721

721

721

9

9
7

2

315
18512

473

473

473

1

16938
2016

358
7290

664

664

137
5935

1

3
5

2

5467

4
323

318

1

2

333
2

331

181
2756

737

737

1838

1838

437

437
6

431

1

1

1

2

2

1

1

551
3762

1

1

1

1

1

450

450

2759
1025

290

252
691

439

384
434

50

313

1
5

4

1

2

2

2

7

1

1

2
6

1

3

664

99
664

274

291

1

1

1

782

782

782
3

779

3

3

3

77
1

21
76

7
10

1

2

3

3

8
12

3

1

16
24

2

3

2

1

2
6

3

1

197358
4180

221
16122

5898

5898

5898

5898

1
701

3
694

691

2
6

2

2

10
4

4

4

2

2

2605
530

1228

1228

553

553

292

292

2

1

1

216
6686

309
1937

2

2

2

1

1

434

1

1

433

433
5

416

416

2

10

1191

1

1

1190
4

1179

3

1

3

194
4533

2151
711

2

2

968
148

545

275

470
39

272

2

157

2188

2188
732

101

209

121

2

1

1

255

255

312

454

1

1

1

1

1

1

1

1

120
59915

1

1

1

1

1

9

2
8

6
2

4

1

1

1

810
4

1
806

805

2

1

1

11

11

11

1
4

3

2

1

4
1

2
3

1

3145

419
3145

1786
337

553

2

1

34

223

1

634

1

940
197

271

471

1

7

6

5

5

1

1

1

1

130
55799

2

2

2
1

1

1

1

8576
217

518

518

518

7260

7260

7260

563

563

563

10

10
4

4

2

7

7

7

1

1

1

47091

47091

47091

47091

1

1

1

216
8124

690

690

690

531

531
1

530

185
5133

1

1

2475
505

1252

718

2472

2472

2
1551

1545

1545

4

4

3

3

1

2

104601

104601
214

1000

1

1

1

1

1

1

998
2

3

2

2

1

984

984

1

983

8
9

1

1

1

1

1

103383

103383
877

2566

2566

2566
1

2564
1329

119

232

884

1

1

1

79627

79627

6

6
4

2

1
79621

79620
567

377

78676

20313
400

1

1

10251
235

2

2

2

441

441

441

9565
809

1

1

1305

686
1305

227

392

4846
287

1
1017

1016

1674

1673
1868

195

1

1

1465

1465

1

255

255

882

882

8

8

8

9661
15

275
9486

239
8764

2674
466

642

593

973

8
5851

22

5821

2

2

427

427

427

1
17

16

16

1

1

1

160

157

157
1

1

155

3

3

3

2
3

1

1

1

818

817

4

4

813

813

1

1

1

757

757

757

757

2840

1

1

1

509
2836

3

3

3

2

2

2

511

429
511

30

30

26

26

5

5

5

181
1458

1
3

2
1

1

1

1

1

1081
159

750
133

616

616

1

172

1

1

191

191

348

348

348

1

1

1

2

1
2

1

1

3708

3708

3708

3708

753
3708

68

2887

110
9301

6

2

2

4

4

1893

1
1886

2

1

1

1

1

1

1

1883

1883

2

1

1880

2

2

2

5

5
1

1

3

9

1

1

7

2

1

4

1

1

2

1

1

1

1

1

1

1

1

1

2

1

1

1

1

15
3

6

6

2

2

3

3

3

1

1

3

1

1

2

2

1

1

1

1

1

1

1

21

2

2

2

19

19

5

5

5

13

1

1

12

12

1

1

1

293
6861

1

1

1

1

3957

3957
4

3953
6

3947

1

1

1

1

167
2608

1516

2
1516

1514

925

925

925

1

1

1

2

2

2

363

360

360

3

2

1

1

9

9

1

1

1

2

2

2

5

2

2

1

1

1

2

2

1

1

1

1

1

1

1
15

1

1

1

5

3
2

1

2

2

2

2

1

1

5

3

2

2

2091
136269

70
93497

1393

1393

1392

26
1392

14

12

11

11

1

1

1

1

1

1

1

1

1

1350

3

3

3

1
1347

1346

1346

1346
35

1311

1

1

1

1

1

1

1

18

1
16

1

1

1

1

7
1

5

1

4

4

2

2

2

2

1

1

14

92002

2
20

2
5

3

5
1

2

2

2

2

2

3

3

3

3

2

2

2

2

2

2

1

1

1

91979
711

1

1

1

318
88912

205
3342

1

1

1

1896

1
1896

1895

1240

1240

1240

3

3

3

3

85248
284

83353

83353

83353

3
1611

1606

1606

2

2

1

1

1

1

1619

1619
2

1611

1611
613

4

380

614

1

1

1

1

1

1

4

4

1

3

6

6

6

6

730

6
730

6

6

6

1

1

1

1
717

2

2

714

714

714

714

1273

146
1271

458

458

667

667

2

2

113
4856

2

1
1718

475
1717

198

198

1044

2

2214

1
2214

1705
2213

508

3

3

1

2

2

2

13

13

2

2

11

1

1

1

1

789

1

1

1

10

9
1

4

3

1

3

3

1

1

1

1

1

778

778

778

778

34552
482

8

8

8

4
8

2

2

1

1

1

1

550
22224

550

550

548

2

888

1
885

884

884
6

878

878

3

3

2

1

922

922

921

1

1
543

19
3

1

2
15

4

6

3

1

1

1

1

1

1

521

521

521

521

1

1

2

1

1

1

1

1

1

3
5

1

1

1

1

1292
1

1279

1279
3

1273

3

8

1

1
3

1

1

1

1

1

3

3

2

1

1

1

1

2

1

1

1

1

13478

13477

13477

1

1

1

13476

13476

13476

1

1

1

1

1

1

5

3

2

1

2

2

3

2

2

1
2

1

1

1

1

1

2

2

1

1

1851

7

7

1

4

2

1

1

1

1839
1

1827
1

1823

1823
19

1770

3

1767

1767

34

1

32

1

2

2

2

1

1

7

2

2

3

1
3

2

1

1

1

1

1

1

4

2
4

2

4

4

4

4

18

1

1

1

1

1

1

1

1

1

7
3

4

4

8

2
8

1

2

2

1

5
2114

2097

2097

12

11

1

2
1460

14
1

2

2

2

2

2

11

9

9

4

1

1

3

2

1
1426

9
2

3

3

3

1

1

1

3

3

3

3

1411

1
1411

1410

1410

889
1410

148

250

1

122

5

3

3

3

2

1

1

1

1

18
1

5

1
5

3

2

2

1

1

1

1

1

1

1

11

1
11

1

1

1

1

1

1

8

8

8

10373
137

7259
561

2
4

2

2

237
5184

1

1

1

3

1
3

2

2012

2012
726

360

479

447

2931

2931
346

1

1884
122

51

1671

40

4

1

1

1

2

251

440

1

1

1

1

1

1

1

1508

1508

724
1508

356

232

1

1

1

1

2

113

76

1

94
2977

2

1

1

1

1238

1238
1

1232
2

3

1

2

1226
590

94

542

1

1

2

2

1

2

2

1

1

1

1642

697
1642

1

363

562

19

5

1
5

3

3

1

1

999

1

1

1

1
991

1
10

9

973

971

2

1
7

4

2

5

5
4

1

2

2

1

1

1

2

2

2

2

4

4

4
3

1

9
2259

15

15

15

15

15

15

8

8

4

4

4

4

1

1

1

1

3

3

2
3

1

2223

66
2223

7
1016

27
69

1

1

11

1

3

3

7

7

15

11
15

4

1

1

1

1

1

1

1

1

3
8

2

2

1

1

1

1

1

1

1

116
2

10

10

1

1

102
2

1

99

1

2

2

2

814
170

571

571

73

73

1140

59
1140

1

1

1

1

820
11

408

394

7

1

1

10
257

183

1

178

3

1

28
64

2

7

4

10

4

1

2

6

1

1

4

4

1
4

2

2

2

1

1

1
9535

5
9522

2

2

2

1

1

1

1

1

1

1

1

1

9514
9

15

7

7

7

2

3

2

5

5

5

5

2

2

2

1

1

1

1

9451

9451

9451

1

1

9450

9450

37

5
31

23

11

11

11

12

12

12

3

3

6

4
6

2

2

2

2

2

2

2

2

5

5

5
4

1

1

1

7

7

7

7

7

7

6337

6337

10
6337

1
6306

1

6295

9

21

2

19

214

214
14

173
1

40

13
7

1

5

13

13

14

2

12

2
132

1

1

74

74

29

29

9

1

8

14

14

3

3

2
27

3

3

2

1

10

10

3

7

1
12

2

2

1

1

1

1

4

3

1

3

3

69

69

69

69

69

21400

21400

3
21400

21397
117

69

21211

83

2

2

81

81

81

81

1242630
55546

4324

4324
4

4320

4320

593644
1847

566509
5025

798
229556

124359

124359

104399
8162

49161

15427

15427
12244

3183

31649

24

24

8667
331904

5
43472

43454

13

279765
67650

27348

184757
159517

25240

10

10

24

24

24

1

23

23

25264
660

26

26

26

15117

15117

15117

9461

9461

9461

38203
308268

155154
581

176
3

81

1
81

1

1

78

78

1

1

1

8

8

5

5

3

3

84
21

8

8

8

2

2

2

21

21

1

1

20

32

2

3

3

3

27

27

4

4

4

4

4

79

3

3

2

2

2

3
50

20

27

24

24

24

12

12

12

12

2

2

2

2

154300
8779

117246
26632

2

2

1

1

1

5

5

4

1

20257

20257

20257

8691
472

3

2

2

1

9

9

11

11

7

1
4558

2

4552
30

4522

1

2

3626

3626

5

5

303
55254

33

33

31

1

1

4636
52644

19

24828

312

14072

4903

4

10

154

1

6

1

10

2

134

21

1

1

3

3680

4
31

13

7

7

7

9
2138

1

2128

69

69

3

3

19

19

14

12

2

18

18

415

415

415

3529

2

2

3476
37

35

3333

2

69

51
1

50

7

7

7

1
2423

2403

2403

4

3

1

15

15

13

13

13

2112
28275

10

10
4

1

3

2

49

20
49

20

9

2

2

2

13066
1841

5

5

6926

6926

4

4

4287

4286

1

3

2

1

7

2

2

5

5

9

9

9

1

1

1

292
6

67

67

60
9

30

21

9

9

143

5

5

2

136

7

7

4

4

12704
2543

6162

6158

6158

1

3

1

1

54

54

54

3930

3930

14

14

12

12

12

7
1

2

2

4

4

87354
11628

11

1
11

2

2

8

8

16073

19

19

17

17

2

2

2

34
1

9

9

19
2

16

1

5

5

5

5450

259

259

12

5179

12
10404

10316
424

13

9

4

45

3
9834

9831

3

3

73

2

66
71

5

147

147

147

78

77

1

5561

14
5561

22

22

17

5

6

6

41

41

5473
2

3

5468

5414

54

3

3

2

2

53961
2

26
2

13

2

2

11

11

11

11

11

53933
11131

94

94

26
1

25

36

36

10

10

5758

4

5754

1624

1624

12

1612

31
1

22

5

2

1

3

2

1

17517
35206

5380

5375

1

1

4

12309
2122

26

5932

4207

12

1

1

5

5

10

14

14

3

3

39

7
33

13

13

3

3

3

10

10

6

6

1

3

2

27557

27557

5
27557

6

6

27546
6167

5494

4436

6

4430

3883

7566

5281

5281

5281
1

6
14

1

7

5266
7

5243

1

4

4

2

9

12
2603

9

9

9
3

6

3

3

3

3

3

1
2190

12

2
12

6

1

5

4

4

4

2001
180

438
1448

414
107

153

37
153

17

80

19

66

14

14

1

1

1

16

16

35

31

4

75
10

32

32

15

15

18

6

4

2

12

13

4

4

9

9

583

583
373

42

3

11

54

11

64

7

18

13
1

8

8

4

348
13

184
3

158

158

6

151

1

3
20

4

10

3

3

1

1

2

2

8

8

8

9

9
6

2

1

134

16

16

118

118

118

25

25

25
2

4

4

19

165
2

3

2

2

1

1

1

160

160

1

159

11

6

6

4

2

1

1

1

3
4

1

224
13

1
135

3
134

26
123

14

25

4

7

7

1

7

3

1

7

28

6

6

2

2

17
63

5

5

5

11

11

11

20

2

2

10

6

4

8

8

6

6

6

3

3

3

1

1

1

1

13

13

13

165

165

79

79

1

1

39834

39834

39834

12
39834

44

39755

23

1
4758

25

25
23

2

2

4732

4732

4732

4717

15

15

73
1

24

24

24
6

4

14

5

5

43

43

43

43

43

3767

3767

3767

3767

3767

224452

224452
1109

222776
47

222647
25007

1

87855

87852

3

109784

109784

82

1

1

81

567

567

567

3994

3994

3994

3994

3994

3994

8369

8369

8369

8369
15

8354

906
33881

8627
29

7062

7062
252

3443

3443
265

892

2286
1853

210

46

57

120

3367
102

130
1136

1005

1

1

1

1

2128

2128

2128

8

7

7

1

1

1

1528

1517

1517

3

3

8

8

8182
25

115

115

114

1

6
8042

4

4

8014

8014

8014

15

3
15

3

3

9

3

3

3

3

1304
16166

1
8289

6

6

6

1

1

1

1

8281

112
8281

8104

65

65

4

3

3

3

1

1

1

1

2

2

1

1

1

1

1

1

2

2

1

1

1

1

1
6562

7
6561

3
6551

1

6547

1

1

2

2

74

2

2

7

1

30

5

2

14

10

1

4432
98032

173

173

173

307
34011

4

4

3

3

1

252
23289

13873

13873

1413
13873

1

1

16

6528

5914

9164

1
9156

17

9127

9127

11

8

8

10411

10411

2

2

2

10409

63
10409

4

2

20

20

10320
192

10128

7792
3

7666

7647
8

5

5

5

5

5

3735

3735

2
3735

3733

3899

3899

3899

3899

5

5

5

5

14

7

7

7

7

1

1

1

1

6

6

6

123

123

123

51624
1304

585
18244

6717

6716

6714

6714

2

2

1

1

5

5

5

3

3

3

3

10934

10934

24351
63

46

45

45

45

45

45

1

1

1

1

4969
24241

10680
898

2658

2658

12

1
2646

2645

7124
658

2883
1

2881

2881

1

3566
1

8
3563

3555

2

17

17

15

15

8577
527

7

7

5732
2

970
5730

2395

2364

1

4
2311

2265

2265

1

1

41

41

1

1

1

1

7725

7725
73

7427

7427
2450

6

6

582

582

3

2780
218

2562

291
1606

18

3

1294

1294

225

225

225

662

34

34

23

23

421

421

421

184

9

175

175

4135380
206320

2092136
27174

21618
99368

3

3

3

3

32257

32256
4834

19161
7731

2

582
11

571

1971

1101
3

1098

797
2

795

1

2

294

1473

1535

673

732

738
1655

48

528

247

24

70

612

573

573

2
598

596

2228

6

2222

3026

3026

3026

1
1836

1835
2

1833

1

1

1

36270

9690
36270

2359

2359

4932
18064

3037

4036

2207

3852

1702
3813

1074

1074

1036

1036

1

2344

2344

6714

6714
596

172
4253

1

4

2155

1921

2

1

1

1
1859

1857

1857

1

4

1

3

20

20

20

20

2486

2486

2486
758

890

890

838

3

3

3

67730
279

73
21187

150
15710

9

9

8407

8407

7144

7144

7144

5404
177

6

6

6

5152

1083
5152

976

386
3093

1374

1333

1333

69
4

15

15

15

50

50

1
46264

46261

46261
13650

10383

10383

7877

7877

33

9

1

3

5

7

17

3908
27

3869

12

12

10410

36

36

10374

2

2

1897860
54443

55498

55498

1
55498

55497

209

55288

4857

4857

4857

11426

11426

1272
11426

4299

4299

5855

5855

1132795
97334

56344
2829

547
20081

2

2

2

2

2

13003

13003
937

5825

5825

6241

6241

13

13

13
2

1

10

2589

2589

30

30

2559

2559

3927

1
122

118

112

6

3

3

3805

3805

3805

16387

16387

744
16387

3

3

13567

13567

2073

2073

24

24

24

24

17023

17023

17023
1655

8589

8589

16

16

6763

6763

129337
13

9

9

9

9

9

129315
3462

3

3

23

23
2

3

3

3

18

18

2

2

2

2

609
12912

3026

3026

11

11

992
3015

2023

1261

1261

1261

5180
35

1193

1193

3

2

1

27

27

20

20

3902

2

3900

20

20

20

20

10

10

10

2806

2653

2653

153
9

130

14

108476

108476
3037

3592

3592

14188
872

3712
35

3677

9604

38857
22

38804

31

48775
1050

16

41860

264

117

2017

2111

71

1269

3
27

17

1

3

3

7

3

6

1

14

14

14

14

4423

4423

4423

4423

2

2

142049

142049
2726

4422

4422

4422

4422

10173

10173
133

8469

8469

1571

1571

3199

32

32

32

3167

3167

11

11

3156

100558
3

41

41

41

49
100514

2998

2998

18

18

97449

97449

7410

7410

7410

7410

13561

606
13561

8814
1196

3672

3946

4141

4141

707729
10340

448779

42683
448779

37

37
8

29

51359
193553

13390

13390

562

562

118817
22793

6496

2

1

1

6926

32544
32549

5

26843

21299
21292

7

1909

16

16

9409

9409

212506

212506
119396

23

37101
4601

24740

7760

36667

4589

14730

7891

7891

21
7891

88
11

5

70

2

26

26

7756
2849

4097

757

53

53

209044
1691

8203

8203

8203
25

129

8049

199150
255

5051

1
5051

63

4986

1

1

1
193784

193783
9419

9

1

2

200

25

4368

1

7071

162625

9948

114

60

60

60

232
20017

8933

14

14

14

8919

8919

8919

6035

6035

6035

4817
8

77

77

76

1

1

4732

4732
4

4651

77

11658

11658

11658

837
11658

7262

3559

627

627
115

138
465

46

6

2

2

38

47
281

14

17

147

56

47

47

47

87
52125

137

137

137

137

7373
51901

11684
6

4

4

11674

6150

6150

7647

7647

19047

19047

20193
281

3059
7

3052

3052

3052

3052

106
16853

44
16649

16559

16559
6685

6061

6061

3813

2
6

1
2

1

1

2

2

2

2

2

11

9

9

1

1

1

1

27

22

22

5
4

1

28
10

2

2

2

2

2
1

1

4
14

1
6

5

4

4

9
34

15

15
13

2

4
10

4

2

2

1

1

1

1

1

1

1

1

35

35
5

30

27

3

31025
2

31023

3768
31023

3097

3097

10208

10208

13950
1

4
3

1

13945

298829
21305

224854
21030

117592

117592

1

1

86231

86230

1

1

23548

23548

23548

14373

14373

14373

14749

14749

14749

147003
7

1925
146996

6262

799
2

566

566

231

5462

5462

1

1

138798

44805
138798

48241

12798

32954

2

2

2

9

9

9

165
89039

11

5

6

88851
654

65771
88061

91

91

91

12123

12123
7

12116

10076
159

9597
7532

2065

14

3

11

78

21

186
207

20

1

131
13

9

9

109
108

1

5

5

5

12

12

12

1

1

19106
1692554

684658
11652

13703
652033

1302

1302

1302

10

10

1292

1292

13

13

13

13

13

13

565068
780

466

466

1

1

1

465
35

143
10

13

120

15
287

54

1

107

37

20

36

17

7

10

563822
2027

561544
2

561540
417286

1

1

76306

1
76306

8

76291

6

67947
176

2
163

114

47

67531
16

67515

75

75

2

2

1

1

1

1

1

1

1

1

73

42

42

42

31

31

28

28

3

178
2

2
43

4

4

4

11

11

1

7

3

26

23

3

3

115

115

115

13

1

1

12

12

4

8

5

5

5

8

8

8

8

8

18378
62936

6

6

4

4

4

2

2

2

4211
23505

15574

15574

84
15574

11

2

5

1001
15471

14470

1

3720

3720

9
3720

3711

12621

12621

38
12621

5

2

12553

7

7

14

1

1

8426

137
8426

2

2

2

8186

8186

8186

28

28

28

72

72

72

1

1

1

2
9003

8928

4

1

1

1

3

3

3

1

1

1

1

8923

8923

8923

8923

73

72

72
2

3

3

2

2

11

11

54

54

1

1

1

1

1

2

1

1

20917

20917

20917
37

1

1

1

6

2
6

4

20853

20853
1

20852

10

10

10

10

10

10

54

54

54

4

1

1

1

3

3

3

641
28632

3

3

3

17851

17851

17851
11960

5891

3

10134

10134

8445
10134

1689

17780

17780

17780

17780

17780
6312

1606

487
1426

939

4619

4619

3817

916104
186886

2555
184108

1

1

1

95245

95245
9922

11104

33086

1

33085

8306

32826

1

177
63490

3094

3094

60219

60219

33

33

33

22541

22541

22541

243

243

242

1

116

5

5

5

5

111

111

111

1

5

20

85

5118
156716

86534
875

3505
19035

7431

8099

6684

6684

3
4

1

2

2

20

20

2

2

59908

59908

4

1

1

3

65064
1717

15278

15278

7
48069

3

13

48046

8477

8477

8477

8477

172952
1952

62
27936

16286

16286

11586

11586

2

2

56
143064

8

8

1

1

1

1

485
142999

1270

5

4

1

141239

22104

22104

22104
14

2

2

22074

9

3

6800

6800

6800
1

6799

17262

17262

17262

17262

1

1

1

2

2

2
1

1

26719

700
26719

22

22

1731

1731

24266
241

19142

4883

133961
8619

18666

18666

11001
56

6438
22

1

6

19

1

6389

4507

4507

17785

17785

17785

2574

2574
8

2543

23

711
67845

2041

2041

3348

3348

5854
61745

4318

11094

10333

945

586

26558

2057

7471

7471

7471

457
26270

4

1

3

13534
24

1185

1
1185

56

54

54

2

2

1128

1128

1128

12325

12325

386
12325

1242
5723

1130

1130

13

13

1116

1116

1281

1281
1104

32

12

2

20

8

4

21

13

4

6

4

9

24

24

9

9

941
2

6
939

933

1431

1165
1431

14

2

51

51

15
1

14

10
11

1

173

4785
456

1048
3338

371

432

245

317

210

359

2

352

2

991

991

215
12275

8275
11

5

1
5

4

350
8258

1296
7699

2106
314

70
34

1

2

8

25

1588
71

7

1510

31
47

12

4

87

11

11

84

507
144

168

195

977
546

36

59

8

42

164
253

15

3

2

1

7

10

8

11

5

2

4

2

6

8

4

1

12

21

2718
179

218

247

221
210

11

352
1158

244

562

60

60

122
1

121

115

206
398

11

7

5

167

2

208

208

208

1

1

1

1

1

1

324
3688

1529

1527
440

444
429

15

333

333

138

2

2

170

2

2

1835

1835
394

243

2

5

337

337

210

9

4
457

453

175

2

1

3
97

72

72

17

55

22

22

687
144370

67684

67684

67682

67681

67681
55062

7314

2992

2313

1

1

1

1

2

2

2
1

1

1

56268
1

31

28

28

28

3

3

3

1

1

20381
56215

4326

4326

9

9

6851

6851

2361
11412

2522

2

2

6527

8403
13

22

8333

35

198

198

4635

4635

2

2

2

2

18

18

13

9

3

6

4

5

5

9874

304

304

304

304

304

9570

9570

1598
9570

6

4

2

6048

6048

1918

1918

399

399

9458
3

1

1

1

1

1

9443
21

9217

9217

9217

9217

205

2

1

1

1

1

1

1

1

203

203

203

11

11

11

11

621
18203

246

246
152

76

18

701

701

245

245

245
57

14

7

7

118

56

16390

16390

16390

16390

4

1

1

3

3

112221
571439

46874
1

46824

46824
3

46800

21

49

49

55

53

2

1

1

274719
56830

121242
44712

10395

10395

763

763

47271

27583
47271

19688

18101

18101

221

105
221

7

1
106

105

3

3

96426
17

12

12

12

3

3

45642
96394

10098

21144

99

8151

11260

11254

5

1

165

165

165

165

137404
3160

16698

16698
22

29

17

12

16589

58

117546

61678
117546

8051

30933

7709

9175

31

31

11

11

20

20

1

1

1

1

192538
251

11

11

11

11

172185
781

137471

11
137471

8

8

3

3

3

5

5

5

5

706
137447

136488
3

12
136480

136442

23

3

3

5

5

4

4

249
17

26

4

22

17

2

2

6

1

5

9
4

2

3

4
189

16

16

7

2

5

1

1

10

10

7

4

3

144
10

2

4

4

93
112

19

4

1

1

1

5

5

5

5

5

5

148
33891

2

2

33275
2164

9

2

2

7

7

5727

2
5727

2

5723

4040

4040

4040

1

1

4039

21335

21335
2627

1

1

90
2028

15
1047

1015

17

23
891

13

850

5

10313
1915

875
592

272
136

98
2

96

13

25
1

24

11

3

1

2

658

657
531

126

1

938

6
938

932

3
2294

2

1776
2289

70

206

237

1318

1

1317
5

1312

162
2312

761

590

1

2

796

937
5

932

515

417

22

22

2

2

22

22

579
5383

208

175

19

3

11

11

5

5

3

3

1754
436

748

570

579
2834

494
597

78

32
78

46

24
3

17

4

1

1

693

693

1

1

369
962

107

107

204
1

201

201

2

160
282

16

1

101
105

4

2

2

466

466

2

2

464

464

460

4

42

9

9

9

3
33

2

2

2
19

3

2

1

1

1

11

9

9

19493

8

8

8

19485

19485
556

19
1

5

5

6

6

7

7

10764

3

3

3

10761
2650

3456

1

1

15

1

4638

5

5

5

8140

8140

1

1

1

27
598

61
282

35

35

75

75

40

35

28
90

42

42
41

1

20

20

21

21

25

25

25

264

75

75

75

189

189

189

5808
52686

2424
34

51

51

51

51

38

38

38

38

2301
2

2271

2271

2271

28

28

28

682
13321

15

15

15

15

15

11804
1054

26

12

12

12

14

14

14

12
5776

13

13

13

15

15

15

10

1

1

8

8

8

1

1

24

24

24

21

3

1
25

21

21

2

2

1

1

11

11

11

2

2

2

6

6

6

12
26

3

3

2
10

8

1

1

2

2

2

5630
11

6

6

5613

5613

29

29

29

29

15

15

15

15

3

3

3

3

23

23

23

23

11

11

11

1051
170

30
3

3

3

6

6

4

4

1

1

4

4

3

3

6

6

133
489

12

12

177
15

33
9

1

10

12

1

3
64

1

18

3

11

11

9

19

2

2

7

6

1

2

2

54

54

27
2

18

3

4

2

2

25

25

25

25

5

5

5

7

7

8

8

34
5

7

7

7

6

5

1

2

2

12

12

2

2

6

6

1

1

16
2

8

8

6

6

12

6

6

6

6

6

6

18

2

2

2

2

10

10

4

4

95
14

3

17

17

5

5

5

5

3

3

8

8

3

3

4

4

5

5

9

9

6
19

6

7

40

23

23

2

2

15

15

20

16

16

4

4

195
27

1

1

10

10

8
20

6

6

21

19

2

4

4

3

3

7

7

4

4

10

10

42

28

28

14

7

7

8

8

16

16

16

11

11

11

4

4

11

11

11

1

1

1

1

19

19
4

15

15

86
6

26

15
1

14

11

49

33
49

3

7

5

1

1

5

2
5

3

3711

54
3705

1

1

1

1

28
7

5

6

6

10

44

44

3
38

7

3

5

11

6

3

5

3

2

3534
16

219

2

3297

6

6

6

90

90

18
90

3
50

29

18

22

22

575
35

191

191

191

191

28

28

28

28

23

23

23

23

222
19

11

11

11

164
48

13

13

2

4

4

19

19

16

16

12

12

9

9

3

3

12

12

9

9

17

17

5

5

5

5

5
23

6

6

12

12

22

22

22
18

4

54
4

17
47

1

1

14

14

7

2

5

8

8

3

3

3

3

19
155

69

9

9

9

60

36

36

24
4

10

10

10

15

15

8

8

8

7

7

7

19

19

19

19

33

33

2
33

20

20

11

11

11

21078
487

7412

7412
559

890
28

447

415

1627

1627

42

42

1

1

39
4293

35

1

4218

7826

7826
1527

31

31

2
3650

3648

96

6

90

1064

1064

1458

1458
43

1415

5353

5353
131

10
5060

9

5041

16

1

15

16
79

13

6

2

42

49

3

46

18

18

7907

7858

7858
6

50

50

7802

7802

48

48

48

48

1

1

1

1

2148

2148

2148

2077

2077

71

71

4

1

3

4126219
169524

830252
53766

145477

8648
411

3

3

100
4095

1062
38

1

1

70
726

239
6

233

208
205

3

1
202

201

7

297

297

297

774

774
266

63

63

86
83

3

296
2

294

2
63

61

1849
50

158

158

169

169

1472
20

1264

1264

188

310

310

2
310

308

1331
8

112
335

112

112

112

111

111

985

985
255

705

25

1

1

1

2

2

2

2255

415

415

539
203

102

14

79
88

5

4

221

13
10

1

2

123

123

123

123

234

234

234

234

628

628

628

628

300

300

16

16

553

553
89

165

165

299

299
181

61

57

58277

58277

3011
36970

634
5707

3250

2371
3250

288

288

591

591

1823

1823

1823

3101

3101

269
3101

955

1877

12

12

12

12

1259

1259

1259

1259

5408

5408
764

2771

2771

1873

1

1872

8925

8925

8925

9547

31

31

31

31

1

1

1

9515

9515
1559

6963
1183

5611

169

993

8086

8086
4

1

1

1

8081

8081

8081

8081

4
13221

13217

13217
4520

56

56

56

2961
28

2

8

2920

3

20

20

2096
7

3

2086

3520
386

846

2288

2119

169

11

11

33

23

10

10

2414

2414

574

574

1840

1840
4

1836

16495
260

5460

5460

100
5460

4558
1

4073

4073

484

484

802

802

802

6612
113

2

1

1

1

1

1

1

1

1

904

904

904

904

904

3982

821
3982

89

89

89

76
3072

2472
315

242

725

333

505

352

524

524

1611

1611

1611
148

827

827

630
1

627

627

2

6

6

24
4163

3050

3050

3050

3050

3050

3050

1089

1089
2

1083

1083

2

2

1081

1081

1
4

1

1

2

1

1

5653

5653

5653

330
5653

5

5

5

3489

3489

3489
3

3486

3
1829

20

20

1806

9
1806

1797

8

8

8

8

8

8

8

1331
53982

5435

5435

5435

5435
801

6

6

1016

1016

250

250
12

238

3362

3362

3362

893
23121

5990

5990
158

4277
551

2622

2622

4
1104

1100

1100

1555

1555

1555

2

2

2

2

1

1

16236

2697
16236

1002

1002

4
1002

998

5
1532

5

5

670
1522

274

578

578

699
7360

606

606

606

606

2218

2218
765

606

606

134
161

19

8

619

66

1

3835
331

2817
387

1

2125

132

132

172
1

171

686

686

1

1

2

2

2

781

781

781

128
2864

912

912

315
1824

622

2

885

24095

2963
24095

16767

16766
1288

2253
13536

1353

344

344

8167
9586

568

851

2

2

1940

1939

1

1

1

1

1

236
4365

1837

1837

1837

2292

2292

2292

50470
630285

424118
2699

9519
416711

31770

31770

4260
31770

1880
34

1

1

977
8

3

966
965

1

413

413

455

455

19
1330

806
255

150

26

108

45

222

222

505

505

369

369

369

369

1015

1

1

1

1

1

347

347

347

321

321

324

1

323

323

323

21

15

4

2

4507
431

1562
50

496
1297

175

175

160

17

1

83
1

82

85

85

1

1

59
2

57

70
56

1

13

4

145
1

144

215

215

215

227

227

227

3

3

196

196

196

157
376

82

7
134

127

1

2

292

292

292

236

229
1

228

7

194

193

193

1

2

1

1

248

248

248

92
374

140

140

142

142

193
366

74

74

99

99

1630
8107

647
50

225

225

370

2

125

10
125

115

51

51

186

185

185

1

6
378

159

39

39

174

174

1

1

494
274

116
118

2

18

13

71

55
636

203

203

171

15

4

188

188

466
1649

209

187

4

246

134

134

100

100

58

58

133

55

57

57

57

354
29

170

170

155

155

591
157

91

91

6
195

189

148
128

20

218

218

347
1

160

160

186

186

220

220

220

580
346

3

13

13

19

19

111
141

3

14

3

10

4

32

32

15

7

670

670

670

670

148
2378

841
91

132
274

142

233

243

243

120
809

183

228

250
278

28

292
580

1

240

1

239

239

25

1

21

21

2179
103

1180
29

200
279

79

275
872

30

274

104

171

18

896
117

201

201

149

427
241

186

186

1

1

1673
68

620
31

1
277

276

13

299

1
12

11

11

289
392

47

54
56

1

1

57
581

251
1

250

273

273

3125
145

1361

1355

1

5

3

3

526

526

526

545

545

6

6

539

539

277

277

277

277

375422
6406

3

3

3

3005

3005

3005
767

321

80

241

241

596

596

596

11

10

1

1

861

796
861

65

1

1

448
174

254

20

67222
351606

1843

1843
19

892

892

892

892

892

5

2

1

1

3

3

1

1

2

2

927

14

14

14

14
1

2

7

4

913

913
2

5

1

1

1

1

3

844

841
568

137

136

1

1

2

2

61

2
61

4

3

52

52

1

1

1

284

284

284

284

284

284

76311

76296
3736

13697

552

552

552

552

563

563

563

563

2

2

650

650

650

650

711

711

711

711

4942

4942

4942

4942

120
3721

2819
509

573

573

1

1

612

612

612

814
83

266

465

310

310

310

56

56

56

56

726

726

726

726

308

308

536

536

536

620

620

620

620

620

760

760

760

760

332

332

18792
472

2320

2

2

2

2318

2318
562

4
218

214

297

297

923
966

43

275

275

835

835

2

1

1

1

833
464

151

151

218

1347

1347
166

302

302

879
34

405

440

13216
665

543
7372

7

7

1429

1429

1429

579

579

593

593

593

366

366

366

972
379

167

260
426

34

32

62

38

1465
650

183

171

227

234

234

441
1418

267

302

408

408

511

511

511

511

4668
208

534

534

534

534

494

494

643

643

409

409

409

949
176

393

380

86
1431

331

982
1014

32

602

602

602

602

602

385
40071

1431

376
1431

446

446

609

609

806

806

806

806

806

35594
3336

383

383

383

383

728

728

728

934
17

423

423

494

494

494

717

717

717

717

1263

643

643

620

620

620

330

330

330

679

679

679

679

34
1309

607

607

668

668

1

1

1

1

18012
387

504

504

1

1

1

4
503

499

1

1

1

1

334
7628

5

5

1191
140

3

3

1

1

3

3

4

354
1018

45

53
482

291

138

105

32

22

22

5

3

2

313

313

219

219

379
950

1
67

66

113

113

4

1
158

157

126
105

14

7

103

103

4315
169

132
203

40
58

18

13

190

190

3753
3719

34

296

296

296

676

676

676

676

1791
95

547

547

417

417

417

299

299

386

386

2
386

384

384

47

47

7025
179

1

303

303

5873

5873

1
356

355

313

313

313

689

689

689

689

950

950

950

950

660

660

660

660

238
5603

793

793

793

862

862

862

64
1114

463

463

463

587

587

620

620

620

620

51
1976

465

465

465

985

985

475

475

475

34
1855

2
1147

1145
217

519

1

4

404

674

674

674

15

15

15

15

15

4240
137835

1284
26353

14474
1239

672

672

207
672

441

24

1337

398
314

30

29

25

1

1

938

938

938

1
318

317

317

77

103

137

2352

2352

2352

946

946

839

839

567

512

512

512

589

239
589

90

98

162

96
2015

27
1164

1137

1137

755

755

3495
968

813
188

191

120

314

1
198

37

97

63

209
810

30

29

55

2

20

43

15

57

43

149
47

28

63

11

59

99

16
706

12
312

300

378

378

2
1945

31
770

414

414

414

325

1173
676

347

150

1744

1744
203

131

131

609
141

168

300

273

273
185

69
49

20

19

19

528
10

334

184

8851

23
1053

76

2

74

954

6435

820
6435

1726

55

52

147

57

104

71

130

130

50

78

48

159

87

196

46

49

175

92

211
3863

3652

26

1363
16

1079

1079

1079

268

268

268

5810
107242

70179
1620

201
8003

6031

6031
1689

471

260

4
210

206

1

281

692

261

153
317

164

114

148

29

30

82

82

7

712

349

349

243
98

145

54

66

527

124

175

61

76

76

91

91

506

506

341

142

199

197

467

467

29
1771

580
1311

160
72

56

32

162

162

32

377

431

431

431

1588
5

1

1

895
105

420

420

370

370

687
51

251

1

250

385
187

1
140

139

58

2659
58968

4390
14

4068
1491

30

227

227

328

280

1

279

279

139

567
477

90

181

181

85
59

8

18

87

373
103

270

14

3

11

294

294

2

2

482

482

19

18

1

1

8
462

454

772

1

1

771
406

105

105

260
124

57

79

545
23560

1

1

1829
110

352

352

11

557

557

424
359

65

375

285
20666

5

20298
233

20022

43

78

78

519

519
221

298

14923
106

607

607

2839
14203

1

1

2

167
512

130

36

1

178

1

3

3

3

3

286
1984

1678
910

53

67

28
55

27

125

125

42

226

16

35

2
30

28

47

47

27

27

26

26

19

19

14
20

6

514
3790

151

104

317

86

2140
2194

54

228
300

72

124
118

6

727
1927

186

165

76

107
3

96

4

4

159

312
358

46

3

46

100

1

32

32

32

1074
3108

1036

333

646

57

18

919

30

31

7

7

7

265
4714

591
1613

1
158

157

218
182

36

96
108

12

26
126

70

30

39
35

4

66

66

70

70

237

237

2

2

17

1

16

735

735

352

352

352

413

413

316
2

305
5

300

6

1

2

985

985
557

126

302

2

2

14
5

1
2

1

4
3

1

1

1

1

1227
1

871
2

2

867

355

355

6239
206

435

435

500

4
498

494

1

1

1

2398
449

282

146

136

331

6
231

225

623

288

194

194

1690

1690

128
1010

302

302

4

308

265

3

1
14171

545
14170

155
1180

466

1

462

82

59

321

3

105
539

231

231

203

20

20

1660
63

99
1154

309

1

415

91

239

435

435

6

6

2

2

2

2

2

2

2

453

453

453

7941

1

1

6803
384

185

6234

1946

188

115

3438

176

173

198

1137

1137

1173

3

3

5
1170

1153
3

1150

12

533

533

533

533

683

683

683

17082
497

1

1

1

5369
200

202
2144

506
209

144
147

3

22

67
126

14

45

1

1

1436

8

1
1428

1427

2580
129

426

354
426

72

1

1

3

3

320

320

320

3

3

3

3
1

2

6
993

327
4

323

323

2

1

657
1

656

702
496

69

137
12

125

445

445

445

2194

2194

2194
199

637

637

505

853

853

365

365

365

365

365

394
3653

546

546

546

153
1625

292

292

223
1179

249

463
364

99

244

1

1

25

1
25

6

9

9

9

53

53

1
12

11

1

1

40

40

7

4

4

3

3

1003

205
1003

513

285

62
2884

1395

531
1395

411
339

15

30

27

217

236

1427
219

4

4

469

469

469

735

735

735

8

8

8

8

2111

2111
116

392

392

392

3

3

1189
414

71

71

150
45

9

96

554
1

553

411

404

404

1

1
6

5

68111
1787

112
15697

365

365

365

365

14635
492

757

757

757

757

4

4

4

1

1

1

2501
12681

660
1577

76

403
366

37

314

94

30

30

768
34

449

449

285

285

1149
3

308

838

1588
423

213
174

39

89

89

137

137

466

260

260

612

612

612

4486
557

540

769

769

502

319
3

316

430

516

853

700

204

172

139

185

585

1
585

2
583

581

581

1

1

1149
50627

42740
2184

629

627

368
627

259

2

2

2

1330

1330

1330

2143
35375

1

1

1972
6038

1

165

95

74

202

1

185

2

303

1

417

2

1

99
106

3

4

99
223

124

1

150

1

93

139

139

208

134

276

276

86

73

73

13

54

152

89

109

335

57

2

1

54

404

1

1

547

547

547

2
274

2

269

1

8421
25922

2638
99

367

540

540

317

171

38
66

20

8

126
275

123

12

5

9

86

86

504
620

1

115

69
97

21

7

206

2375

2

3

2370

423

423

93

93

190

10

137

137

54

31

31

269

269

53
117

6

58

444

67

70

343

343

128

142

49

49

278

44

385
2255

60
19

41

745

323
12

311

425

425

317

317

187

512

115

115

119

98

98

39

39

155

52
12

40

3

256

256

399

747
15

199
254

22

14

19

76

76

402

402

121

121

50

36

36

70

37

37

86

52
45

7

282

282

71

174

174

178

178

103

103

141

221

467

467

73

343

343

53

35

35

526

526

526

484

1

483

322

322

82

82

5

57

57

180

180

180

69

69

51

87

449

441

8

23
3222

91
2788

1344
115

205
251

35

11

69

527

527

382

2

2

221
938

3

477

237

413

413

410

1
410

409

1

502
6738

226

222

222

222

4

4

554
2907

328

328

328

237
638

113

143

14

14

60

71
7

29

15

20

371
11

206
164

42

154

154

501

241
501

58

113

22

67

117

117

117

398

398

398

1241

275
859

69
461

62

35

23

272

123
64

25

34

1

1

381
196

91

91

75
94

9

10

361

2
361

353

353

5

1

320
1501

546
321

4
42

38

9

9

28

28

59

59

7

15

15

16

16

49
16

1

32

209

209

209

218

218

206

206

206

2

2

14402

14402
279

622

621

621

621

1

1

1

1273

1273

1273
7

1266

730

730
1

33

33

696

696

9368
302

8603
204

134

134

935

316

5

7008

1

463

463

463

1506
77

711

711

718
2

716
15

701

624

624

624

60
4703

1006

1006
66

578

578

578

362

362

362

362

577

577

577

217
577

70
6

2

62

4

2

2

43

43

138
243

35

70

1722
1

1721

1721

1721
575

195

195

170

170

362
20

342

419
3

416

951

951

951

951

387

387

387

387

387

387

5

5

5

155697
5706

884

884

884

884

253
884

227
205

22

215
196

19

189

16180
184

649

649

649

649

649

649

9146

9146
138

628

628

623

623

1

1

4

2
8328

1105
8324

991

991

991

645
2280

1635
1

1634

925

925
802

123

1

1038

1984

1984

2

4

4

4

48

48

48

6201
416

766

766

17
766

742

7

3565

3565
140

342

342

3083
659

340

340

524

6

375

958
940

16

2

221

135

86

2

2

2

2

1452

1452
1

1451
778

210

3

298

17

17

145

17683
757

4419

4419
277

350

350

350

3792
873

424
188

53

53

183
143

40

104

104

104

224

224

224

768
215

55
3

52

235

13

6
187

181

63

157

157

157

81
503

166

99

120
157

21

16

739

1

716
738

22

1838

1838

1838

294
1838

268
228

40

242
3

239

6

367

309

2
349

347

3

10666
445

1116

1116

558

558

558

558

500

500

500

500

500

5702

5702

5702
2574

396

2149

530

53

1541
106

654

654

654

781

781

781

726

726

726
437

117

172

636

636

636

636

3

3

3

1
3

2

115244
3146

14

14

14

14

14

14933
826

803
8039

1237

1237

1237

38

38

37

1

777

777

777

537

537

537

4037
224

2198
567

396

158

158

198
144

9

45

242

242

366
397

12

19

240

240

719
1615

169
82

15
26

5

6

30
31

1

17

17

13

13

233

233
77

48
88

14

9

17

45
68

19

4

44

44

308
276

23

9

51
83

24

8

59

610

610

12

1

597
526

71

5512

5512
1478

962

962

766
962

90

106

1
1434

1433

1090

1090

548

548

14

14

542

542

542

542

94665
12634

912

3

1
3

1

1

1

1

1

909

906

906

3
906

903

3

3

3

2

2

36984

918

918

918
648

123

123

147

147

616

1

1

1

615

615

615

615

1664

1663

1663
141

844

844

678

678

1

1

24
2023

305

305

2

303

303

1694

1694

1694

1694

235
1624

454

454

454

935
40

497

497

497

398

398

398

5927
59

832

2
832

8

1

807

14

380
2716

1577

1577
1081

169

110

59

327

327

756

756

3

3

1240

1240

1240

1240

1080

1080

1080

1080

154
5879

1
818

817

817

817

1789

1789

1789

913

876

21
1994

1533
287

569

677

677

440
1

439

1104
37

2
536

532

532
4

528

2

531

531

1
20

3

3

9
1

5

1

1

1

7

7

130
3426

740

740

740

104
1778

2

2

1

1

793

1
793

792

372

372

505

505

1

1

778

778

778

14907
1153

553

4
553

549

1006
46

452

452

508

508

1546
134

420

420

420

49
992

463

480

480

339

4

4

335

335

6
934

473

473
3

470

455

12
455

443

580
6070

715

715
455

165

95

10

10

1112
383

325

325

2

402

2050
503

391

391

322

452

452

382

840
83

6

2

6

597

597

146

763

763
725

38

569

568

543

25

1

1

561

561

561

51
1101

33

33
10

23

493

493

493

524

524

524

478

478

7
478

471

597

597

270
597

116

211

44133
1538

156

156

156

156

156

744

744

744

744

744

6282
365

1185
7

491

491

491

491
337

74

80

687

687

687

687

1369

47
1369

1016
138

326

326

552

552

306

306

306

1924
82

1166

1166
143

550

550

473

473

676

676

676

676

1

1

1

1

1

1438
129

675

675

675

675

634

634
251

2

207
1

206

174

2402

2402
25

858

858

858

1519

1519

1519

2412
33011

702

2
702

582
692

110

6

1

1

1040

20
1040

2

1
372

371

646

646

164
1479

513

513

513

802

802

1103

115
1103

249

249

674
739

65

1639

1
13

5

2

1

1

1

1

1

334
1624

522

522

11

757
5

752

2

2

3275
131

1052

2

1050

1360
52

671

1

636
2

634

732

732

3073
24

79
2

77

2

2

2072
903

493

387

289

896

896

896

1

1

1

6178

6178
156

5244

1

777

64
2200

1

1

837

837

837

573

571

2

725

725

725

1350
8

771

771

571

571

571

387

387

387

387

1347
23

2
500

498

7

491

1

1

3

3

820

820

2499

679

679

679

1357

1357

1357

1

1

462

462

15

15

15

1114
43

2

1

1

1
538

536

536

1

1

1

530

346
529

88

88

95

95

1

89
2444

2

1

1

410

410

1943
31

794

794

1118

1118

751

3

3

747

747

1

1

1

1

1

1

1

1

1

2486

2486

331
2486

245
1568

596

596

727

727

587

587

30

30

694

141

552

1

416450
2092

19664

2567
19664

10
6808

6727

71

17

17

10271

10266

2

3

1

1

394694
61

26
42

2

2

7

7

7

7

17
394591

9

9

20

20

394545

394545

2697361
23189

2644021
137281

1833
147532

13730

6
2

1

1

1

1

3

3

2

2

1

1

326
13724

6472

6441

6439

20
6439

3

4

6412

1

1

1

1

1

1

23

17

2

2

15
2

1

8

4

6

6

6

8

1

1

1

3

3

3

4

4

4

6925
2

2

2

2

6921

6921

6921

6921

1

1

1

1

1

7

7

4

2

2

2

2

2

3

3

3

3

5587
129201

1

1

1

1

238
16922

5

5

5

5

16648
1682

10
4

1

2

2

3

12024
21

12003

12003

12003

2930

2928

2

2

2925

2925

1

2

2

2

2

2

14
31

2
7

4

4

1

1

1

10

8

8

2

2

2

5160
106691

2332
62552

5

5

5

5

5

2503

2503

2503

2503

1460
57712

1
5807

5806
2561

1

1956

1288

1235
50445

46010

46010

3200

3200

3
4

1

1

972
38931

5

2

2

2

2

2

1

1

1

1

1

1

1

1

1

8213

8213

8213

2
2881

4
2874

1

2869

4

4

1

1

26859

26859

25

25

26834

4
2

1

1

1

1

1

1

1
40

15

7

7

8

1

7

24

24

24

2640

2640

3

3

3

2

1

2637

1

1

1

1

2636

42
2636

208

208

171

171

2215

2215

2

2

2

2

2

119
1

107

11

11

11

2

2

2

94

94
5

2

1

1

87

11
2

1

1

1

1

1

1

1

5

5

5

1

1

1

1

1

1

2352812
156484

81

81

20

20

19

19

1

1

61

13

13

13

24

24

24

23

23

23

1

1

1

8257
497550

3892

3886

3883

3883

3883

3

3

3

6

4

4

4

4

2

2

2

2

460322
4330

12757

12757

12757

12757

12757
3417

4

3378
3367

11

2

5956

58
13012

8369

8369

8369

8369

8369

10
4585

4509
246

1397
1

1392

1390

2

4

4

4

2865

2865
356

986

1523

1

1

1

3

3

1

1

2

2

1
63

1
9

1

1

1

1

3

3

3

3

17

17

17

36

5

5

1

1

4
30

4

21

1

430223

2408
430223

424889
8650

246
9884

201
7882

901
4

882

2
882

1

1

879

879

2

2

2

1

1

1

1

12
1

11

6

6

6

5

3

3

2

2

2

6780

1

1

1

1

2
6779

291
6776

4
1101

1

1

1

1

1

1

1095

1095
330

374

391

5384
196

373

373

373

1

1

1

4814

4814

4814

1

1

1

1

1

1756

1756

1756

1756

1756

406355

406355
22699

923
165048

163103
897

2158

2158

1
2158

2156

2156
3

2153

16
2153

2137

1

1

1

1

1

160048
882

2736
158053

2990

2990

2990
105

809

2

2

1

1

807

807

807

1

1

1

1

2075

2

2

2

2

2070

5
2070

2065

2

2

2

1

1

1

1791
152327

147523

389
23699

2163

46
2163

2
1025

1022

1020

1020

1020

2

2

2

1

1

1

1

1092

1092

1092

1092

21147
273

857

857

857

857

857

857

680
20017

9305
496

2915
1

708
2914

495

1
495

494

2

2

2

408

408

408

1

1

1

3

2
3

1

1

474

474

474

474

2

2

2

820
153

464

464

203

201

2

1

4193

3

3

3

3

3

3

4190
262

2610
465

1888

1888

257

257

1

1

2

2

2

1

1

1

1

1314

1313

2

2

1311

1311

1

1

1

1701

1701

1701

1701

1701

10032

10032
327

822

6

6

6

6

816

1
816

815

1

1

1

955
8882

7

7

7

7

2

1

1

1

1

1

624

622
9

602

602

602

11

11

2

2

2

2198

40
2198

953

953

1205

1205

5096
607

480
3624

595
2274

655

474

550

463

463

407
2

1

404

596

596

3

3

593

593

269

269

269

2223
123824

24

5
20

1

1

1

14

14

14

14

4

2

1

1

1

1

1

2

2

2

74649

74649
3

74643
2

3

3

3
2

1

1

74638
664

72699
1551

69933
10724

4

4

3

3

3

3

1

846
21485

1603

3

3

3

233
1316

2

2

327

327

1

1

753

753

2

2

2

2

2

1

1

13
280

13

13

2
254

252

1

1

1

367

367

5

5

5

362

362

362

1

1

1

1

3

3

3

3

1

1

1

1

2

2

2

2

1

1

17

17

17

16

1

1

397
10819

1

1

1

1

399

398

398

1
398

397

1

1

1

1

121
5330

1129

1

1

1

133
1128

578

578

417

417

319
1

317

317

317

1

1

1

1

3757
46

403

403

403

403

3307

3307

3307

1

1

1

4

4

4

4

287

287

287

287

287
5

1

268

13

1480

300
1480

683

102
683

477

477

104

104

134

134

134

363

363
51

131

181

529
2925

283

282

282

1

1

152

152

152

1
132

127

127

4

4

1829
32

188

188

233

233

1376

1376

1288

38
1288

858

858

392

392

5452
40

513

513

513

513

4899

4899

159
4899

1

1

1889

1887

2

2850
580

989

1103

178

1084

1084
232

1

1

730

730

121

121

1100
22922

3915

3915

3915
233

706

705

705
150

321

321

234

234

1

1

1

1

2976

2976
823

285

285

285

1002

175
1002

614

213

866
221

157

157

157

128

128

237

237

123

123

6

6

6

2

1

1

1

1

2

2

1

1

2192

355

355

355

326
1837

367

367

484

484

153
660

202

305

2017

2017
6

1337

1336

1336

1336

1336

1

1

1

1

674

674

674

674

674

288
13692

2

2

2

2

2

3517

3516

3516

3516

629
3516

626

2054

207

1

1

1

1

1

254
9885

3630
97

1702
223

455
1301

374

95

73

144

79

81

177

177

1

225
1831

593

593

613

613

400

400

6001
456

866

866

866

3899

747
3899

1

587

587

788
211

200

318

59

1252
342

144

317

240

209

524

524

780

780

780

780
399

141

240

68
3066

1687
36

404

404

404

400

1

3

42
1247

600

600

2

1
598

597

605

605

605

15
1311

626

626

626

626

670

670

670

670

2483

2483

2

2

2

2

2481

290
2481

561

561

561

1630

1630
2

1628

2985
13

1187

1187

1187

1187
1

1186

1186

1785

14

14

11

2

2

1
9

8

3

3

3

1771

1
1771

1

1

1

4

1

1

2

2

1

1

407
1764

430

430

927

927

1

1

1

5473
146

3326

3326
196

342

342
233

45

64

196

196

196

1212

1212

1212

1

1

1

1

1

1

1

1377

1376

1376

1376

1

1

1

574

574

574

574

1427
39

649

649

649

739

2

2

4

4

733
265

178

290

290

791

791

791

790

790

790

1

1

1

1214

1214

1214

1214

1214

1214

1

1

1

1

1

1275

1275

4

4

1271

1271

1270

1269

1269

1269

1

1

1

1

1

1

1

1

3

3

2

2

2

2

2

1

1

1

1

46928
398

44829

666
44829

1604
43048

503
39

192

272

8729

8729

79
2511

1911

521

29701
400

257

1095

25616

289

2044

1115

1115

1115

1115

1701

1701

1701

1701

3013

3013

70
3013

1

1

1

1301

1301

1301

1641

1641

1641

1113

1112

1112

2
1112

1110

1

1

1

1

1022

1022

1022

1022

1022

646
218608

216729
10

11

11

11

1

1

1

1

2
10

1

1

1

7

7

3

2

2

216708
2621

8034
127

2
5574

38

37

2

2

2

10

1

1

9

2

2

1

1

6
2

1

3

25

3
25

1

1

9

9

1

9

8

1

2

2

1

1

1

1

181
5534

1

1

1

1

1

1

12

2

10
1

9

9

3726

3726
5

9

2

2

7

7

3712

3712
619

2012
4

2008

2
1081

1079

1612
5

2

1

1

1

1

1

1

1

1571
94

801

2
801

799

799

3
673

1

1

1
664

1

662

1

1

1

1

1

1

2

2

2

2

2

2

1

1

2
23

1

1

3

3

3

1

1

1

1
12

11

1

1

10
4

2

1

3

1

1

1

1

1

1

1

1

2

2

1

1

1

1

1

1

11
1

1

1

1

4

2

2

2

3

3

3

2

1

1

1

1

587
2333

421

421

421

460

460

460

865

865

865

206053
5779

91106

91106
1776

903
78721

63303
327

2220
460

728
64

43

43

43

43

621

621
1

620

1032

2

1

1

1

1030

216
1030

559

255

60756

60756

60756

60756

2122
60756

56416
7722

1619

1619
121

31

31

318

318

318

1042

1042

22

22

85

85

140
10035

3136
73

82

82

82

301
2911

14
472

131

131

327

327

327

102

102

102

2036
39

1714

1714
897

817

37

37

246

246

70

70

70

6759
404

85
395

104

104

153

153

53

53

408

408

408

80

80

80

5472
246

4136
54

3060

3060

1022

1022

336

336

336

2

2

752
5

605

2

603

142

142

615
15

80

80

80

48

48

48

139

139

139

45

45

45

113

113

113

85

85

85

90

90

90

2238
162

20

20

20

18
1144

289

289

33

33

804

804

804

41

41

41

131

131

131

6
92

52

52

34

34

37

37

37

2
48

20

20

2

2

24

24

33

33

33

298
1

7

7

21

21

269

269

24

24

24

16

16

16

8
74

20

20

13

2

11

18

18

14

14

1

1

35

35

35

83

36

36

47

47

496

18
496

211

70

141

267

267

94

94
6

34

34

54

54

168

168
11

124

124

33

33

27

27

27

27

27

1183
15

1097

1097
77

871

871

871

1

1

13

13

20

20

9
115

58

48

25

25

25

22

22

22

24

24

24

224

224

224

224

212

212

212

212

668
42

105

105

105

47

47

47

80

80

80

21
394

343

343

30

30

78
834

78

78

78

73

73

73

501

501

501

13
104

53

53

38

38

9
1585

6
823

99

99

99

68

68

68

439
3

316

316

29
120

55

55

36

36

83

83

83

128

128
7

38

83

753
9

452
45

378

378

29

29

292
10

197

197

197

85

85

85

34

34

34

34

3605
21352

133

87

87

46

46

416
8

346

346

45

45

17

17

1441
122

30

30

694

694

62

62

160

160

373

373

257

184

184

73

73

31

31

31

49

22

22

27

27

80

80

80

41

22

22

19

19

36
3

19

19

14

14

166

166

166

27

27

27

27

27

27

45

45

45

2402
68

31
1

30

30

7
84

27

27

50

50

39

39

39

2
244

101

101

40

40

19

19

37

37

12

12

33

33

19
90

26

26

29

29

16

16

41

41

41

82

82

82

1350

1350

42
1350

1308

67

67

67

16

16

16

290
8

13

13

58

58
6

25

27

138
4

24

24

45

45

20

20

28

28

17

17

73

49

49

24

24

421
1

382
21

23

338

37

37

1

1

72

72

72

429
33

21

21

171

171

31

31

28

28

45

45

48

48

14

14

38

38

1
625

33

33

591

591

48

48

48

24

24

24

42

42

42

29
103

35

35

39

39

8
78

42

42

28

28

13
111

28

28

28

25

25

25

45

45

45

1441
189

93

93

237

237

178

178

29

29

110

110

321

321

284

284

22
306

53

53

53

49

29

29

20

20

51

51

51

107

58

58

49

49

24

24

24

52
1845

35
1

10

10

24

24

16

16

16

16

16

16

1337

17

17

1276
111

713

92

92

360

25

25

19

19

23

1

22

22

76

19

19

40

40

17

17

29

29

29

15

15

15

37

37

37

17

17

17

56

56

56

56

19

19

19

3
117

17

17

26

26

11

11

23

23

13

13

24

24

30

30

30

20
409

182

182

24
2

22

43

43

140

140

3
118

71

71

44

44

2

2

2

6354
136

339

339

28
339

311

18

17

17

1

1

248
7

7

7

17

17

217

217

16

16

16

16

2
502

407
7

364

364

2

2

2
34

23

9

15

15

3
78

47
4

12

12

31

31

28

28

28

21

21

21

37

37

37

16

16

16

1346

68
14

40

14

1278

1278

42

12

12

30

30

19
3633

81
3614

669

669

669

2864

2864

94

94

94

44

44

44

195
9

122

3

3

119

119

64

64

64

180

7
180

67

67

106

106

8
479

117

117

117

117

354

354

354

42
287

122

122

122

123

123

123

3
388

22

22

22

21

21

21

30

30

30

275
52

2
181

134

45

42

42

37

37

37

10
1046

25

25

25

55

55

55

237
8

14

14

215

215

499
5

444

444

50

50

164

164

164

56

56

56

1602
136

242

242

242

38

38

38

33
1186

131

131

171
20

124

27

851

851

40
935

95

95

95

20

20

20

236

236

236

78

78

78

78

96
15

47

47

17

17

17

17

51

51

51

208
33

38

38

112

112

25

25

64

64

64

47

47

47

142

142
5

101

101

36

36

737

5
737

47

47

685
238

128

225

94

8
216

18

18

18

13

13

13

9
52

11

11

20

20

12

12

125

101

101

24

24

1103
4

526

526

526
28

498

573
34

1

462

462

29

29

47

47

2218
199

229
45

26

26

26

158

158

158

1186

1186
168

102

102

73

73

66
243

14

14

149

488

486

2

28
112

52

32

176

175

175

175

175

1

428

428

428
43

181

181

204

14515

14515

314
14515

885

885

885

885

647
13316

922
1

79
921

548

548

548

294

294

294

243
11747

811
80

80

80

80

172

2
172

170

479

479

479

56
823

558
23

377

158

209

209

209

9870

9870

9870

10609

10608

380
10608

8622
3

8606
287

143
6419

368
4699

3974
657

4
551

518
170

6
9

3

1
94

93

4

3

3

1

235
5

228

2

1

1

2

2

1

1

1

2

2

5
29

1

1

1
5

1

3

1

1

1
13

1

2

2

4

4

5

4

4

45
667

265
3

1

1

9
4

5

241

241

2

2

6

6

3

2

1

357

2

2

2

355
72

129

154

1
7

1

1

3

3

3

2

2

2092
91

79
568

1

1

107

107

1
378

377

3
2

1

1

9
1431

119
865

72

141

1

4
285

218

63

194

53

555

555

2

1

1

1

1

1

1

1

1

1

1

8

1
7

6

6

1

1

1

349

349

2
349

1

346

1568
30

1

1

1

628

623

618

618

618

2

1

1

1

2

2

2

1
5

1

1

2

2

1

1

908

908
2

905

1

1

1

1

1

9

7

5

5

5

5

2

2

2

2

2

2

2

1
2

1

1900

1899

2

1

1

1

1

1

1

1897

685
1897

888

3

885

168

168

156

156

1

1

1

13

1
11

1

1

4

1

1

1

2

2

1

1

1

2

2

1

1

1

2

1

1

1

1

1

1

1

1606
2

4

4

2

2

2

2

3
1

2
1

1

2

2

2

4

1

2

2

2

1

1

1591

1591
180

427

427

2

1

980

980

1

1

1

1

1

1

645
109168

1209
105878

620
3609

2

2

2

2

848
1

847
1

846

846

263
1217

399

399

399

3

2

2

1

1

295

295

295

256

256

256

1

1

1

922

922

922

922

922

101060
5300

5707
91036

49729
2546

34
2244

101
1069

494

494

474

474

530

530

530

530

611

611

611

611

87
2197

1376

293
1376

146

146

78
937

669

190

134
734

125

125

125

475

475

475

441
15433

1764

1764

1764

1764

2501

380
2501

165

165

1

1

1955
305

660

889

101

5628

485
5628

203
3545

72
962

321

321

569

569

2380
170

219

219

1769
315

66

584

317

487

222

222

222

81
1598

991

991
176

278

537

284

284

284

284

242

242

242

242

5099
4

1

1

1

1

410
5094

844
12

64

64

64

768

768
53

193

389

133

133

3840
406

713
33

104

104

576

576

39
232

74

74

74

119

119

739
274

82

82

49

49

183

183

99

99

52

52

1433

1433

1433

317

317

317

371
13505

9293
742

804
109

270

270

128

128

27

27

177

177

93

93

83

83

83

83

228
2073

24
317

98

98

195

195

195

6
970

934
113

564

564

257

30

30

558

558

558

133
3178

1756

1756

1289
158

179

179

607

345

112
399

67

67

175

175

45

45

2014
244

224

224

1546

1546
292

1178

76

3841
243

62
513

210

210

241

241

767

767

767

403
2318

135

135

135

65
1254

535

535

517
38

271

208

137

137

526

14
526

301

301

211

12557
165

384
10158

1

1

1

1

716

716

1
716

1

714

790

790

790

854

853

853

1

1

2907
267

1

1

1

307

1

1

306

306

1

1

1

77
2037

1

1

195

195

1764

1764

294

294

294

4501
520

885

885

264

1
264

263

259

259

295
2573

814

414

561

489

4

4

4

1

1

2234

2234

206
2234

2

2

793
141

256

396

1233

1233

1247

1247

1247
440

384

423

822
35600

95
18469

16319
421

12
1048

4

4

583

583

583

583

449

449
13

435

1

4535
363

730

730

730

609

609

609

2

2

2

2385
119

803

803

1463

1463

446

446

446

8707
73

426

426

426

426

426

8208
677

435

1

1

1

434

433

433

1

1

6

6

6

6

2944
204

1475
75

1

1

1179
1

1178

220

220

891
118

258

258

58
306

135

113

113

209

209

147
374

122

122

104

104

1

1

4146
48

372

372

372

387
3726

86

86

1

1

193

193

85

85

935
149

553

233

2038
91

7

7

1

102

102

1837
60

1

147

1627
1628

1

1

1

1

1608

1607

1607

1607
381

161

161

91
977

89

91

91

706

1

1

87

87

1

2055
55

479

479

479

1521

1521

1521

912

912

1
911

909

1

1

1

14967
177

13366
33

846
12854

244
2296

92
1653

340

340

340

340

83
484

216

3

213

185

185

737
1

2

2

734

734

734

2

2

1

1

1

1

397

1
397

396

1084
9712

238
3805

3053
367

409

1
409

1

407

2277
311

80
621

283

258

1174

1174

171

2
171

169

514
86

136

136

2
292

290

4823

521
4823

146
1180

158

3

155

169

169

506
49

299

158

201

201

201

3122
322

265

1

264

1437
167

344

691

235

194

194

252

252

439

439

4

2

2

209

209

479

479

479

479

1424
49

924
99

1

1

1

344

344

344

480

8
480

472

451

3

3

3

448

448

448

430

430

430

430

281
3000

432

432

432

432

364

364

364

364

364

1
421

420

420

420

431

2

429

429

429

350

350

350

1
350

349

721

721

721

721

57
1724

824

824

824

824

843

1

1

1

1

842

842

842

842

2645

682
2645

1220

1220

1220

743

743

743

1233

1233

1233

1233
5

1228

2926
168

2641

3
2641

2

2

2

2636
130

70

1

69

2421

2421

1

1

14

13

1

117

117

117

111

111
75

1

9

26

6

6

6

25079
743

688
20203

144
10112

5993
2

1

1

1

1

1

3
5990

1

1

1

1

1

5973
32

16

1

1

1

15

1
15

9

5

1

1

1

1

5924

36
5924

5

5

5

17
5881

2

2

2

2

5860
10

5850

2

2

2

13

1
13

9

9

9

1

1

1

2

2

2

3975
2

3971

3971

1
3965

3964

3964

6

6
1

2

1

1

1

2

2

1

1

1

1

9403

12

12

12

12

12

12
2

1

1

1

1

1

1

1

1

1

4

4

4

3

2

1

1

1

1

4
1

2

2

2

2

2

1

1

1

1

985
9391

4842

4842
765

1596

1596

1596

2481

2481
1

2

2478

2

2

2

2

1

1

3562

3562

4
3562

1

1

3556

2

3551

3

1

1

4133

4133

4133

1

1

1

1

4132

4132

4132

4132

4132

1698697
35648

1426079
28241

199069
941

10111

70
10111

665

665

665

665

665

2

2

2

1

1

1

1

1

1

1

1

1

1

363
9373

5508

830
5508

2648

830

1200

3502

3502
2135

80

56

96

104

279

81

101

349

45

176

55

47

74

3104
188017

1

1

1

1

1

157002
2643

51571
72

49836

49836

49836
87

15067

15067
2720

685

492

442

919

1666

231

7282

628

2

11
5

4

4

1

1

1

1

1

34671

34667

34667

1
4

2

1

1663

1663

1

1

1

1662

1662

1

1

1661

45186
714

1450

1450

1445
129

1

1

639

639

2

2

674

674

5

5

5

4

4

4

4

4

2912

1

1

1

1

2911
344

596

596

596

596

967

967

967

1
1004

170
1000

510

320

3

3

646

646

646

646

346
39460

461

461

461

461

2
38206

38204
1027

23869

23869

213
603

112

190

88

293

293

10713
85

339

10141

148

2

2

675

675

237

237

785

785

446

446

446

446

1

1

1

1

266
57602

96
46210

2386

2386
107

1324

1324

955

955

955

266
43728

30694

30694
252

29190

1252

11631
388

830
2096

301

397

311

257

9147

9147

1137

1137

1137

1137

25
11126

9920
187

7742

7742
3

18
1

1

16

7721
1

1

7719

1986

1986

1986
501

434

1051

5

4

3

3

1

1

1

1

1

1

1

1

1

1180

1180

1175
1

1174

5

1

4

3

3

3

3

3

3

1852
27907

3742
208

653

653

651
653

2

453

453

453

453

2044
103

312
1627

733

582

1

1

313

313

384

384

384

384

36
2120

385

385

385

385

8
910

214

214

214

1
688

687

687

789

789

789

19576

19576
83

3901

3901

3901

15592

436
15592

14606

302

248

617

614

614
160

203

250

1

2

2

2

1

1

1

1

1217
1193988

4672

4672

4672

2

2

2

2

416
4670

1983

1983

1983

2271

2271

2271

1188088
29927

50806
509

949
48285

6
11634

46
1

7

7

2

2

2

2

2

2

17
7

2

2

1

1

3
1

2

1

3

3

1

1

9
10

1

1

5

1
5

4

1

1

1

15

15

15
6

1

1

8

2

2

3
11565

3

3

3

3

21

5

2

2

2

2

1

1

1

1

1

1

1

15

14
1

7

2

5

5
6

1

1

1

1

1

1

3

3

3

11535
707

67
1

19

1

1

1

1

16

16

10

6

2

2

2

45
3

30

22

22

3
8

3

2

4

1

1

3

1

1

1

1

1

1

1

7

3
7

3

1

1
2

1

1

3009

1
3009

8

8

3000
564

381

2055

7752

10

10

10

10

7742
181

1

1

1

1

6025
2

1

1

6022

1

6021

2
6

3

3

1

1

6
1529

1506

1506

17
2

2

5

5

3

2

2

2

2

2

1

1

1

1

1

1

1

1

1

33224
155

147
25353

17778

737
17778

31
2334

73

73

12

2

10

2218
386

495

942

6

357

31

1

14690
1609

3

3

1

2

1237

1237
276

46

6

96

244

56

191

153

125

1

43

5366
641

354
3711

1247

1247

2110
198

132

1

1

395

1239

1239

145

1014

1013

1

125

1
125

1

122

1

6350

6350
1177

2268

1

2904

17

17

17

7428

7428

346
7428

258
4827

1225

1225

1225

3344

445
3344

317

2582

2255

2
2255

2242

2242

2242

11

2
8

6

3

3

56
7716

4539
1

4538
128

426

425

425

425

425

425

425

1

1

1

1

2670

247
2670

1

1

1

1

1

1

1

1184

1184

1
1184

1183

1238

1238

1238

1238

1314

1

1

1

1

1

1313

1309

1309
6

4

4

55
1299

1

1

1242

4

4

4

4

3121

1

1

1

1

1

1

3120
2

183
3110

1031
1

1023

1023

1023

1023

7

7

7

1896

388
1896

716

1
716

713

2

792

792

792

3

1

1

1

2

2

2

5

4

4

2

2

2

2

1

1

2473
31

2378

2378

2378
18

8
2

3

2

1

1

1

2

1

1

2

2

3

3

4
16

2

2

4

2

2

1

1

1

2310
8

2296

1

5

6

6

6

6

6

5

4

1

64

51

51

51

51

51

9

5

1

1

1

1

4

4

4

4

1
2

1

1

1

1

1

2

2

2

2

4

1

1

1

1

2

2

2

2

1

1

1

1

1

1

1

1

1

1993

1993

20
1993

7

7

1966

1966

19

19

3

3

3

1

1

1

4

3

3

1

1

11

11

3

8

8

1107355
2171

1102906
31004

549340
1665

2233
435370

51810

1

1

1

1

1

51809
745

109
30516

2826

2

1

1

1

1

1

1

4

4

2
4

2
1

1

1

1

1

1

1

1

1

1

1

2818

2

2

2

2

2816

2816

2816
541

442

1833

4

4

4

1

1

1

3

3

3

27577
157

3
12813

12802

12802

10

10

10

12792

12792
1

12791

4

4

2

2

2
1

1

2

1

1

1

1

1

1

1

4

1

1

1

1

3

3

3

3

2

2

1

14607

14607

14607

14607

14607

14607

14607

3
20545

3

3

3

3

621
20539

13408

13408
1

3

3

3

13404

1

1

13403
1737

17

1

1

7941

3706

18
6510

1
34

22

4

4

4

18

18
1

17

1

16

6

6

4

4

1

1

1

1

1

1

4

4

2

2

2

2

9
54

3

3

3

3

2

2

1

1

1

1

4

3

1

1

2

2

1

1

1

3

3

3

3

30

1
30

1

1

4

1

1

2

2
24

10

12

2

2

2

2

1

1

1

1

11

11

11

7

7

4

3

1

6382

6382

18

18

1

16

1

1

15

15

1

1

1

6364

6345
1

6344
384

3

3

4813

4813

7
1144

2

1127

8

12

1

1

1

11

1

10

10

1

6

6

6

6

11

11

11

11

11

3

3

3

1

1

1

1

2

2

2

2

381327
560

365582

365582
17517

306813

1904
306813

32

32

22

10

4

4

4

188
15

1

1

1
170

80

2

8

13

66

2

2

11

11

11

253

4
253

18

231

1

1

35

35

8

27

1

1

1

37

37

37

3
2

1

1

303728

42
303728

8

303678

1

1

1

615

615
1

552

32

30

41252
6536

840

19
7

1

1

10

10

1

1

821

821

821

23166

1

1

1

23164

23164
377

67

22707

13

1

1

10710

1

1

1

488
10709

9897

9889
9897

8

324
27

147

150

15185

12
15185

1434
15150

2512

2512

2512

2512

2512

11204
8

1
11196

5

1

2

2

2
11190

17

11171

23

2

2

2

21

21

21

112299
81

37

1
37

8

8

8

28

28
8

1

1

1

15

3

59
112181

9

9

4
7

3

3

2

1

1

6
112113

112107
68

112015
1792

804
108330

35

35

107414
66629

4

4

5

10
2

6

2

7

25646

15100

1

1

5

6

1

19

19

30

30

28

27

1

1818

1818

1817

1

75

75

5

70

6
3

1

1

1

2

2

2

18

18

18

2

16

2

2

2

2

2

1

1

1

1

1

3

3

1

1

1

1

1

1

1

2

2

2

2

2

2

2668
522562

1

1

1

1

1

2

2

2

2

2

2

2

6807

6803
399

4476

4476

4469

4469

4469

4

4

4

3

3

3

3

1928

1928

1928

1928
1

1927

2

2

2

2

2

2

2

1

1

1

1

1

1

1

1

1
513084

3

3

3
2

1

1

1

513079
28

8

2

2

2

6
1

5

5

513043
8410

69655

2
69650

69645

1

1

1

1

1

69644

69644

69644

69644

1

1

1

1

2

1
2

1

1

5

5

4

4

4

4

1

1

1

1

1

22985
434978

277
63845

864
1

856

2
849

843

843

842

842

1

3

1

1

2
1

1

1

1

3

1

1

1

1

1

1

1

1

1

1
4

3

3

3

7

5

5

1
5

3

3

1

1

1

1

1

1

2

2

1

1

1

1

1

1

1

1

3317

3316

3314

3314

3314
523

1
1512

1511

1279

1

1278

2

2

2

2

1

1

1

1

1

1

1

59387
1144

19526

1

1

1

1

19525

19525

2
19525

515
19523

225
16131

5353

5353
8

5

5

2

3

5330
432

23
338

272

43

571
20

496

5

42

7

1

82
3989

3888

19

1

1

1

9

9

9

9

67
10551

142
5334

4

4

2

2

2

2

2143

2143

2143

2143

2143

3042

3042
508

627

627

1907

5

1902

3

3

3

3

5150

5150

5150

2

2

2

2

9

9

9

9

9

2868
127

1356

3

3

3

1353

1353

1353

1353

7

7

1

1

6

6

1304

1301

1301
9

1

1

1

1

1290

1290

3

3

3

74

1

1

1

1

1

1

1

72

72

70

2

27052
370

4
23010

130
23005

1

1

1

1

1

1

1

1

1

22873
1135

17270
1121

161
11914

136

136

11396

11396

221

221

249
4235

283

283

356

356

7
3229

158
3214

487

1871

698

8

8

118

118

798

798

798

4

4

4

3666

3666

3666

1

1

1

1

1205
3

1

1

1

1192

1192

1192

1

1

1

6

5

5

1

1

1

1

1

1

1

1

1

418

1

1

1

410

398

398

7

7

5

5

5
6

1

1

1

1

2049

2049

2049
1

1

1

1
2047

2046

11665
84

6464

11
6464

484
6452

5070
3

5062
3

5059

5

5

898

898

898

1

1

1

1

5117
71

3406

1

1

1

3405

1

1

3392
909

350
1551

1
648

647

553

553

932

932

932

3

3

3
1

2

2
9

5

4

4

1

1

2

2

1630

1630

1630

1630

10

10
1

1

1

8

4

4

4

4

4

4663
11

6

6

3

3

3

3

3

3

2

2

1

1

1

9

3
9

2

2

1

1

1

1

1

1

1

1

1

1

1
4

2

2

2

1

1

1

1

2

2

2

2

2

2

2

2

2

119

20

20

20

2

2

2

18

18

14

1

13

1

1

2

2

1

1

99

99

99

11

11

11

11

11

88
1

86
9

8

8

8

1

1

1

2

1

1

1

1

5

5

5

5

54

1

1

53

53
50

3

7

7

7

1

1

1

1

1

4514

296
4510

1523

1523

1

1

1

1

1522

1522

1522

6
2689

10

10

10

10

2673

1142

1140

1140

1140

2

2

2

8
697

13

13

3

3

10

10

6

670

670

669

1

1

1

1

1

1

833

833

833

833

833

2

2

2

2

2

4

1

1

1

3

2

2

2

1

1

1

343485
18836

1555

1555

1555

1555
217

536

536

536

802

802

802

46778

1
46771

46770

46769
3

46765

46762
3340

1143

1140

2
1140

1138

3

3

3

997
14

784

784

784

784

199

199

199

199

36356
2555

2
21878

3

2

2

2

1

1

1

372
21871

7
2

1

1

4

2

2

2

2

979
167

380
5

375

432

432

566

566

566

1
19936

19935
287

17645

1

2001

1

11

4

4

7

1

6

2

2

2

2

3556
67

1071
18

475

475

475

2
578

574

574

1

1

1

1

2418
52

646

2

2

644

644

1711

3

3

7
1708

1700

1

5

2

2

3
2

1

4

4

4

3

3

1

1

2

2

6183
343

136
1639

463

463

463
4

458

1

564

564

564

476

475

475

1

1

1567

1567

1567

1567
589

1

382

243

352

2632
164

568

568

568

3
568

565

565
4

561

3

2

2

1

1

1

1

10

1

1

1

9

9
2

7
6

1

1492

2

2

2

1490
165

1

1

1

602

602

602

602

160

160

160

562

562

562

1
395

394

393

391

2

1

1

1

2

2

2

2

6

6

4

4

4

1

1

1

1

1

1

1

560

1

1

1

1

559
4

12

1

1

2

2

8

8

1

1

1

542

2

2

540

540

1615
86

5

5

3

3

2

2

593

2
593

589

589

589

2

2

2

16

1

1

1

1

14
6

1

1

1

7

2

2

2

5

2

3

915

915

2

2

913
535

49

329

91
1

3

3

3

3

5

2

2

2

1

1

1

1

2

2

2

76

76

9

9

7

7

2

67
1

64

59

59

3
1

2

1

1

1

1

2

1

1

1

1

6

6

6
2

4

4

4835

1

1

1

1

1

4833

4833

4833

4833

1

1

1

2

2

2

2

1

1

1

1

1

1

1

1

1

1

1

1

1

1

1

7

3

3

3

3

3

3

4

1

1

1

1

1

1

3

3

3

3

3

13
100971

1482
100941

690

690

1

1

1

689

689

1
689

685

3

5

4

1

1

1

1

3

3

3

2

1

1

1

1

1

1

1418

1418

1

1

1

1

1

33
1416

348

348

348

2

1

1

1

1

1

1

4

3

3

1

1

2
1029

1027

1027
1

1026

11377
602

5
2

2

2

2

2

1

1

1

1

1
3483

1

1

3478
85

2
2464

2
2458

2456

2

1

1

2

2

5
917

900
6

894

8

8

1

1

3

3

2

1

1

1

1

1

1

6

1

1

1

5

5

5

3

3

3

1

1

2

2

2

6
725

1

1

1

2

2

2

2

20

17

17

17

17

1

1

1

1

1

2

1

1

1

1

1

1

1

1

1

2

2

2

2

2

2

2

2

2

690

690

690

690

1

1

1

1

2
1710

1

1

1

1

2

2

2

2

1

1

1

1

6

6

1

1

5

5

1697
159

2

2

18

17

17

1

1

1

577

577

577

941

940

940

1

1

1

1

1

1

1

118
4852

876

876
3

1

1

1

871

871

871

1

1

1

126
3858

993

980

980

980

980

1
980

979

5

2

2

2

3

3

3

8

1

1

6

6

1

1

2
720

689

689

689

1
29

9

9

14

1

13

5

5

1

1

1

1

1

1

1

2017

2016

2016

14

2002

1

1

1

56
85969

7

1

1

1

1

4

1

1

1

3

2

1

1

1

1

1

1

85124
5

85109
399

68
6523

4574

4574

1881

1881

78187

78187

78187

1

1

1

1

2

1

1

1

1

1

1

1

5
4

1

1

1

1

2

2

2

2

2

1

1

780

780

780
1

779

5

774

13

3

3

3

3

2

2

1

1

1

1

1

1

1

1

1

1

8

1

1

1

1

1

1

1

6

5

5

5

5

5

1

1

1

1

4

4

4

4

2

1

1

1

1

1

1

1

1

1

121393
2968

81364
221

777

777

777

777

777

101

101

101

101

101

101

100

1

80265
3

80260

1

1

1

1

1

74
80259

79845

26912

1154

1153

1153
123

544
5

68

68

471

471

486

486

486

486

1

1

1

1
8559

10
8558

1

1

1

1

8542

347
8542

8009

186

5

5

2

3

17199

17197

2642
17197

3316

3316

3181
1066

102
1813

1711

302

302

366
8058

2224

4738

730

730

2

2

2

2

2

52933

2393

2393

2393

2393

1

1

1

1

1

42694

42694

42693
490

41711
2

41709
1174

385

385

2569
37975

458

458

458

28576
6863

1606
269

94

95

3

308

119

716

2

1575

1575

1888
205

1507

1507

176

176

1490
162

60

110

1158

3655

3655

2071

2071

4254

4254

3679
218

1

921

2539

2539

1495

1495

289
6372

207
475

55

78

78

135

5606
160

96

46

5304

2

2

361

1

1

360
1

359

359

359

359

282
1814

428
314

3

33

78

136
494

136

136

4

4
1

3

3

3

215

7

7

208

158

16

34

270

270

270

340

1

339

492

492

492

492

1

1

1

1

7844

2

2

2

2

461
7842

2192
198

269
1529

176
855

592
77

215

215

245

55

55

87

87

405

405

405

465

1
465

464

464

5189

5189

5189

5189
444

4559

186

1

1

1

1

1

340

2

2

1

1

1

1

1

1

1

1

338

338

338

338

338

2

336

2

2

2

2

2

431
37061

30813
152

28336
112

390

390

390

390

27834
538

12230

12230
995

7777
743

3117
50

2847

2847

220

128
834

317

317

317

317

389

389

94
1015

418

418

314

314

189

189

189

2068
119

185

1559

1021

29

193

252

60

4

205

205

2193
421

678
17

2
317

281

281

34

34

34

344

344

1094
30

581

581

581

483
69

220

220

194

194

127
1265

465

465

465

465

673

673

673

673

673

2

2

2

283
15064

1632

1632

277
1632

142

1213

1211

2

13149
3

13122

13122
490

599

12033

2
1

1

1

22

22

2325
16

6

2

2

2

4

2

2

2

2

2

2

2

2

1
1051

1049

1049

1049

1

1

1047

1047

1

1

1

1

1

1

1243
8

88

88

5
26

2

2

17
4

7

6

2

2

62

8
62

36

18

2
1147

2

2

1143

1143

3
9

5

1

3690

1

1

1

1

1

3689
28

2270

2266
8

2258

3

3

1

1

1

1391

2

2

1389

3

3

3

3

1386

1386

1385

1385

1

1

2123

2123

2123

1

1

2118
390

4
204

200

859
30

350

349

1

479

479

664

664

664

1

1

1

3

3

3

1

1

1

4

4

2
4

1

1

1

1

53950
1925

479

479

479

479

1444
49680

1

1

1

1

12295
212

1
6211

3
20

8

8

4

4

4

4

7

7

7

7

1

1

1

1

1

1

1

291
6190

206
1188

14
509

2

478

15

463
3

459

1

3
10

3

4

1

1

1

3

3

3

1

1

1

2

2

2

146
999

358

358

495

495

1

1

1

3226

3226

3226

471

471

471

6

1

1

5

4

1

1

1

1

1

5122
240

2415

502
2415

635

1278

623

623
2

621

621

3

3

3

3

3

2
768

762

762

762
2

760

4

1

1

1

1

1065

1065
414

319

319

328

1

327

1

1

3

3

6

6

4

4

2

2

1

1

1

1

1

750

749

749

749

749

749

1

1

1

1

1

1

1

1

1031
35939

7

7

7

7

7

7

1

1

1

1

1

1

27370

27370
1656

406

406

406

12140
160

14
11171

403

403

10754

10754

10754

453

453

453

356

356

356

10284
565

561

106
561

190

265

189

189

189

965

965

965

15
709

451
1

449

1

243

243

317

317

317

6978
405

5252

5252

134

134

70
1051

572

211

198

30
136

97

9

1466

148
1466

268

268

214

214

836

836

1

1

1

1

1114

1114

1114

303

6

6

2

2

294

294

1

1

6794
530

736

72
736

362

362
192

79

79

91

302

302

302

114
953

239

239

239

5

234

328

328

328

272

272

272

272

55
4564

199
4061

327

327

327

2088

2088

2087

1

405

156
405

48
194

5

5

4

123

14

55

54

1

48
476

130

130

298

298

566

566
241

21
26

5

172

16

58
111

53

448

448

448

448

448

11

11

11

11

11

733

156
733

364

364

145
364

137

82

210

3

3

2
207

204

1

3

3

3

3

2

2

2

1

1

1

1

1321
84

711

711

711

711

526

526

526

526

545

545

545

545

1

1

2278
1

2

2

1

1

1

1

6

3

3

3

3

3

1
3

2
1

1

1

1

2268

2268

2265
5

76

2

2

2

2

15
74

23

20

2

1

36

36

2184

2184

2184

2184

3
1

2

2

2

11

11

11

11

9
1

8

2

2

2

4779

1

1

1

1

1

4778

4778

4778

4778

4778

4778

2

2

2

2

2

2

2

580
236970

3319
183894

2751
117392

4143

9
4141

4080

4078

4078

3
4078

7

3

3

1256
4065

1983

826

2

2

2

2

52

1

1

1

1

41

3

3
1

2

2

38

38

4

3

1

34

34

1
10

4

4

4

4

5

5

5

1

1

3

2

2

2

2

2

1
90797

33
90780

1175
174

463

456
105

115

106
4

13

13

13

9

9

9

78

1

1

77

77

2

2

2

9

9

3

3

3

6

6

6

29

29

29

29
3

3

23

66
157

7

7

6

6

1

1

2

1
2

1

1

1

1

1

1

67

67
7

7
30

1

1

2

2

17

1

1

25

3

22

1

1

2

2

1

1

6

6

8

8
2

6

3

3

36

9

9

1
9

8

1

1

1

1

26

26

9

9

17

17

14

11

11

11

11

3

3

1

1

2

2

7

5

2

2

2

2

2

2

1

1

1

2

2

2

2

538

538

538
2

15

12

12

1

1

2

2

3

3

3

518
398

1

1

1

1

50

37

37

1

1

3

3

2

2

2
4

2

1

1

2

2

2

1

1

1

1

67

4

4

63

63

3610
89572

271

271

271

3

3

268
5

253

6

3

1

12405

12405

7
12405

23

23

12375
2318

2921

2

7134

8063

1
8063

1

1

1

1968
8061

2902

2902

2

2

1

1

4
7

3

3177

3177

4

4

65207

65207

7
65207

9
6

3

3

35

29

28

1

6

6

65156
16

1343
65134

14

9

57878

7

5883

5
1

1

2

1

1

1

16

16

16
2

4

4

10

1

2

2

5

5
16

1

1

1

1

10

6

6

6

4

4

4

8

8

2
8

6

5

5

1

1

4

4

1

1

1

19689
512

1

1

1

1

1

1

2375

13

13

13

13

2362

2361

2361

2361

1

1

1

1

13403

569
13403

10410
231

1847

1842

1841

1

1

1

1

4

4

1

1

3
2

1

1

1

1

1

1

1

5

4
1

1

1

1

1

1

1

1

1

1

1829

1829

1829
1

1828

1

1

1

1

1

1

4

4

4

2

2

2

2

1

1

1

1

1

3086

3086
1

2
3071

3069

3069

14

13

13

1

1

1

1

1

1

1

5245

5245

5245

5244

5244

1

1

2

2

2

2

2

2

2

2422

2422

2422

2422

2422
8

1

2413

3

3

2

2

2

1

1

1

10
3309

3122

3122

3122
1

2
3121

3119

1

1

1

1

1

1

4

4

4

4

4

4

46

1

1

1

1

3

3

3

3

5
1

3

3

3

1

1

2

2

2

2

35

35

35

32

3

126

110

6

6

6

6

4

1

1

3

3

6

6

6

94

9

9

85
18

3

3

1

1

15

9

6

2

2

2
10

1

7

3

3

2

2

4

4

2

2

3
5

1

1

4

3

1

8

8

1

1

4

4

2

2

1

8

1

1

1

1

4

4

4

3

3

3

8

8

2

2

1

1

1

1

3

1

2

1

1

86

74

73

73

2
73

4
3

1

1

62
5

13

44

1

1

2

1

1

1

1

1

1

1

1

1

1

11

1
11

3
6

1

1

1

2

1

1

1

1

3

3

2

1

1

1

1

4

2

2

2

2

2

2

2

2

778
30286

5
8461

2

2

2

2

2

208

1

1

1

1

207

1

1

1

1

1

1

206

198

5
198

13

13

180

180

8

8

8

8

4

1

1

1

1

1

3
1

1

1

1

1

8242

8242

8242

8242

8242

1990

8

2

1

1

1

1

1

1

3

3

3

3

3

1

1

1

1

1

1

1

1

1982

5

4

3

3

3

1

1

1

1

1

1

1976

1976

1976
1

1975

1

1

1

1

9528

242
9528

1

1

1

1

3674

1506
3674

1

1

459

459

363

363

1
560

559

785

785

1

1

1

1

1

1

555
5609

1

1

1

1

1

1

992

992

992

1357
245

437

437

675

675

2703
421

320
1598

85

1191

1191

2

1
330

1

328

4
354

350

9529
1

684
9528

3564

1

1

1

1

3563

3563
158

3066
1370

169

4

2

161

101

166

1

133

19

109

831

339

339

2519

3

3

3

3

3

2516

2

1

1

1

1

905
2514

815

815

794
99

510

185

2

2

1

1

1

1

1

1

41

33
41

7

6

6

6

1

1

1

1

1

1

2718
60

1940

216
1940

1018
10

568
1008

315

125

700
2

1

1

1
697

696

6

1

1

5

5

5

5
3

2

2

2

2

711

711

711
1

1

2

707

2

2

2
1

1

1

1
19

2

2

2

2

15

1

14

6

6

6

7

7

7

1

1

1

1

1

1

1

1

3

1

1

1

2

2

2

850

846

846

846

846

4

4

3
4

1

9

2

2

2

2

2

7

5

5

5

2

1

1

2

2

2

2

2

26254
57

43
24020

2244

2243

2243

2243

1

1

1

92
2

1
83

6

6

5

1

1

1

1

72

1

1

1

1

1

1

1

11

1

1

9

1

1

1

1

1

1

1

1

55

55

1

1

1

1

1

1

1

1

1

7

1

1

1

1

1

1

1

1

1

1

3

2

2

1

1

1

1

1

12

12

12

12

2

10

1
21629

79

4

2

2

2

2

2

2

59

15

2

2

10

10

1

1

2

2

34

3

3

1

1

1

29

29

29

4

4

4

4

6

5

5

1

1

16

16

5

3

3

3

3

3

3

5

5

5

5

3

3

2

2

21544

2

2

2

4

4

4

21538

21538

21538

2

2

2

2

2

2

1

1

2174

28

2

2

3

3

3

3

5

3

3

3

3

3

3

2

2

2

2

1

1

1

1

1

15

2
11

9

2
9

3

3

1

3

1

1

2

2

4

2
4

1

1

1

1

2

1

1

1

1

1

5
2146

47
1

46

46

13
1

1

4

7

32

32

1

1

1

1

1

2093

2093
1

2090
1

2089

2

2

1

1

1

4050

1

1

1

1

1

1

1

7

7

7

7

6

6

5

5

1

1

1

1

1

1

4042

4042

4042

4042

4042

4042

4042

4

3

3

3

1

1

2

2

1

1

1

1

1

1

1

1707

1

1

1

1

26

1

1

1

1

25

25

25

25

1680

1680

3
1675

1667

1667

1667

1667

3

3

3

3

2

2

2

2

5

5

5

5

1

4

52496

4

4

3

3

3

3

1

1

1

1

1

9
52492

5
52482

1
52046

52045

52045
79

2

2

2

20

20

20

51944

51944
2066

15

99

106

45

14

49599

431
19

321

321

321

1

303

17

91

91

91

91

1

1

1

1

1

4
266

6

6

6

6

5

1

256
2

221

221

221

221

3

3

3

3

30

2

2

2

14

14

14

13

10

10

3

3

1

1

1

6130
4

6112

1976
6112

3586

550

550

14

1

6

1

6

2
30149

25

3

3

1

1

1

2

2

2

22

2

2

2

2

20

4

4

4

16

16
1

14

1

30066
9

24

18

18

18

18

6
2

4

4

4

4

30030
15

3

2

2

2

1

1

1

29877

2

2

2

1

1

1

29874

29874

29872

2

9

9

2

2

2

7

7

7

96
13

21

20
1

19

8

11

1

1

1

1

1

61

61

61

4

4

4

4

4

26
1

12

12

11

1

13

13

13

3

2

2

2

2

1

1

1

1

33

33

24
1

23

3

5

15

1
9

8

8

18

18

14

1
3

2

2

11

11

11

4

4

3

3

3

1

1

1

5

5

2

2

1817

3

3

3

1814

1814

1814

5802

1

1

6

6

6

5795

2

2

4

4

4

5789

5789

5789

5013

6

6

5007

5007
37

4970

2440596

2440550
13814

267789
2407151

141963

141963

141963
41179

12721

594

82772

82772

4697

37497

37497

37497

37497

1

1

1

1

2031
44983

18060

18060

18060

24892

24892

2

24890

195043
7400

43138
1128

8
26079

26071

15931

15931

40370
144505

86009

86009

18126

18126

1719875
87026

1295535
70819

420478
3207

232675

4

184592

804238
74917

602618

126703

337314

337314
24609

1

142318

170303

1

82

82

4243

4243

4243

15287

15287
1481

1
4157

4156

4624

4624

5025

5025

1

1

54

54

46

46

46

46

46

46

237696
10943

222

222

222

129

129

129

93

93

93

74154
17

73

73

73

73

73

6

6

6

5

5

1

1

194

194

194

3

3

191

191

2768

692

691

48

48

177

177

27

27

439

439

1

1

1

1966

327

37

37

290

290

1639

103

103

25

25

54

54

10

10

1442

1442

5

5

110

110

110

110

58024

1261

1261

642

642

298

298

321

321

56763
682

30

30

30

220
25295

10554

10554

1257

1257

463

463

1228

1228

1204

1204

1380

1380

6931

6931

373

373

1685

1685

50
30756

250

250

145

145

4684

4684

81

81

5596

5596

59

59

10289

10289

1568

1568

1747

1747

6287

6287

35

2

2

2

2

33

33

33

33

85

85

7

7

7

78

78

78

59
12952

964

953

16

16

138

138

34

34

89

89

1

1

11

11

108

108

43

43

18

18

495

495

11

11

11

1222

160

12

12

106

106

42

42

1062

157

157

905

905

3229

3157

2748

2748

390

390

19

19

72

72

72

24

24

24

24

4472

27

27

27

1193

774

774

419

419

9
2354

400

400

164

164

80

80

256

256

121

121

52

52

91

91

262

262

127

127

263

263

293

293

236

236

480

480

480

418

101

101

298

298

19

19

419

419

419

419

1016

1016

1016

1016

431

431

431

431

329

44

27

27

17

17

285

285

285

605

605

3

3

369

369

198

198

1

1

34

34

127

127

65

65

62

62

55

55

55

55

15

15

15

15

15

15

72

72

72

72

72

72

213

213

213

18

18

18

87

34

34

53

53

108

48

48

60

60

1074

1074

411

411

411

411

663

128

76

76

52

52

58

5

5

4

4

13

13

36

36

477

5

5

22

22

450

450

2

2

2

2

2

2

26
16446

51

51

51

51

51

1347

1347

430

430

430

917

209

209

514

514

194

194

4286

440

274

274

274

93

93

93

73

73

73

3846

3846

2875

2875

171

171

800

800

10736

10736
81

9567
3

238

238

352

352

4319

4319

10

10

8

8

3665

3665

946

946

1

1

25

25

25

25

25

1063

1063

1063

1599

1599

552

215

38

38

177

177

337

41

41

5

5

20

20

60

60

211

211

194

24

24

24

122

122

122

48

34

34

14

14

180

116

116

116

64

52

52

12

12

673

564

103

103

461

461

4

4

4

105

42

42

10

10

53

53

3370

3370

10

10

7

7

3

3

968

632

1

1

9

9

195

195

10

10

33

33

384

384

5

5

5

175

145

145

4

4

23

23

3

3

133

133

133

23

13

13

10

10

37

37

36

36

1

1

1040

5

5

5

168

168

168

17

17

17

21

21

21

197

197

197

1

1

1

12

12

12

614

45

45

565

565

4

4

5

5

5

400

400

400

400

308

308

5

5

19

19

119

119

3

3

162

162

607

39

19

19

9

9

11

11

568

3

3

565

565

2036

2036

1268

152

152

152

1116

33

33

1083

1083

480

4

4

4

141

141

141

3

3

3

90

90

90

6

6

6

215

160

160

40

40

15

15

11

11

11

10

10

10

4

4

1

1

3

3

284

275

2

2

273

273

9

9

9

14637

63
14637

3392

79

15

15

55

55

9

9

3313

3313

3313

63

63

20

20

43

43

110

110

110

110

122

122

122

122

2619

8

8

8

2

2

2

22

22

22

68

68

68

51

51

51

22

22

22

731

165

165

5

5

129

129

3

3

7

7

3

3

38

38

16

16

185

185

153

153

27

27

899

99

99

136

136

134

134

5

5

4

4

2

2

79

79

10

10

116

116

5

5

309

309

177

177

177

94

94

94

257

233

233

19

19

4

4

1

1

59

59

59

229

127

127

2

2

52

52

48

48

15

15

15

15

4
6969

147

147

147

664

188

188

312

312

7

7

157

157

225

137

137

88

88

296

296

296

20

20

20

328

75

75

2

2

251

251

19

19

19

671

671

671

161

21

21

140

140

16

16

16

646

11

11

98

98

125

125

180

180

163

163

8

8

11

11

50

50

2

2

2

51

14

14

37

37

139

16

16

123

123

23

23

23

13

9

9

4

4

62

62

62

1

1

1

365

6

6

64

64

177

177

118

118

411

411

411

523

523

523

267

267

267

16

16

16

54

54

54

109

109

109

13

13

13

9

9

9

57

57

57

9

9

9

32

12

12

20

20

729

9

9

532

532

21

21

16

16

128

128

23

23

206

206

206

297

296

296

1

1

3

3

3

381

381

381

11

11

5

5

1

1

5

5

31

31

7

7

24

24

253

253

251

251

2

2

973

7

7

7

67

67

67

6

6

6

12

12

12

176

176

176

24

24

24

31

31

31

99

10

10

89

89

385

385

385

8

8

8

158

158

158

16

16

16

16

252

252

252

113

113

113

139

139

139

555
51114

8
10366

5464

592

4

4

493

493

33

33

3

3

59

59

293

293

293

543

543

543

1894

69

69

121

121

1

1

37

37

11

11

3

3

4

4

185

185

178

178

2

2

4

4

80

80

2

2

31

31

60

60

159

159

947

947

2142

102

102

692

692

83

83

860

860

405

405

257

124

124

124

133

133

133

23

23

23

23

101

9

9

9

92

56

56

36

36

2655

11

11

11

95

95

95

108

91

91

17

17

2267

9

9

164

164

2094

2094

174

151

151

23

23

1085

1085

269

269

579

579

48

48

2

2

95

95

92

92

773

773

544

544

227

227

2

2

258

258

36

36

36

18

18

18

35

35

35

169

169

169

13

13

13

13

13

616

9

9

9

9

607

33

33

33

242

242

242

332

323

323

9

9

14923
18

8225

120

30

30

87

87

2

2

1

1

484

342

342

47

47

35

35

60

60

43

25

25

18

18

390

90

90

16

16

120

120

17

17

19

19

11

11

16

16

70

70

9

9

22

22

2380
463

51

51

16

16

18

18

412

412

16

16

102

102

194

194

18

18

186

186

904

904

48

48

48

264

10

10

83

83

165

165

2

2

4

4

3754

883

883

107

107

2588

2588

176

176

742

256

256

419

419

20

20

47

47

583

583

560

560

19

19

4

4

40

40

12

12

28

28

2630

1

1

1

605

345

345

260

260

2024

227

227

917

917

60

60

713

713

107

107

701

178

174

174

4

4

523

523

523

2676

91

77

77

14

14

154

154

154

83

83

83

2320

2284

2284

13

13

23

23

1

1

1

27

27

27

50

50

34

34

16

16

2
22524

20969
225

209

209

209

460

460

460

677

344

344

333

333

1676

1676

1676

115

115

115

15096
52

72

72

63

63

4

4

33

33

43

43

2072

2072

42

42

1325

1325

596

596

1242

1242

7020

7020

216

216

1088

1088

1228

1228

575

92

92

444

444

39

39

1759

610

610

480

480

14

14

525

525

84

84

13

13

33

33

21

21

21

156

156

156

111

87

87

87

5

5

5

19

19

19

185

185

4

4

134

134

47

47

13

13

13

13

1244

314

194

194

23

23

97

97

148

148

148

782

56

56

322

322

59

59

345

345

1859

6

6

6

6

1339

1177

340

340

821

821

11

11

5

5

162

162

162

23

22

22

22

1

1

1

491

22

22

22

87

64

64

23

23

382

382

382

446

446

446

205

205

205

8

8

8

201

201

201

8

6

6

2

2

24

24

24

745

745

745

745

268

268

71

71

19

19

5

5

62

62

212

212

108

108

6057

21

21

21

21

21

4142

4142

4142

6

6

84

84

4

4

296

296

32

32

3477

3477

130

130

10

10

56

56

47

47

1618

587

3

3

3

7

7

7

576

576

576

1

1

1

1031

42

42

42

344

5

5

339

339

645

573

573

43

43

29

29

276

128

128

1

1

127

127

90

29

29

29

61

61

61

42

42

42

42

16

16

16

16

53336
56

3667

3222

3204

111

111

20

20

3

3

505

505

1107

1107

597

597

861

861

18

18

18

445

445

445

445

36

36

6

6

6

30

30

30

11128

403
11128

2140

5

5

1

1

757

757

179

179

619

619

110

110

469

469

474

25

25

110

110

252

252

56

56

22

22

9

9

65

65

65

1404

1020

1020

384

384

148

12

12

136

136

4012

3901

3901

111

111

2482

9

9

12

12

2461

2461

981

981

303

303

303

678

678

678

5

5

5

5

5

37
12313

2269

3
2269

1371

1371

3

3

2

2

812

812

58

58

1

1

19

19

10007
24

486

52

52

27

27

407

407

872

872

872

2818

2818

2818

73

1

1

72

72

5240
101

7

7

272

272

1948

1948

83

83

2088

2088

90

90

187

187

269

269

3

3

192

192

494

490

490

4

4

713

713

713

689

689

24

24

2319

2049

5

5

5

2044

2

2

172

172

12

12

96

96

413

413

8

8

1341

1341

270

270

270

270

22118

407
22118

67

67

67

1017

1017

1017

1
5770

1

1

505

505

75

75

164

164

354

354

149

149

300

300

1

1

356

356

1699

1699

11

11

783

783

91

91

822

822

11

11

299

299

78

78

5

5

24

24

41

41

5

5

5

46

46

46

5

5

5

1664

1664

1664

537

195

195

342

342

647

182

182

1

1

6

6

326

326

84

84

48

48

128

128

128

27

27

27

71
3394

237

237

89

89

728

728

59

59

7

7

38

38

50

50

537

537

137

137

24

24

442

442

87

87

1

1

17

17

10

10

385

385

369

369

106

106

246

246

246

1

1

1

227

227

227

7930
8

152

152

108

108

390

390

379

379

39

39

287

287

562

562

422

422

99

99

3900

3900

37

37

137

137

111

111

224

224

241

241

65

65

44

44

398

398

3

3

136

136

188

188

963

14

14

14

14

14

949

949

466

466

466

37

37

37

352

258

258

34

34

60

60

94

94

94

10220991
2614965

5

5

5

5

5

5

12301

5816

5816

29

14

14

15

15

5787

20

20

2695

2695

675

675

572

572

1113

1113

80

80

33

33

6

6

593

593

169

169

133

133

133

36

36

36

6316

120

120

120

120

138

138

138

138

6058

1

1

1

38
6057

254

254

300

300

42

42

199

199

35

35

5189

5189

7166

3093

2586

13

13

13

2573

2165

2165

206

206

202

202

147

147

146

146

1

1

9

9

9

9

351

259

237

237

22

22

1

1

1

82

82

82

9

9

9

3799

1976

956

63

63

893

893

689

1

1

688

688

331

199

199

132

132

1823

1744

1612

1612

126

126

6

6

79

13

13

25

25

4

4

37

37

2

2

2

2

2

272

17

17

17

17

255

255

11

11

133

133

111

111

28

28

28

14

3

3

11

11

14

14

14

2473

36

36

36

1

1

35

35

268

233

233

233

233

35

35

35

35

126

126

3

3

3

123

103

103

20

20

23

23

23

23

23

483

206

19

19

19

187

181

181

6

6

277

244

244

244

1

1

1

32

27

27

5

5

1424
4

85

85

85

85

881

646

75

75

2

2

179

179

281

281

39

39

70

70

52

52

52

62

62

62

121

121

121

454

1

1

1

453

18

18

11

11

54

54

56

56

254

254

60

60

113

79

79

79

79

34

34

1

1

33

33

175

175

175

175

175

175

7704

1713

1713

1713

1713

1713

597

597

418

418

418

179

179

179

304

203

203

203

203

85

85

85

85

1

1

1

1

15

15

15

15

5090

3532

3532

283

283

16

16

37

37

13

13

1624

1624

339

339

261

261

959

959

1147

952

583

583

114

114

255

255

195

35

35

16

16

144

144

411

411

411

411

63

63

63

63

63

63

14

14

14

14

14

14

2672

453

453

453

453

453

34

34

34

34

34

1517

1517

1517

56

56

1461

1461

568

568

568

126

126

442

442

100

100

100

100

100

58610
236

1209

1209

1209

1012

1012

197

197

89

89

89

89

89

3869

708

708

51

51

5

5

491

491

161

161

8

1

1

1

7

7

7

551

551

551

551

80

80

80

80

92

92

53

53

39

39

679

679

45

45

409

409

128

128

97

97

4

4

4

4

1747

1747

1190

1190

161

161

316

316

3

3

18

18

8

8

30

30

21

21

924

924

924

924

924

873

230

230

230

230

643

643

541

541

102

102

11939

229

229

34

34

195

195

11710

420

414

414

6

6

15
10279

301

301

399

399

352

352

2121

2121

3860

3860

193

193

1036

1036

5

5

306

306

1691

1691

137

70

70

67

67

107

107

107

767

588

588

144

144

35

35

16874

1227

310

91

91

140

140

79

79

917

382

382

447

447

88

88

238

24

24

24

214

214

214

738

186

186

186

552

530

530

22

22

112

112

112

112

41
10578

5130

2

2

48

48

78

78

256

256

227

227

742

742

59

59

1147

1147

1182

1182

326

326

71

71

35

35

34

34

16

16

40

40

26

26

329

329

7

7

12

12

119

119

138

138

236

236

45
2992

1716

1716

1231

1231

2173

536

536

91

91

1069

1069

450

450

27

27

237

3

3

234

234

5

5

5

1499

166

166

166

966

39

39

28

28

755

755

16

16

128

128

367

367

367

1915

1915

9

9

41

41

1

1

16

16

233

233

8

8

1607

1607

567

567

57

57

510

510

144

144

144

144

144

4845
1

2066

1940

67

67

360

360

4

4

2

2

1471

1471

18

18

9

9

9

9

126

75

75

51

51

1091

1091

21

21

1026

1026

44

44

1566

1566

317

317

49

49

1188

1188

12

12

121

121

53

53

68

68

279

279

279

264

264

15

15

6293

1311

1311

39

39

1269

1269

3

3

4982

649

20

20

629

629

1782

1352

1352

380

380

50

50

267

13

13

241

241

13

13

2168

51

51

884

884

875

875

16

16

342

342

116

116

116

10824

164

164

144

144

20

20

10660
25

1795

298

298

424

424

69

69

4

4

2

2

838

838

74

74

7

7

79

79

385

25

25

186

186

86

86

8

8

80

80

8455
200

122

122

1897

1897

1675

1675

4561

4561

212

212

212

182

182

30

30

88

88

1

1

1

1

87

87

87

87

18

18

18

18

6

6

12

12

167

167

167

123

23

23

12

12

88

88

44

44

44

668

668

2

2

2

2

617

353

326

326

27

27

264

264

264

49

49

49

49

364

364

364

10

10

10

354

354

354

5

5

5

5

5

5

1083
167373

7

7

7

7

7

5

5

2

2

2

3

3

3

271
22333

9214
2

4368
7

126

126

56

56

43

43

33

33

62

62

30

30

327

327

1754

1754

155

155

998

998

7

7

1

1

769

769

4023

3220

3220

803

803

821

821

821

12848
20

1090

536

536

554

554

2906

160

160

51

51

187

187

1992

1992

453

453

55

55

8

8

6

6

6

11

11

11

93

93

93

1113

1113

1113

1
874

5

5

228

228

398

398

42

42

56

56

2

2

53

53

85

85

4

4

447

146

146

160

160

12

12

129

129

2948

356

356

972

972

1620

1620

2922

2602

2602

320

320

418

124

124

22

22

272

272

47

47

47

18

18

29

29

1231

1231

346

346

346

885

819

819

66

66

1880

998

829

283

283

106

106

399

399

41

41

38

38

38

131

131

131

882

606

47

47

151

151

44

44

364

364

276

145

145

117

117

14

14

18071

18071

2

2

2

378

303

303

75

75

1611

1611

1611

53

53

53

4964

2895

2895

360

360

536

536

263

263

910

910

241

11

11

29

29

201

201

1687

1687

1687

3552

3552

3552

19

19

19

24

24

24

258

258

258

2751

2519

2519

227

227

3

3

2

2

445

362

362

65

65

18

18

1

1

1

728

728

728

1212

1212

1212

145

145

145

9097
1

893

893

217

217

676

676

1688

1688

11

11

126

126

828

828

25

25

562

562

136

136

2598

2090

43

43

346

346

1701

1701

508

39

39

469

469

748

748

260

260

295

295

2

2

191

191

831

831

145

145

686

686

995

988

988

988

7

7

7

1343

1343

637

637

33

33

673

673

1680

1680

7

7

7

52

52

52

44

44

44

750

45

45

5

5

650

650

50

50

5

5

5

822

74

74

748

748

3
36313

5432

359

8

8

113

113

231

231

7

7

96

96

96

4185

574

574

569

569

205

205

9

9

19

19

33

33

75

75

1811

1811

685

685

77

77

7

7

121

121

792

266

266

526

526

1020

162

162

162

13

13

13

845

99

99

209

209

22

22

404

404

111

111

18
29858

7

7

7

88

88

88

38

38

38

216

186

186

30

30

1730

1

1

893

893

7

7

829

829

190

190

190

123

116

116

7

7

13

13

13

472

472

472

233

233

233

7643

453

453

6639

6639

200

200

351

351

21

21

21

181

181

181

1

1

1

1

1

1

652

129

129

11

11

4

4

53

53

97

97

108

108

250

250

33

16

16

17

17

559

88

88

269

269

202

202

86

86

86

591

591

591

553

553

553

767

132

132

635

635

4453

6

6

864

864

18

18

1

1

1149

1149

2284

2284

76

76

32

32

1

1

22

22

208

208

208

864

864

864

5158

1200

1200

18

18

874

874

1728

1728

1338

1338

143

143

143

233

233

233

35

35

35

569

149

149

417

417

3

3

258

258

258

2

2

2

27

27

27

3692

2810

2810

21

21

861

861

531

531

531

531

531

2870

2747

2747

332

332

188

188

703

703

1029

1029

495

495

123

123

73

73

50

50

28303

28303

1654

1311

1311

343

343

1657

1635

1635

22

22

858

858

858

7

7

7

3203
20017

1514

1514

554

554

179

179

898

898

386

386

328

328

3

3

4737

4737

178

178

5

5

37

37

411

411

150

150

341

341

808

808

59

59

564

564

316

316

2192

2192

218

218

79

79

2

2

339

339

767

767

8

8

63

63

88

88

1153

1153

437

437

2606

45

45

2545

2545

2

2

14

14

1489

1489

1489

15

15

15

1261

1261

113

61

61

52

52

27

27

27

303

303

303

818

76

76

742

742

2910

1978

1978

32

32

137

137

927

927

33

33

504

504

345

345

112

6

6

6

106

106

106

820

37

11

11

26

26

687

370

370

289

289

28

28

96

96

96

997

619

619

619

619

378

378

23

23

32

32

214

214

15

15

94

94

4002

4002

7

7

7

3539
3

424

424

1614

1614

1498

1498

456

352

352

104

104

1541

292

292

292

292

658

476

49

49

427

427

20

20

20

162

66

66

71

71

25

25

591

591

591

591

24910

24910

3440

66

66

251

251

33

33

125

125

610

610

1071

1071

7

7

99

99

696

696

103

103

366

366

13

13

6755

820

820

1

1

2027

2027

28

28

8

8

510

510

522

522

675

675

1193

1193

156

156

361

361

26

26

77

77

351

351

216

216

216

14499

559

559

139

139

9396

9396

1086

1086

543

543

1131

1131

1645

1645

1304

269

269

67

67

71

71

40

40

91

91

1035

1035

716

716

259

259

11

11

16

16

33

33

880

848

836

28

28

666

666

114

114

28

28

12

12

12

32

32

30

30

2

2

3659

3659

2788

22

22

883

883

268

268

850

850

226

226

173

173

38

38

22

22

306

306

871

824

824

47

47

2458

19

19

19

19

2439

1497

7

7

286

286

112

112

205

205

93

93

9

9

5

5

161

161

548

548

71

71

942

158

158

54

54

729

729

1

1

179733
2621

126

21

21

21

21

105

105

105

105

1281

693

693

693

693

507

507

507

507

81

81

81

81

1228

762

762

647

647

115

115

421

421

421

421

45

38

38

38

7

7

7

14

14

14

14

14

145

135

135

2

2

133

133

10

10

10

10

4935

606

606

606

606

24

24

24

24

1680

1680

56

56

443

443

944

944

237

237

2625

1129

480

480

239

239

410

410

1488

410

410

51

51

966

966

22

22

24

24

15

15

8

3

3

5

5

24
48225

1214

1214

26

26

80

80

129

129

510

510

469

469

410

410

359

359

51

51

4

4

4

4

5450

1001

937

937

12

12

16

16

1

1

35

35

1872

49

49

560

560

747

747

1

1

462

462

53

53

764

282

282

279

279

203

203

1813

693

693

326

326

60

60

8

8

395

395

331

331

2918

595

61

61

479

479

55

55

2323

54

54

86

86

1

1

78

78

70

70

164

164

1089

1089

67

67

14

14

230

230

244

244

226

226

1644

1644

13

13

68

68

9

9

606

606

857

857

53

53

38

38

159

19

19

19

140

128

128

12

12

9740
41

3229

264

264

45

45

756

756

31

31

174

174

43

43

69

69

115

115

119

119

61

61

556

556

996

996

121

5

5

116

116

1803

1803

1803

3834

268

268

2454

2454

411

411

332

332

17

17

134

134

22

22

184

184

12

12

712

574

574

130

130

8

8

26662

694
22808

368

368

289

289

1

1

1302

1302

22

22

112

112

110

110

98

98

356

356

798

798

358

358

559

559

361

361

119

119

72

72

18

18

540

540

144

144

2080

2080

124

124

423

423

315

315

1803

1803

144

144

1343

1343

40

40

146

146

301

301

156

156

864

864

1471

1471

8

8

213

213

51

51

135

135

3328

3328

2109

2109

212

212

111

111

280

280

112

112

206

206

62

62

112

112

57

57

50

50

52

52

93

93

86

86

3854

96

96

3

3

2031

2031

82

82

57

57

151

151

608

608

101

101

263

263

332

332

130

130

242

1

1

1

1

241

241

241

241

926
99707

5047

5047

706

706

715

715

889

889

2

2

35

35

298

298

2402

2402

24214

227

227

227

49

17

17

32

32

13
23938

183

183

853

853

2

2

28

28

339

339

496

496

1123

1123

644

644

257

257

605

605

749

749

45

45

565

565

331

331

712

712

272

272

175

175

1876

1876

8595

8595

4

4

2919

2919

17

17

23

23

882

882

57

57

2173

2173

14648
22

11650
5

2

2

794

794

70

70

45

45

2297

2297

74

74

69

69

313

313

1166

1166

197

197

519

519

80

80

304

304

4975

4975

317

317

33

33

390

390

2976

22

22

2954

2954

202

202

202

202

354
54670

324
30833

1907

1907

105

105

17

17

679

679

125

125

25

25

3644

3644

3

3

1277

1277

2005

2005

35

35

932

932

51

51

62

62

283

283

77

77

35

35

675

675

761

761

124

124

6494

6494

637

637

366

366

78

78

136

136

397

397

511

511

4317

4317

991

991

227

227

2528

2528

587

587

148

148

270

270

10

10

10

793

775

775

13

13

5

5

8489
5

25

25

2008

2008

1301

1301

3999

3999

48

48

1

1

542

542

560

560

470

463

463

4

4

3

3

98
6318

958

958

1761

1761

92

92

12

12

394

394

617

617

1134

1134

43

43

119

119

1090

1090

3565

284

284

24

24

353

353

369

369

1431

1431

57

57

495

495

368

368

14

14

170

170

2204

2204

2204

251

251

251

1383

855

855

72

72

69

69

387

387

3270

3270

3270

105

105

158

158

1181

1181

21

21

663

663

633

633

509

509

24

24

24

24

24

62

62

62

13

13

49

49

957

670

670

670

670

287

287

281

281

6

6

9659

1909

378

304

304

74

74

1531

361

361

2

2

511

511

275

275

382

382

392

25

25

25

367

367

367

6511

1427

497

497

223

223

72

72

75

75

134

134

113

113

307

307

6

6

89
5084

171

171

37

37

79

79

88

88

29

29

117

117

182

182

367

367

22

22

54

54

788

788

14

14

321

321

223

223

727

727

1660

1660

14

14

6

6

96

96

847

847

847

847

1951

1951

49

49

49

191

191

191

1711

531

531

1102

1102

3

3

75

75

19

19

19

19

19

19

19

19

19

19

5248

112

112

112

112

975

960

960

960

15

15

15

4161

4161

934

934

2781

2781

86

86

360

360

578

578

578

578

35

35

148

148

395

395

471

471

471

420

420

420

51

51

51

12
46391

169

47

47

47

47

122

122

122

122

2956

2956

2956

2956

2956

4880

4851

422

422

422

1242

1035

1035

207

207

3187

3187

3187

29

29

29

29

1023

1023

515

33

33

1

1

481

481

5

5

5

503

5

5

26

26

45

45

7

7

74

74

346

346

559
31080

3

3

3

3

5
13117

1763

970

970

11

11

117

117

341

341

301

301

23

23

118

118

118

230

33

33

6

6

84

84

107

107

11001
72

46

46

125

125

480

480

7418

7418

18

18

81

81

274

274

29

29

58

58

470

470

1550

1550

380

380

60

60

60

60

637

637

607

607

30

30

77

77

8

8

69

69

3

3

3

3

339

63

63

63

276

276

276

312

312

312

312

291

291

287

287

4

4

111

31

31

31

80

80

80

9327
10

227

227

227

128

30

30

47

47

51

51

1357

164

164

597

597

9

9

587

587

203

203

203

1301

1187

1187

114

114

689

284

284

350

350

55

55

19

19

19

567

567

567

4112

4112

4112

553

413

413

140

140

161

7

7

154

154

4119

4119
7

178

178

17

17

483

483

373

373

36

36

77

77

131

131

82

82

169

169

31

31

1630

1630

790

790

100

100

15

15

1495

1204

1204

1204

66

66

66

221

34

34

187

187

4

4

4

318

318

44

44

274

274

312

312

312

312

245

245

245

1

1

244

244

18

18

18

18

18

264

264

264

247

247

14

14

3

3

5574

5538

57

56

56

1

1

5284

5

5

5213

5213

66

66

197

150

150

47

47

36

36

36

36

170

170

170

170

170

7211
3

5985

4866

599

599

599

970

970

970

3297

218

218

501

501

2112

2112

176

176

290

290

1119

4
1119

625

625

9

9

90

90

82

82

302

302

7

7

1223

1223

12
1223

26

26

764

764

181

181

240

240

176

176

176

165

80

80

85

85

11

11

11

5869

5869

5869

1009

7

7

52

52

299

299

39

39

144

144

234

234

234

234

179

78

78

101

101

3702

43

43

1589

1589

1328

1328

360

360

322

322

60

60

46

12

12

34

34

7

7

7

283

193

193

50

50

40

40

392

392

392

251

251

251

338

338

338

338

146

146

27

27

165

165

29

29

29

29

29

29

293

293

293

293

293

293

7984

7984

32

32

32

32

5487

10

10

10

5477

216

216

4

4

9

9

939

939

1

1

45

45

540

540

712

712

709

709

824

824

344

344

946

946

17

17

47

47

124

124

2465

63

63

63

1432

1432

1432

970

970

970

3

3

3

3

3

3

122

122

122

118

97

97

21

21

4

4

4

812

812

812

812

812

812

27

27

27

27

27

27

1001

378

378

378

344

344

34

34

623

623

251

74

74

70

70

107

107

372

370

370

2

2

100

100

100

100

100

100

13

13

13

13

13

13

5230

37

37

37

37

37

4514

4159

3726

108

108

46

46

183

183

49

49

54

54

156

156

3

3

47

47

53

53

844

844

649

649

234

234

31

31

24

24

95

95

505

505

101

101

25

25

315

315

200

200

4

4

433

433

433

355

355

246

246

102

102

5

5

2

2

679

45

45

45

45

30

30

30

30

604

604

367

367

237

237

361

7

7

7

7

7

290

290

290

290

290

64

64

55

55

55

9

9

9

25
21087

1105

1105

1105

431

431

4

4

670

670

898

898

898

793

793

105

105

773

3

3

3

3

770

770

770

770

1300

35

35

35

35

1265

527

209

209

2

2

57

57

259

259

92

10

10

82

82

513

513

513

97

97

97

36

36

36

1290

455

455

29

29

46

46

157

157

223

223

835

2

2

2

92

4

4

79

79

9

9

114

90

90

24

24

7

7

7

620

414

414

14

14

192

192

2111

2111

2111

2101

2101

10

10

18

18

18

18

18

6931

6931

6931

24

24

6

6

9

9

81

81

8

8

226

226

561

561

5

5

239

239

84

84

77

77

38

38

29

29

490

490

12

12

53

53

4231

4231

3

3

51

51

5

5

198

198

3

3

458

458

9

9

31

31

6636

5347

126

50

50

76

76

1092

514

514

95

95

45

45

40

40

80

80

301

301

17

17

133

133

133

1
3974

54

54

227

227

1480

1480

11

11

2

2

14

14

11

11

87

87

1941

1941

15

15

21

21

2

2

11

11

35

35

62

62

22

14

14

8

8

1071

2

2

2

192

192

192

122

122

122

755

42

42

14

14

116

116

28

28

20

20

228

228

11

11

37

37

9

9

29

29

215

215

6

6

218

207

207

207

11

11

11

117
234282

215484
2114

756

756

756

756

56448
4292

505

20

20

189

189

148

148

148

148

1014

233

233

181

181

126

126

211

211

245

245

18

18

2773

196

196

248

248

10

10

2307

2307

12

12

877

77

77

3

3

70

70

727

727

1224

1224

1224

1394

4

4

1331

1331

27

27

32

32

2377

8

8

516

516

77

77

290

290

988

988

498

498

14392
513

2671

2671

186

186

55

55

822

822

98

98

327

327

98

98

102

102

2286

2286

127

127

121

121

500

500

33

33

10

10

376

376

228

228

1071

1071

617

617

193

193

1047

1047

821

821

104

104

3

3

34

34

7

7

1427

1427

515

515

733

733

733

6447
1

15

15

239

239

475

475

303

303

169

169

3783

3783

789

789

483

483

190

190

2
8405

262

262

3848

3848

62

62

1929

1929

191

191

446

446

6

6

1659

1659

1096

776

776

320

320

622

439

439

5

5

16

16

26

26

136

136

486

486

486

48

48

48

408

408

408

35

35

35

1348

540

540

126

126

682

682

5648

683

683

511

511

3307

3307

1147

1147

2324

1612

1612

624

624

88

88

425

300

300

300

125

90

90

35

35

71

71

52

52

19

19

440

440

440

440

1449

913

270

270

643

643

536

536

536

583

583

583

583

416

416

416

416

197

197

4

4

126

126

67

67

8774

8774

17

17

2885

2885

21

21

159

159

1673

1673

414

414

13

13

59

59

3018

3018

515

515

89

89

89

89

1447

1447

57

57

22

22

11

11

136

136

115

115

254

254

38

38

522

522

37

37

3

3

252

252

2943

2943

479

479

18

18

781

781

364

364

777

777

122

122

13

13

18

18

92

92

13

13

266

266

3870

3870

116

116

5

5

4

4

41

41

2318

2318

1219

1219

167

167

132181

4190
132181

87

87

87

87

3868

3868

1572

1572

193

193

86

86

29

29

500

500

427

427

1145

1145

76

76

355

355

511

511

912

912

43

43

49

49

52

52

529

529

2256

2256

325

325

46

46

1180

1180

309

309

1116

1116

1623

1623

58

58

9

9

239

239

1600

1600

443

443

89

89

226

226

163

163

86980

86980

165

165

188

188

103

103

94

94

318

318

2403

2403

5

5

9378

9378

220

220

6247

6247

1687

1687

3281

3281
5

439

439

4

4

108

108

470

470

16

16

4

4

2192

2192

8

8

35

35

17969

31

31

7

7

14

14

10

10

93

93

93

93

15908

2600

25

25

1665

1665

910

910

196

196

196

13112

7205

7205

291

291

40

40

159

159

1914

1914

592

592

18

18

380

380

403

403

64

64

72

72

44

44

446

446

118

118

138

138

24

24

163

163

39

39

389

389

613

613

1163

13

3

3

1

1

9

9

1150

952

952

137

137

61

61

774

134

127

127

7

7

640

398

398

125

125

13

13

45

45

39

39

20

20

712

314

2

1

1

1

1

312

312

312

23

23

23

23

375

21

21

21

354

354

354

1047
159632

31
63635

204

204

200

200

4

4

1045

1045

1001

1001

7

7

31

31

6

6

1063

607

64

64

66

66

477

477

206

14

14

192

192

250

135

135

115

115

10

10

10

10

222

222

222

222

6882

1070

2

2

42

42

646

646

224

224

142

142

14

14

1334

1197

1197

137

137

4478

1776

1776

393

393

273

273

919

919

24

24

1093

1093

4515

553

22

22

105

105

45

45

300

300

76

76

5

5

3562

14

14

1089

1089

1072

1072

2

2

513

513

872

872

18

18

18

328

328

328

54

54

54

162

66

66

66

96

96

96

469

298

58

58

240

240

157

157

157

14

14

14

1629

1629

6

6

1623

1623

18

18

18

18

239

239

239

239

8069

716

108

108

321

321

1

1

286

286

216

205

205

11

11

3466

923

923

776

776

895

895

212

212

10

10

275

275

14

14

283

283

60

60

4

4

1

1

13

13

3544
3

395

395

509

509

2637

2637

107

7

7

55

55

34

34

11

11

20

20

20

2215

10

10

10

595

147

147

448

448

1610

1610

1610

84

84

84

84

132
32831

787

79

79

343

343

365

365

4362

41

41

154

154

114

114

1143

1143

524

524

9

9

156

156

51

51

437

437

71

71

607

607

15

15

47

47

23

23

970

970

1285

923

923

362

362

18

3

3

15

15

581

581

581

78
12493

15

15

415

415

215

215

48

48

20

20

408

408

11

11

91

91

1254

1254

312

312

644

644

11

11

1430

1430

445

445

132

132

158

158

44

44

22

22

35

35

195

195

947

947

1725

1725

3727

3727

2

2

2

2

39

39

65

65

3

3

1240

585

585

394

394

257

257

4

4

7794
1

1594

1594

607

607

650

650

159

159

40

40

17

17

937

937

204

204

189

189

113

113

145

145

13

13

195

195

1178

1178

1752

1752

137

137

137

971

971

971

831

13

13

52

52

141

141

63

63

562

562

1232

31

31

79

79

424

424

676

676

22

22

968

23

23

127

127

818

818

75

7

7

7

68

10

10

58

58

2141

2141

34

34

8

8

424

424

327

327

5

5

135

135

382

382

350

350

68

68

408

408

91

91

91

91

3

3

3

3

174

99

99

99

75

75

75

703

658

7

7

15

15

214

214

67

67

19

19

51

51

158

158

35

35

11

11

81

81

45

45

45

226

144

77

77

67

67

82

82

82

534

534

110

110

361

361

63

63

465

465

465

465

465

47

47

47

47

47

15746

838

838

107

107

226

226

456

456

49

49

2617
47

2343

289

289

567

567

1040

1040

1

1

446

446

227

227

227

12291

1746

1746

1746

10545

76

76

151

151

27

27

3

3

2608

2608

129

129

276

276

135

135

327

327

1

1

581

581

196

196

53

53

7

7

957

957

5018

5018

570

570

41

41

41

158

158

158

320

320

320

51

51

51

1419

705

705

44

44

243

243

418

418

714

714

714

714

230

230

230

79

79

151

151

13022
2

181
7526

2372

9

9

14

14

47

47

79

79

72

72

7

7

77

77

2067

2067

1628

1081

1081

5

5

349

349

158

158

35

35

3345

112

112

3

3

296

296

25

25

77

77

995

995

29

29

153

153

258

258

1220

1220

114

114

20

20

21

21

22

22

510

510

178

178

332

332

544

21

21

21

523

185

185

335

335

3

3

4096

2102

376

376

376

376

265

265

7

7

284

284

794

794

133

133

133

1157

32

32

121

121

44

44

4

4

26

26

458

458

113

113

7

7

23

23

1

1

25

25

157

157

4

4

142

142

704

669

669

35

35

344

344

263

263

23

23

58

58

1051

631

50

50

50

581

581

581

420

420

420

420

5082

5082

127

127

127

4955

91

91

171

171

740

740

57

57

10

10

1062

1062

54

54

77

77

1

1

1

1

2639

2639

49

49

3

3

214

214

9

9

9

205

205

205

57104
147

187

187

187

187

4169

4169
28

259

259

809

809

168

168

11

11

418

418

44

44

344

344

1050

1050

503

503

439

439

50

50

3

3

43

43

7877

457
1283

331

331

7

7

447

447

41

41

7

7

7

49

49

49

1038

140

140

99

99

799

799

1288

108

108

19

19

9

9

4

4

591

591

252

252

261

261

44

44

53

53

53

168

168

168

69

69

69

2228

1888

1888

340

340

285

285

285

553

178

178

1

1

9

9

161

161

150

150

54

54

622

130

130

5

5

61

61

426

426

234

111

111

25

25

98

98

202

153

146

146

7

7

49

45

45

4

4

4485

206
3439

22

22

5

5

117

117

1456

1456

121

121

24

24

423

423

174

174

820

820

71

71

525

525

525

393

393

393

128

55

55

73

73

5

5

5

5

4

4

4

4

8986

1304

1304

1304

2036

2036

2036

45

45

45

99

99

99

875

8

8

119

119

533

533

86

86

71

71

58

58

1126

1126

1126

861

861

861

1569

99

99

126

126

600

600

740

740

4

4

211

211

211

860

860

860

1945

1945

568

568

1377

1377

289

267

6

6

261

261

22

22

22

1066

1066

366

366

31

31

669

669

2331

1658

76

76

1

1

1581

1581

583

547

547

35

35

1

1

77

77

77

13

13

13

1
7658

1626

826

826

186

186

3

3

98

98

198

198

1

1

15

15

12

12

79

79

167

167

21

21

20

20

4301

2446

2446

621

621

650

650

312

312

2

2

270

270

1730

770

770

533

533

395

395

32

32

468

468

468

468

40

40

40

40

643

616

616

616

27

27

27

948

948

7

7

62

62

63

63

363

363

352

352

101

101

527

377

377

377

41

41

41

109

109

109

1137

1137

234

234

903

903

58

58

58

58

20

20

20

20

153

153

153

153

375

375

16

16

359

359

421

232

39

39

193

193

189

189

189

18

18

18

18

1449

441

430

430

11

11

1008

66

66

254

254

688

688

1506

1506

985

985

521

521

166

166

166

166

486

486

486

486

2642

308

113

113

23

23

172

172

159

27

27

132

132

2175

2175

2175

6696

2647

33

33

619

619

41

41

534

534

301

301

981

981

138

138

49

49

49

2815

1728

1728

2

2

975

975

110

110

1185

829

829

356

356

303

303

30

30

30

30

273

81

81

81

192

190

190

2

2

1922

1922

1922

1922

1464

1464

458

458

44819

52

52

52

52

52

44

44

44

44

44

44723
113

24232
155

11321
15

225

225

460

460

129

129

544

544

66

66

37

37

225

225

1412

1412

45

45

25

25

377

377

85

85

3

3

1451

1451

303

303

3404

3404

265

265

442

442

807

807

1001

1001

12756

1858

1858

46

46

6

6

14

14

221

221

3

3

302

302

2854

2854

1838

1838

10

10

638

638

7

7

733

733

526

526

2349

2349

52

52

179

179

461

461

190

190

469

469

1228

917

859

859

58

58

311

311

311

19142

71

66

66

3

3

2

2

6344

62

62

338

338

232

232

5704

5704

8

8

12727
4

49

49

1494

1494

955

955

2028

2028

1252

1252

10

10

394

394

3

3

528

528

50

50

61

61

14

14

155

155

549

549

5

5

417

417

94

94

5

5

834

834

815

815

176

176

137

137

2588

2588

110

110

8

8

8

8

715

715

715

625

192

192

433

433

90

90

90

851

851

851

851

189

189

67

67

538

538

35

35

2

2

20

20

34220
9

171

171

171

171

171

308

308

308

98

98

210

210

5
31374

66
7633

21

21

21

1601

703

703

876

876

22

22

9

9

9

869

869

869

185

185

185

754

754

754

31

31

31

1092

1092

1092

146

146

146

2398

2398

2398

434

434

434

27

25

25

2

2

23736
1097

2348

675

675

504

504

18

18

442

442

709

709

1037

796

796

129

129

112

112

32

26

26

6

6

9501

772

772

25

25

2064

2064

719

719

965

965

2018

2018

2938

2938

3551

19

19

71

71

190

190

1455

1455

1325

1325

448

448

43

43

48

10

10

36

36

2

2

728

310

310

416

416

2

2

1071

1071

1071

4323

4323

4323

1496

1496

26

26

26

1470

1470

1470

862

862

862

306

306

4

4

544

544

8

8

2787

2787

2787

2787

140

140

50

50

107

107

2105

2105

14

14

300

300

71

71

6937

107
6937

1358

1358

1358

1358

99

99

99

99

391

391

138

138

3

3

250

250

1

1

1

1

2417
38

34
609

241

241

170

170

1

1

163

163

1770
4

84

84

1682

1682

91

91

91

91

2473

393

196

196

9

9

23

23

2

2

24

24

116

116

23

23

111
2080

216

216

1748

1748

5

5

65

8

8

8

8

8

57

57

57

19

19

38

38

36

36

36

36

36

36

899

899

807

807

417

417

390

390

92

92

41

41

33

33

13

13

5

5

824

824

815

815

281

281

493

493

41

41

9

9

9

9

237

237

25

25

25

25

205

205

205

205

7

7

7

7

33

33

33

33

33

33

12590

12274

26

26

26

26

2
12248

3285

25

25

1869

1869

66

66

5

5

91

91

24

24

1205

1205

7

7

7

1262

4

4

7

7

957

957

16

16

278

278

1185

752

752

232

232

201

201

151

151

151

2315

2315

2315

4041

668

668

643

643

2598

2598

132

132

316

316

316

229

229

87

87

898

898

898

898

742

742

156

156

6

6

6

6

6

6

53

53

53

53

53

53

11069

11069

11069

188

62

62

126

126

339

339

339

622

3

3

9

9

6

6

5

5

231

231

1

1

250

250

9

9

108

108

6

6

6

2

2

2

15

15

15

94

94

94

8552

38

38

73

73

9

9

106

106

6

6

8304

8304

16

16

39

11

11

28

28

321

8

8

201

201

45

45

33

33

34

34

891

7

7

884

884

5122

5122

4259

4259

20

20

123

123

697

697

3419

3419

82

82

82

82

781

781

765

765

16

16

162

162

162

162

162

162

33

33

33

33

33

33

316
88273

10049

878

878

878

878

7313

7313
1

1181

1181

235

235

980

980

364

364

585

585

13

13

1199

1199

111

111

68

68

13

13

77

77

1191

1191

20

20

45

45

41

41

92

92

251

251

5

5

841

841

1678

1678

1672

1672

6

6

99

99

99

99

81

81

18

18

63

63

2

2

2

2

2

42602
294

6767

158

158

158

6609

1704

1704

4

4

128

128

3502

3502

47

47

273

273

78

78

246

246

619

619

8

8

600

600

2

2

10

10

19

19

63

63

69

69

329

329

77

77

22

22

9

9

151

151

145

145

6

6

757

757

744

744

13

13

106

106

106

106

18
20317

10

10

10

86

86

86

1248

908

908

340

340

263

263

263

7403
1

169

169

804

804

939

939

1758

1758

165

165

47

47

105

105

121

121

434

434

332

332

131

131

94

94

89

89

1094

1094

3

3

63

63

153

153

880

880

21

21

5
11289

33

33

51

51

467

467

479

479

236

236

1135

1135

1878

1878

29

29

984

984

129

129

1373

1373

1268

1268

83

83

263

263

2

2

2874

2874

13610

43

43

43

13567

58

58

50

50

351

351

15

15

149

149

75

75

14

14

30

30

2183

2183

552

552

1

1

7

7

37

37

41

41

365

365

4

4

443

443

2660

2660

555

555

151

151

662

662

81

81

714

714

81

81

21

21

84

84

1083

1083

383

383

52

52

52

52

2433

2433

180

180

552

206

206

206

206

41

41

41

41

256

256

28

28

209

209

19

19

49

49

49

49

10602

8243
7

2413

41

41

587

587

192

192

144

144

89

89

488

488

5

5

86

86

26

26

109

109

505

505

141

141

1503

345

345

934

934

135

135

89

89

2400

43

43

1939

1939

352

352

18

18

48

48

697

210

210

18

18

312

312

94

94

45

45

18

18

218

70

70

148

148

484

330

330

154

154

521

119

119

402

402

1728

1728

1011

1011

81

81

374

374

69

69

193

193

631

603

603

603

28

28

28

851

91

91

78

78

13

13

90

90

90

90

2

2

1

1

1

1

650

420

54

54

366

366

230

230

230

18

18

18

18

495

495

18

18

18

477

477

477

261

261

261

261

261

6421

6167

6167

1484

1484

4405

4405

240

240

38

38

254

254

254

254

9893

248

248

248

248

7509

57

57

57

2510

2510

2510

935

613

613

194

194

128

128

298

298

298

3247

436

436

44

44

1203

1203

563

563

1001

1001

462

462

462

1906

1906

11

11

664

664

1231

1231

230

230

230

230

23

23

8

7

7

1

1

15

14

14

1

1

5961

1750

629

393

393

37

37

180

180

19

19

1121

328

328

246

246

547

547

1194

1194

98

98

1095

1095

1

1

129

129

129

129

2610

1422

1341

1341

81

81

1188

942

942

26

26

189

189

2

2

14

14

15

15

278

276

90

90

18

18

57

57

111

111

2

2

2

245

245

245

245

245

1602

1602

1602

1602

1569

1569

33

33

210

210

210

210

166

166

44

44

899

899

194

194

194

194

119

119

55

55

64

64

37

37

37

37

20

20

20

20

514

477

118

118

331

331

28

28

37

37

37

15

15

15

15

38

38

38

38

38

38

10

10

10

10

10

10

248

248

248

248

248

248

308

308

9

9

9

9

299

299

299

299

6164

6164

880

880

880

880

2050

428

323

323

105

105

1

1

1

1621

92

92

6

6

335

335

412

412

720

720

56

56

958

237

237

237

721

721

721

60

60

60

60

210

210

210

210

104

53

53

53

51

51

51

1902

1323

1323

1323

8

8

8

27

27

27

165

35

35

12

12

118

118

379

379

379

6351

6351
1

907

44

44

44

863

636

636

116

116

111

111

5443
1

14

4

4

10

10

351

306

306

45

45

3337
68

139

139

82

82

724

724

90

90

522

522

815

815

69

69

507

507

318

318

3

3

1740

88

88

764

764

396

396

255

255

35

35

17

17

185

185

2267

2267

503

212

75

75

137

137

291

291

291

17

17

17

17

103

103

103

103

1308

1171

1171

1171

32

32

32

105

105

105

210

63

1

1

62

62

147

147

147

126

126

126

126

147

147

147

9

9

9

138

73

73

65

65

894

894

325

325

325

325

569

569

83

83

486

486

5600

5600

249

248

16

16

227

227

5

5

1

1

1

5351

5351

86

86

50

50

625

625

4590

4590

744

744

744

674

674

674

70

70

70

12660

12660

197
12660

480

48

48

432

432

4826

4756

4756

70

70

205

205

205

2954

75

75

841

841

60

60

1

1

74

74

1729

1729

35

35

139

139

3998

1882

1882

311

311

1805

1805

779

18

18

18

18

18

575

575

575

575

575

186

186

1

1

1

185

185

185

494

494

494

24

24

24

470

470

470

281

281

281

281

8

8

273

273

2022

2022

2022

500

500

500

1365

327

327

228

228

810

810

25

25

25

132

132

132

85

85

3

3

3

3

82

72

72

72

10

10

10

142

142

142

84

84

84

58

58

58

512

2

2

2

2

2

510

510

510

155

155

355

355

389

389

21

21

21

21

368

368

27

27

341

341

95

95

95

95

95

95

2269

2269

2269

616

81

81

535

535

13

13

13

974

63

63

911

911

306

256

256

50

50

360

301

301

59

59

3534

3534

3534

164

164

164

275

275

275

2147

573

573

1095

1095

23

23

456

456

78

78

78

2

2

2

868

868

868

44

35

26

26

26

26

9

9

9

9

9

9

9

9

9

1101

1101

1101

525

163

163

362

362

576

576

576

278

278

144

144

144

144

134

134

124

124

10

10

78
39030

64
9336

7544

1461

1370

1370

91

91

26

26

26

555

79

79

11

11

5

5

125

125

13

13

316

316

6

6

713

88

88

4

4

368

368

122

122

131

131

589

589

589

1918

662

662

142

142

1106

1106

8

8

6

6

6

851

8

8

16

16

780

780

1

1

46

46

1413

963

963

450

450

12

12

12

2
1622

226

226

226

1291

99

99

33

33

9

9

270

270

48

48

160

160

426

426

243

243

3

3

21

21

21

82

82

82

106

106

106

106

2029

2029

2029
1593

436

436

1430

1430

1430

726

726

704

704

26157
81

3040

1218

1218

1218

638

638

638

1063

1063

1063

103

103

103

6

6

6

12

6

6

6

6

22323

14787

84

84

3

3

460

460

32

32

7355

7355

38

38

43

43

6772

6772

1687

12

12

1616

1616

57

57

2

2

4299

2782

2782

304

304

115

115

1033

1033

65

65

1550

1550

1550

569

569

49

49

520

520

144

144

144

144

5336
683598

1807

5

5

5

5

1715

1310

743

743

3

3

136

136

8

8

260

260

160

160

405

196

196

209

209

87

87

87

87

495

495

383

383

383

112

112

112

2301

225

43

43

43

182

28

28

154

154

1856

1856

90

90

28

28

1738

1738

220

220

220

220

2
6400

2135

2117

1402

1402

714

714

1

1

18

12

12

6

6

3662

2651

360

360

2218

2218

73

73

2

2

2

305

102

102

203

203

639

12

12

294

294

333

333

65

58

58

7

7

580

580

549

549

31

31

21

21

21

21

5909

403

136

136

136

267

131

131

94

94

42

42

690

690

34

34

54

54

418

418

31

31

142

142

11

11

4816

453

453

453

3716
3

99

99

436

436

21

21

4

4

301

301

19

19

32

32

1064

1064

11

11

901

901

21

21

4

4

800

800

647

2

2

622

622

23

23

5043

2426
5

1181

61

61

148

148

211

211

16

16

4

4

46

46

423

423

272

272

10
1240

102

102

11

11

34

34

1083

1083

2617

2617

1315

1315

43

43

1259

1259

327

171

42

42

42

129

129

129

156

156

156

156

22829

586

586

540

540

46

46

649

649

649

649

9

9

9

9

2051

162

162

162

895

21

21

874

874

343
4

52

52

248

248

39

39

623

550

550

73

73

28

28

28

230

230

230

230

4539

4262

33

33

2

2

912

912

929

929

756

756

1630

1630

277

277

277

8548

351

351

351

8196

37

37

803

803

707

707

683

683

370

370

82

82

30

30

108

108

744

744

1877

1877

13

13

95

95

370

370

909

909

1087

1087

2

2

279

279

1

1

1

3
6026

45

45

45

1479

88

88

1391

1391

861
76

22

22

98

98

49

49

598

598

18

18

3638

195

195

3136

3136

299

299

8

8

191

191

191

191

109

109

109

79

79

12

12

18

18

897

130

130

130

130

767

710

3

3

707

707

11

11

11

46

46

46

13078
346697

1446
108783

860

6

6

139

139

37

37

91

91

282

282

49

49

256

256

418

204

204

6

6

11

11

11

11

75

75

111

111

3448

1496

1496

66

66

1844

1844

1

1

41

41

303

193

193

110

110

282

56

56

43

43

183

183

955

218

218

393

393

193

193

151

151

91542
3419

725

725

5

5

145

145

42

42

69

69

320

320

48

48

3158

3158

92

92

8

8

83

83

87

87

254

254

77

77

491

491

520

520

84

84

265

265

1006

1006

86

86

1637

1637

700

700

425

425

100

100

1277

1277

649

649

579

579

405

405

1104

1104

123

123

4738

4738

277

277

16

16

79

79

381

381

139

139

29

29

469

469

1

1

628

628

320

320

1550

1550

67

67

20

20

1333

1333

262

262

532

532

44

44

481

481

10

10

21

21

14

14

5

5

55

55

218

218

244

244

764

764

50

50

91

91

1164

1164

348

348

72

72

3254

3254

44

44

1

1

627

627

1

1

5

5

49

49

1561

1561

144

144

43

43

203

203

13

13

1227

1227

123

123

97

97

305

305

173

173

440

440

709

709

84

84

12

12

266

266

107

107

194

194

55

55

307

307

1029

1029

3912

3912

3

3

1588

1588

43

43

1067

1067

4

4

197

197

58

58

554

554

102

102

58

58

99

99

689

689

35

35

915

915

155

155

652

652

2509

2509

45

45

25

25

1116

1116

216

216

305

305

144

144

68

68

1251

1251

45

45

49

49

1101

1101

342

342

318

318

190

190

25

25

19

19

52

52

294

294

41

41

4

4

1140

1140

36

36

2

2

28

28

716

716

4

4

292

292

151

151

409

409

495

495

14

14

10

10

22

22

667

667

972

972

4

4

12

12

5653

5653

268

268

36

36

5616

5616

17

17

1

1

254

254

3814

3814

101

101

118

118

318

318

274

274

18

18

1013

1013

34

34

7

7

1474

1474

157

157

55

55

46

46

484

484

515

515

557

557

1564

1564

761

761

216

216

196

196

159

159

14

14

95

95

211

211

754

754

1111

325

325

33

33

753

753

3797

109

109

320

320

2279

2279

595

595

70

70

1

1

423

423

14

14

14

1183

10

10

746

746

27

27

374

374

9

9

17

17

629

629

629

2795

136

136

364

364

71

71

1482

1482

84

84

38

38

14

14

99

99

72

72

1

1

41

41

87

87

36

36

270

270

3

3

3

3

13

13

13

13

5906

227

227

227

80

55

55

25

25

5599
28

69

69

1978

1978

59

59

20

20

88

88

122

122

461

461

614

614

6

6

52

52

13

13

199

199

921

921

2

2

100

100

357

357

10

10

47

47

134

134

319

319

15462

424

6

6

3

3

330

330

85

85

15024
734

21

21

12815

12815

27

27

116

116

24

24

19

19

1

1

66

66

352

352

308

308

2

2

98

98

67

67

143

143

228

228

2

2

1

1

14

10

10

4

4

9642
34

76

76

76

700

700

700

619

1

1

618

618

114

73

73

31

31

7

7

3

3

1

1

1

69

1

1

68

68

22
3328

3

3

11

11

59

59

26

26

2

2

29

29

458

458

398

398

455

455

16

16

3

3

93

93

353

353

90

90

12

12

1

1

121

121

232

232

327

327

219

219

298

298

8

8

92

92

272

268

268

4

4

14

14

14

2

2

2

12

12

12

1474

1

1

7

7

178

178

1

1

646

646

380

380

17

17

136

136

108

108

2

2

2

94

94

94

1818

76

76

1520

1520

222

222

922

306

306

4

4

13

13

107

107

151

151

65

65

10

10

161

161

3

3

68

68

34

34

91

5

5

86

86

23

23

23

23

23457
26

39
4385

23

23

2284

2284

83

83

3

3

213

213

613

613

155

155

40

40

116

116

502

502

45

45

269

269

279

276

276

3

3

866

8

8

42

42

43

43

499

499

154

154

33

33

2

2

37

37

48

48

796

71

71

725

725

13090

3171

3171

6

6

75

75

233

233

244

244

134

134

25

25

1

1

166

166

84

84

1067

1067

66

66

157

157

17

17

34

34

4

4

2

2

6

6

20

20

204

204

9

9

96

96

41

41

613

613

6

6

32

32

1049

1049

626

626

423

423

11

11

75

75

1176

1176

6

6

1327

1327

4

4

3

3

5

5

1

1

460

460

143

143

86

86

445

445

18

18

11

11

708

708

1819

1554

1554

31

31

43

43

185

185

1

1

5

5

182

19

19

141

141

18

18

4

4

2

2

2

1990

1097

1097

22

22

19

19

668

668

40

40

62

62

42

42

40

40

22

4

4

13

13

3

3

2

2

992

992

745

745

14

14

164

164

65

65

4

4

398

398

2

2

396

396

5
6528

489

217

217

272

272

197

197

197

594

378

378

76

76

5

5

135

135

4137

662

662

254

254

586

586

75

75

2560

2560

1106

19

19

1061

1061

22

22

4

4

1142

331

298

298

33

33

336

336

336

475

464

464

11

11

5149

50

50

50

2890

359

359

75

75

1570

1570

16

16

870

870

61

61

61

1

1

1

2072

675

675

378

378

296

296

100

100

157

157

118

118

9

9

175

175

164

164

6

6

6

69

30

30

39

39

454

454

454

454

2
6764

2407

80

80

19

19

133

133

332

332

616

616

819

819

408

408

806

3

3

13

13

194

194

137

137

345

345

105

105

9

9

1453

696

696

335

335

175

175

26

26

5

5

173

173

43

43

2096

425

425

512

512

750

750

328

328

81

81

3619

279

163

163

81

81

32

32

3

3

1459

52

52

389

389

127

127

33

33

325

325

25

25

207

207

23

23

248

248

30

30

1657

471

471

1

1

1039

1039

146

146

8

8

8

39

39

39

37

37

37

140

31

31

109

109

36

36

36

36

534
35641

258

258

258

3938

585

585

363

363

179

179

525

525

18

18

249

249

83

83

82

82

142

142

73

73

67

67

63

63

86

86

202

202

34

34

577

577

49

49

19

19

542

542

607

600

600

6

6

1

1

1172

41

41

175

175

32

32

11

11

105

105

808

808

5

5

5

2580

30

30

471

471

66

66

1512

1512

67

67

324

324

110

110

1532

449

449

69

69

11

11

41

41

5

5

369

369

588

588

544

541

541

3

3

69

69

69

318

94

94

8

8

31

31

130

130

22

22

33

33

10628
6

1437

1437

6

6

31

31

60

60

839

839

47

47

24

24

45

45

291

291

27

27

249

249

9

9

891

891

1

1

495

495

261

261

112

112

5

5

69

69

26

26

1006

1006

656

656

227

227

8

8

77

77

562

562

163

163

12

12

56

56

5

5

146

146

2547

2547

192

192

40

40

1755

50

50

2

2

67

67

518

518

7

7

85

85

192

192

30

30

656

656

50

50

79

79

19

19

162

162

162

447

366

366

81

81

2

2

2

45

45

45

15

15

15

50

50

50

18

18

18

122

122

122

5394
161

29

29

99

99

130

130

95

95

17

17

2595

2595

154

154

180

180

216

216

20

20

894

894

669

669

20

20

115

115

1252

161

161

1021

1021

70

70

649

27

27

5

5

62

62

555

555

10

10

10

129

129

129

189

81

81

108

108

3217

1

1

85

85

80

80

302

302

540

540

275

275

98

98

3

3

2

2

17

17

71

71

86

86

5

5

21

21

5

5

631

631

176

176

90

90

221

221

5

5

1

1

41

41

461

461

10

10

10

10

108024
1193

32

29

29

3

3

2769

734

734

3

3

1

1

27

27

1652

1652

11

11

42

42

26

26

87

87

113

113

73

73

3

3

3

286

57

57

228

228

1

1

2281

1

1

339

339

5

5

144

144

7

7

10

10

411

411

106

106

62

62

1168

1168

11

11

17

17

111

93

93

18

18

246
26998

60

60

17

17

79

79

45

45

63

63

164

164

339

339

58

58

687

687

60

60

42

42

992

992

1731

1731

900

900

34

34

208

208

153

153

1557

1557

165

165

43

43

102

102

55

55

722

722

104

104

431

431

343

343

113

113

1696

1696

24

24

68

68

48

48

82

82

39

39

79

79

973

973

4561

4561

40

40

1427

1427

10

10

14

14

78

78

905

905

2

2

7

7

27

27

35

35

1503

1503

176

176

392

392

52

52

58

58

88

88

4

4

355

355

1943

1943

5

5

225

225

526

526

1188

1188

205

205

249

249

401

401

8118
15

370

370

83

83

36

36

27

27

207

207

5

5

902

902

9

9

6

6

44

44

62

62

83

83

20

20

4

4

63

63

223

223

93

93

35

35

2

2

906

906

409

409

14

14

856

856

482

482

442

442

386

386

71

71

34

34

20

20

13

13

8

8

280

280

234

234

89

89

1440

1440

13

13

98

98

34

34

958
33537

257

257

754

754

100

100

1506

1506

595

595

1088

1088

28

28

18

18

325

325

16

16

19

19

22

22

89

89

544

544

155

155

2010

2010

2991

2991

56

56

1388

1388

85

85

420

420

1940

1940

597

597

443

443

167

167

49

49

1464

1464

32

32

618

618

126

126

2208

2208

85

85

8

8

173

173

2205

2205

527

527

1119

1119

1537

1537

307

307

442

442

232

232

9

9

44

44

2003

2003

1760

1760

1675

1675

11

11

321

321

11

11

32090
177

29

29

77

77

4

4

498

498

218

218

23

23

10

10

87

87

97

97

194

194

342

342

39

39

1581

1581

179

179

6

6

4190

4190

87

87

28

28

13

13

26

26

13

13

3

3

2598

2598

157

157

59

59

304

304

4

4

14

14

369

369

3

3

141

141

132

132

84

84

170

170

12

12

15

15

4298

4298

53

53

65

65

198

198

1896

1896

591

591

373

373

127

127

915

915

572

572

162

162

127

127

102

102

305

305

694

694

23

23

9

9

47

47

40

40

1243

1243

1016

1016

365

365

525

525

14

14

1

1

15

15

4

4

74

74

23

23

111

111

60

60

1215

1215

133

133

805

805

272

272

219

219

22

22

1040

1040

2353

2353

26

26

26

44

6

6

31

31

7

7

316

316

316

220

1

1

219

219

172

172

172

172

418

418

418

418

928

333

11

11

322

322

410

410

410

185

185

185

55

41

10

10

12

12

19

19

14

14

14

2

2

2

2

2

22
9710

359

359

359

359

1791

1791

49

49

34

34

114

114

12

12

1289

1289

31

31

262

262

629

629

3

3

12

12

614

614

6909

4

4

4

4892

3

3

38

38

2088

2088

161

161

248

248

28

28

17

17

1572

1572

400

400

44

44

260

260

33

33

357

1

1

301

301

55

55

1656

8

8

404

404

41

41

1

1

38

38

376

376

43

43

710

710

35

35

64

64

64

58

58

6

6

3033

2105

636

73

73

167

167

27

27

369

369

1344

13

13

310

310

194

194

827

827

124

124

124

1

1

1

928

407

189

189

218

218

303

77

77

35

35

177

177

14

14

218

1

1

8

8

2

2

207

207

1062

938

912

287

287

407

407

218

218

26

26

26

107

84

16

16

68

68

23

23

23

17

17

17

17

1049

1049

1039

1039

1039

10

10

10

32

32

32

32

32

231307
14473

11922

2605
2

252

252

314

314

5

5

187

187

13

13

88

88

882

882

58

58

1

1

157

157

23

23

278

278

303

303

42

42

11
9317

136

136

75

75

23

23

380

380

32

32

11

11

33

33

24

24

11

11

64

64

13

13

142

142

16

16

2747

2747

1126

1126

15

15

5

5

29

29

22

22

1

1

28

28

5

5

39

39

1244

1244

97

97

31

31

14

14

22

22

31

31

84

84

253

253

15

15

608

608

40

40

102

102

41

41

115

115

707

707

419

419

280

280

226

226

924

448

55

55

2

2

391

391

476

443

443

33

33

1

1

1

1

2345

939

302

302

38

38

9

9

39

39

46

46

74

74

8

8

345

345

78

78

1406

21

21

28

28

504

504

125

125

289

289

97

97

86

86

158

158

98

98

36

36

36

36

2
2708

68

27

27

15

15

4

4

10

10

12

12

32

32

32

1665

5

5

20

20

118

118

14

14

433

433

26

26

599

599

395

395

55

55

941

291

291

600

600

3

3

34

34

13

13

2858

2858

2753

2753

7

7

98

98

4

4

4

4

4357

106

8

8

98

98

1154

24

24

180

180

1

1

249

249

694

694

6

6

2

2

2

222

187

187

10

10

25

25

154

154

154

2719
70

9

9

1

1

110

110

179

179

58

58

393

393

113

113

81

81

4

4

113

113

236

236

317

317

180

180

5

5

53

53

23

23

502

502

272

272

1295
11

30
1214

88

88

47

47

195

195

104

104

22

22

55

55

5

5

7

7

58

58

12

12

141

141

13

13

60

60

149

149

5

5

31

31

187

187

5

5

70

38

38

32

32

5
6873

22

22

22

744

744

744

41
1931

251

251

732

732

896

896

11

11

4171
6

3736

3736

429

429

17192

336
17192

12

12

829

829

439

439

33

33

95

95

16

16

19

19

233

233

334

334

329

329

32

32

15

15

171

171

114

114

27

27

25

25

1

1

302

302

199

199

384

384

8

8

9

9

19

19

95

95

8

8

3

3

2

2

828

828

51

51

17

17

8

8

13

13

195

195

152

152

116

116

715

715

40

40

20

20

633

633

53

53

263

263

4

4

54

54

20

20

102

102

9

9

49

49

730

730

63

63

986

986

109

109

835

835

110

110

435

435

95

95

16

16

33

33

19

19

56

56

179

179

40

40

5

5

34

34

56

56

36

36

12

12

1

1

264

264

342

342

15

15

59

59

1460

1460

3603

3603

60

60

80

80

14

14

14

14

49

8

8

8

39

39

39

2

2

2

46

46

46

46

1127

1127

1068

1068

12

12

47

47

481

481

406

406

55

55

20

20

26485

397

213

213

184

184

430
26088

258

258

45

45

622

622

39

39

9

9

8

8

640

640

6

6

604

604

14

14

53

53

14

14

2103

2103

41

41

274

274

20775

20775

153

153

16580

12

12

12

518

3

3

127

127

36

36

30

30

39

39

283

283

9606
203

1839

1839

118

118

7202

7202

3

3

29

29

59

59

153

153

1

1

1

354

209

209

24

24

121

121

37

15

15

22

22

30

30

30

6022
10

35

35

5321

5321

1

1

53

53

31

31

195

195

376

376

1829

1133

2

2

1131

1131

696

696

696

53234
201

4671

12

12

505

505

423

423

2965

2965

7

7

95

95

326

326

5

5

41

41

289

289

3

3

103

103

103

13577
76

11925

11925

234

234

1325

1325

2

2

15

15

5668
237

36

36

496

496

4899

4899

676

4

4

672

672

4324

135

135

152

152

293

293

966

966

5

5

205

205

45

45

1105

1105

147

147

820

820

410

410

1

1

8

8

32

32

22530
370

2140

2140

9

9

1993

1993

753

753

2780

2780

137

137

404

404

773

773

100

100

96

96

161

161

1925

1925

2214

2214

5

5

76

76

105

105

1831

1831

610

610

6

6

40

40

2951

2951

1015

1015

445

445

413

413

27

27

15

15

115

115

59

59

640

640

226

226

96

96

7
1484

1477

1477

4022

2069

242

242

20

20

1608

1608

44

44

11

11

144

144

1873

61

61

54

54

5

5

39

39

263

263

383

383

514

514

287

287

56

56

211

211

80

80

80

1

1

1

1

667

3
667

664

664

179
61798

14

14

14

3352

105

105

1246

1246

1944

1944

1

1

56

56

594

594

594

8442
79

476

476

2272

2272

19

19

3143

3143

748

748

25

25

309

309

161

161

1210

1210

13624
3

89

89

1423

1423

8

8

341

341

21

21

22

22

15

15

270

270

29

29

51

51

1235

1235

617

617

107

107

1149

1149

137

137

137

137

101

101

77

77

7612

7612

28

28

152

152

1273

474

474

7

7

183

183

58

58

551

551

34
10281

1921

1921

656

656

255

255

75

75

1

1

38

38

1947

1947

42

42

31

31

69

69

65

65

249

249

2033

2033

10

10

369

369

2459

2459

27

27

619

619

619

1222

1222

1222

24

24

24

516

8

8

31

31

17

17

20

20

8

8

394

394

38

38

74
9575

229

229

930

930

213

213

69

69

56

56

202

202

2683

2683

83

83

430

430

80

80

178

178

9

9

41

41

955

955

13

13

1949

1949

42

42

120

120

1219

1219

1066

1066

1066

420

11

11

5

5

246

246

158

158

230

230

230

5

5

5

2911

549

549

2362

2362

2403

292

292

49

49

58

58

18

18

1905

1905

8

8

22

22

33

33

3

3

15

15

33

33

33

158

158

158

795

6

6

334

334

181

181

66

66

16

16

6

6

3

3

39

39

58

58

86

86

1476

934

934

64

64

478

478

1298
2

1015

1015

67

67

129

129

26

26

32

32

27

27

1288

26

26

64

64

142

142

979

979

77

77

2079

24

24

24

24

2055

296

58

58

238

238

541

79

79

24

24

109

109

329

329

11

8

8

3

3

1207

110

110

2

2

32

32

637

637

6

6

380

380

40

40

1697

925

897

622

622

13

13

101

101

2

2

22

22

137

137

28

28

28

12

12

12

12

722

722

501

501

50

50

171

171

38

38

38

38

483

483

468

468

468

15

15

15

1632

1632

1632

1414

1414

218

218

4295

273

273

273

273

226

226

226

226

184

184

184

184

34

34

34

34

32

32

19

19

13

13

228

228

228

228

2676

2676

12

12

731

731

48

48

123

123

1762

1762

459

459

46

46

413

413

20

20

20

20

163

163

163

163

4
7469

1721

1714

210

210

104

104

1213

1213

20

20

167

167

7

7

7

223

223

223

223

14

14

14

14

169

94

67

67

27

27

75

75

75

360

10

10

10

323

173

173

36

36

114

114

27

27

27

4978

95

95

95

75

75

75

4267

23

23

7

7

8

8

60

60

3079

3079

181

181

80

80

774

774

14

14

41

41

524

44

44

67

67

27

27

361

361

25

25

17

17

17

180

180

180

180

180

2458

56

56

56

56

186

101

101

101

85

85

85

2216

1826

1798

1798

28

28

390

22

22

368

368

647

78

78

78

78

569

25

25

25

544

465

465

27

27

52

52

268

268

122

101

101

21

21

146

114

114

32

32

6845

61

61

61

61

394

8

8

8

189

141

141

5

5

43

43

197

2

2

105

105

90

90

6390

3059

1223

1223

201

201

1485

1485

70

70

65

65

15

15

25

25

25

81

81

81

1107

31

31

44

44

342

342

150

150

1

1

539

539

437

115

115

288

288

34

34

1445

300

300

252

252

893

893

10

10

10

226

133

133

83

83

10

10

3144

1373

1266

1266

1266

107

107

107

1771

1361

861

861

327

327

3

3

133

133

37

37

410

2

2

13

13

17

17

320

320

58

58

1474

1474

1474

164

164

1310

1310

1946
1

213

213

128

128

61

61

24

24

64

9

9

9

55

7

7

48

48

46

46

41

41

5

5

657

657

657

657

693

20

20

20

615

208

208

314

314

5

5

88

88

58

1

1

57

57

272

272

272

272

4

4

4

4

4

3617

564

66

66

66

163

163

163

335

80

80

48

48

2

2

205

205

2745

2745

2406

2406

339

339

200

79

79

79

121

53

53

68

68

108

108

104

104

4

4

951

951

254

22

22

232

232

697

3

3

190

190

351

351

153

153

113

113

113

10

10

10

1

1

1

66

3

3

63

63

36

36

36

235

235

235

235

201

201

34

34

10564

10564

1524

1524

8

8

1435

1435

81

81

2801

1824

14

14

70

70

1319

1319

14

14

6

6

20

20

381

381

977

352

352

625

625

6239

6239
72

66

66

16

16

23

23

628

628

169

169

1160

1160

117

117

254

254

2070

2070

1115

1115

102

102

73

73

1

1

121

121

252

252

175

175

175

175

175

175

87

87

87

87

87

87

293

293

293

293

130

130

163

163

16007

246

246

246

246

246

992

992

40

40

40

952

348

348

604

604

2652

1448

376

359

359

17

17

114

114

114

958

320

320

2

2

413

413

223

223

592

592

592

592

87

87

82

82

5

5

525

307

307

307

218

218

218

12117

76

51

30

30

21

21

25

25

25

3634

486

82

82

23

23

145

145

236

236

3148

34

34

2407

2407

511

511

196

196

6453

3751

2682

2682

262

262

306

306

501

501

2362

44

44

109

109

13

13

383

383

1132

1132

95

95

579

579

7

7

340

340

340

13

13

13

13

1332

1332

1332

1332

609

609

36

36

65

65

508

508

7659

7659

71

1

1

1

70

70

70

69

69

69

69

588

363

5

5

358

358

225

84

84

141

141

3008

354

225

225

129

129

776

776

776

1878

1

1

1877

1877

1368

891

891

891

69

69

69

109

109

109

299

299

299

2555

2555

2458

2458

16

16

81

81

173

173

173

40

40

40

8

8

8

106

106

106

19

19

19

18836

411

365

330

330

330

35

35

35

46

46

46

46

955

955

917

165

165

227

227

435

435

76

76

14

14

38

32

32

6

6

446

176

176

176

176

270

206

206

206

64

64

64

228

228

228

200

200

28

28

9811

9811

277

9

9

11

11

257

257

2730

524

524

1066

1066

1044

1044

96

96

6804

1024

1024

631

631

10

10

778

778

1295

1295

2

2

1544

1544

1422

1422

98

98

763

763

763

763

763

303

303

104

104

104

199

199

199

1194

1194

964

7

7

344

344

475

475

56

56

1

1

51

51

30

30

230

56

56

8

8

166

166

692

356

356

348

348

8

8

336

336

335

335

1

1

3200

123

123

123

123

3075

205

205

205

2870

2498

2498

22

22

54

54

296

296

2

2

2

2

661

167

167

167

167

494

62

62

62

152

152

152

88

57

57

31

31

39

39

39

105

105

105

48

48

48

172

172

140

140

140

32

32

32

1106
220556

24566
8

3790

3784

1052

1052

7

7

232

232

1881

1881

612

612

6

6

6

2322

193

193

193

149

149

149

424

365

365

59

59

1556

33

33

1425

1425

98

98

2826

163

163

163

2663

1

1

567

567

363

363

1732

1732

34

34

34

34

2482

565

271

271

292

292

2

2

1638

1638

1638

266

266

266

13

13

13

258

94

94

94

164

164

164

707

601

601

601

106

106

106

341

128

128

128

213

213

213

9463

527

2

2

107

107

418

418

756
8395

659

659

1811

1811

2399

2399

270

270

49

49

1257

1257

18

18

351

351

819

819

6

6

541

2

2

284

284

255

255

542

174

174

174

368

368

368

1793

12

12

12

8

8

8

10

10

10

240

240

240

173

173

173

118

118

118

340

340

340

444

440

440

4

4

448

448

448

183524
1749

38684
909

122

75

75

47

47

770

108

108

662

662

1086

1086

1086

460

460

460

9367

1619

1619

68

68

142

142

326

326

7212

7212

15

2

2

13

13

3223

3223

3223

6408

1378

1378

175

175

130

130

492

492

2521

2521

1009

1009

83

83

620

620

3

3

3

158

88

88

70

70

17

17

17

392

392

392

682

682

682

511

125

125

67

67

89

89

230

230

1346

1346

1346

51

2

2

49

49

759

477

477

247

247

35

35

167

167

167

94

94

94

623

623

623

276

203

203

73

73

35

35

35

198

198

198

52

16

16

36

36

43

43

43

1

1

1

185

185

185

1139

107

107

266

266

580

580

3

3

1

1

182

182

9171

265

265

7

7

635

635

8264

8264

421

121

121

204

204

12

12

84

84

146

146

146

146

77925
158

267

267

267

8873

8873

8873

41

8

8

33

33

387

131

131

256

256

9372

1124

1124

72

72

8176

8176

31084
1750

375

375

47

47

1827

1827

682

682

1071

1071

26

26

367

367

1081

1081

816

816

115

115

279

279

106

106

37

37

16678

16678

1004

1004

108

108

7

7

621

621

93

93

35

35

14

14

24

24

1155

1155

794

794

25

25

551

551

94

94

149

149

641

641

419

419

93

93

1

1

1

1015

86

86

408

408

521

521

468

468

468

137

137

137

11973

233

233

788

788

10952

10952

160

160

160

1209

38

38

671

671

500

500

10174

1563

1563

37

37

7030

7030

1

1

507

507

595

595

61

61

127

127

253

253

48

17

17

27

27

4

4

334

334

334

3

3

3

2

2

2

2219

125

125

327

327

1702

1702

65

65

17786
18

3

3

3

2670

204

204

2392

2392

74

74

1
10904

170

170

1144

1144

522

522

7

7

735

735

1163

1163

25

25

1764

1764

5131

5131

242

242

232

232

232

2003

173

173

204

204

166

166

321

321

1042

1042

97

97

1956

1953

1953

3

3

4209

1395

882

882

202

202

311

311

82

82

82

1110

1110

1110

1072

410

410

358

358

304

304

550

129

129

421

421

146
41814

1848

1

1

1847

1847

10430

26

26

1

1

67

67

3891

3891

98

98

5202

5202

1043

1043

102

102

8156

2633

2633

22

22

387

387

223

223

4725

4725

166

166

20616
48

760

760

171

171

5837

5837

1233

1233

1683

1683

356

356

6059

6059

825

825

1811

1811

1833

1833

448

448

448

170

137

137

32

32

1

1

1211

940

151

151

6

6

4

4

47

47

440

440

292

292

271

271

271

11360

5783

205

4

4

201

201

186

186

186

280

280

280

46

46

46

2359

89

89

1265

1265

1005

1005

2557

15

15

682

682

46

46

199

199

195

195

860

860

114

114

446

446

150

150

150

367

355

355

355

12

12

12

100

100

100

100

5110

2243

200

200

80

80

9

9

462

462

80

80

2

2

1162

1162

248

248

998

698

698

11

11

289

289

50

50

50

53

53

53

1282

1262

1262

20

20

3

3

3

481

167

167

314

314

809

809

567

567

137

137

71

71

167

167

20

20

172

172

242

242

242

242

538

538

538

410

285

285

34

34

91

91

128

128

128

5709

5696

5591

4345

38

38

243

243

349

349

3715

3715

1169

437

437

343

343

389

389

77

77

77

1

1

1

1

104

104

104

104

13

13

13

13

13

6137

6137

6137

4982

173

173

315

315

99

99

20

20

4375

4375

1082

1

1

560

560

2

2

130

130

3

3

381

381

3

3

2

2

73

73

73

20539

16942
2

1052

508

41

41

215

215

252

252

25

25

25

487

291

291

66

66

115

115

2

2

13

13

4

4

4

28

8

8

20

20

37

37

14

14

23

23

47

47

1

1

46

46

122

122

55

55

3

3

57

57

7

7

179

179

179

179

437

437

255

255

182

182

233

224

9

9

23

23

192

192

9

9

9

4266

28

28

28

3464

164

164

84

84

31

31

3

3

1

1

358

358

51

51

31

31

9

9

872

872

167

167

635

635

108

108

567

567

383

383

774

42

42

732

732

479

479

479

479

37

37

37

37

72

72

72

72

96

96

96

96

106

106

106

106

180

180

156

156

24

24

72

72

72

72

2739

2716

6

6

231

231

2479

2479

23

23

23

371

371

371

371

6009

1533

8

8

565

565

78

78

20

20

57

57

729

729

76

76

4476

105

105

82

82

698

698

3575

3575

16

16

406

406

69

69

337

337

2830

9
2830

1266

103

103

717

717

2

2

444

444

8

8

8

118

118

118

39

39

39

1177

2

2

665

665

510

510

213

213

213

767

767

767

424

424

343

343

1328

1328

31

31

31

31

62

62

62

62

282

282

22

22

260

260

953

953

161

161

331

331

21

21

344

344

10

10

53

53

9

9

24

24

13109

13109

4

4

4

4

95

86

86

86

9

9

9

13010

2

2

2

2679

34

34

3

3

92

92

4

4

105

105

24

24

89

89

257

257

51

51

2

2

30

30

145

145

77

77

8

8

352

352

32

32

8

8

14

14

4

4

347

347

976

976

25

25

2822

72

72

195

195

48

48

279

279

112

112

86

86

1601

1601

172

172

108

108

8

8

141

141

932

707

707

147

147

7

7

71

71

66

66

66

1651

67

67

1578

1578

6

6

76

1

1

4

4

71

71

4

4

4

13

13

13

28

4

4

12

12

10

10

2

2

69

69

69

5
459

423

423

31

31

40

40

40

16

16

16

3613

113

113

194

194

31

31

8

8

405

405

67

67

822

822

11

11

393

393

14

14

7

7

161

161

904

904

224

224

10

10

120

120

114

114

15

15

83

83

83

457

9

9

216

216

178

178

54

54

180

180

180

56

56

56

124

124

124

3142

3142

107

62

62

62

45

45

45

520

514

1

1

19

19

14

14

68

68

412

412

6

6

6

2515

53
2515

118

118

124

124

567

567

722

722

38

38

23

23

20

20

268

268

300

300

282

282

3957
186488

183

183

183

183

183

756

756

756

4

4

23

23

729

729

123

123

123

123

123

18

18

18

18

18

1731

286

116

79

79

37

37

28

28

28

142

142

142

192

192

192

192

67

67

67

67

217

101

1

1

100

100

105

105

105

11

11

11

70

70

70

70

226

226

226

226

24

1

1

1

23

1

1

22

22

570

570

558

558

12

12

79

10

10

10

69

1

1

68

68

1180

27

27

27

27

1062

504

504

504

558

29

29

68

68

16

16

26

26

15

15

5

5

1

1

46

46

135

135

2

2

48

48

3

3

9

9

93

93

54

54

8

8

25

14

14

14

11

8

8

3

3

4

4

4

4

62

62

62

62

344

344

220

108

108

112

112

124

73

73

44

44

7

7

9271

85

85

64

64

21

21

483

483

6

6

331

331

146

146

1096

1096

22

22

117

117

552

552

10

10

73

73

5

5

22

22

81

81

63

63

151

151

9

9

9

9

25

25

25

25

139

139

139

139

2005

1

1

1

5

3

3

2

2

329

278

278

51

51

61

61

61

8

8

8

301

301

301

3

3

3

108

2

2

68

68

38

38

434

118

118

101

101

61

61

33

33

11

11

95

95

15

15

114

92

92

4

4

18

18

619

619

619

22

2

2

20

20

18

18

18

18

3933

3933

15

15

850

850

419

419

730

730

6

6

6

6

302

302

41

41

461

461

22

22

197

197

214

214

73

73

75

75

432

432

69

69

21

21

1478

1044

104

104

16

16

182

182

19

19

606

606

117

117

434

71

71

16

16

7

7

3

3

42

42

233

233

62

62

674

674

448

327

327

121

121

226

3

3

2

2

221

221

21
20778

1024

1024

1

1

59

59

627

627

32

32

56

56

163

163

6

6

70

70

10

10

441

176

7

7

169

169

265

3

3

94

94

123

123

4

4

16

16

25

25

16

16

16

16

20

20

20

20

2290

2290

481

481

150

150

1194

1194

14

14

44

44

9

9

51

51

115

115

48

48

40

40

144

144

2417

2329

25

25

39

39

1

1

70

70

2

2

559

559

416

416

148

148

406

406

2

2

252

252

25

25

332

332

34

34

18

18

88

34

34

28

28

26

26

2

2

2

2

6016
27

21

10

10

11

11

395

393

393

2

2

1554
114

5

5

171

171

364

364

94

94

4

4

2

2

176

176

101

101

25

25

69

69

34

34

39

39

146

146

3

3

207

207

246

17

17

43

43

186

186

843

843

843

1887

187

187

61

61

1639

1639

10

10

10

408

7

7

17

17

358

358

24

24

2

2

511

511

511

101

101

101

13

3

3

10

10

117

1

1

1

116

105

105

11

11

862

862

144

144

11

11

268

268

1

1

350

350

8

8

11

11

69

69

17

6

6

6

11

11

11

1096

1096

2

2

13

13

36

36

108

108

160

160

91

91

686

686

4143

686

43

43

57

57

205

205

171

171

207

207

3

3

2212

1911

1911

218

218

82

82

1

1

25

25

25

1220

48

48

187

187

21

21

140

140

2

2

55

55

107

107

29

29

80

80

67

67

51

51

108

108

3

3

7

7

124

124

7

7

11

11

62

62

12

12

43

43

23

23

33

33

38

38

6

6

30

30

2

2

2258

106

39

39

67

67

1992

147

147

59

59

98

98

10

10

21

21

241

241

203

203

36

36

178

178

665

665

4

4

43

43

287

287

160

10

10

21

21

16

16

69

69

39

39

5

5

223

223

208

208

208

15

15

15

125

23

4

4

4

15

15

15

4

4

4

98

98

98

98

4

4

4

4

18076
3

29
14744

136

136

136

390

8

8

382

382

34

34

34

23

23

23

6

6

6

406

406

406

18

18

18

45

45

45

182

10

10

83

83

13

13

22

22

36

36

18

18

2586

1

1

2585

2585

6695
143

2

2

54

54

50

50

493

493

121

121

1109

1109

5

5

165

165

87

87

5

5

1

1

695

695

420

420

251

251

464

464

9

9

24

24

1132

1132

43

43

49

49

15

15

445

445

2

2

442

442

4

4

279

279

159

159

27

27

403

403

403

8

8

8

3783

303

303

1091

1091

67

67

14

14

743

743

22

22

409

409

93

93

70

70

6

6

965

965

3088

30

14

14

16

16

3058

2914

2914

144

144

241

87

87

87

154

127

127

27

27

3137

2994

117

1

1

116

116

253
2874

12

12

431

431

370

370

358

358

231

231

45

45

8

8

373

373

42

42

751

751

3

3

3

143

143

72

72

71

71

25

25

25

6

6

19

19

836

836

389

389

389

154

154

154

22

20

20

2

2

59

4

4

55

55

93

93

93

119

101

101

18

18

13

13

13

13

13

1633
102082

62033
2474

135

135

135

3184

3184

3184

458

458

458

234

221

221

13

13

4736

34

34

350

350

4334

4334

18

18

106

106

106

354

331

331

23

23

166

160

160

6

6

5025
9

2733

2733

11

11

67

67

2

2

62

62

227

227

234

234

16

16

43

43

200

200

10

10

1312

1312

1

1

98

98

6

6

6

1078

1

1

112

112

219

219

419

419

327

327

371

20

20

107

107

244

244

3

3

3

99
14449

624

624

247

247

3

3

155

155

4495

4495

12

12

176

176

8382

8382

85

85

112

112

59

59

186

185

185

1

1

12
23028

444

444

23

23

69

69

1013

1013

168

168

213

213

31

31

274

274

12

12

2

2

7447

7447

1207

1207

101

101

630

630

1223

1223

7628

7628

2531

2531

26

26

26

21

21

21

2

2

2

62

62

62

133

133

133

482

458

458

24

24

520

46

46

99

99

72

72

60

60

130

130

2

2

111

111

31

31

31

1989

182

182

1807

1807

6

6

6

54

54

54

487

487

487

12

12

12

13

6

6

7

7

23

23

23

43

43

43

1622

150

150

17

17

89

89

420

420

27

27

105

105

35

35

16

16

38

38

50

50

615

615

21

21

5

5

34

34

83

83

83

431

12

12

419

419

2153

2057

23

23

6

6

2028

2028

96

51

51

13

13

18

18

14

14

1473

1199

32

32

48

48

695

695

45

45

153

153

31

31

98

98

12

12

4

4

2

2

79

79

185

21

21

4

4

96

96

64

64

89

10

10

79

79

347

183

83

83

47

47

53

53

164

164

164

12

12

12

12

1

1

1

1

219

49

49

49

170

90

90

80

80

556

556

556

556

8964

911

2

2

27

27

50

50

218

218

389

389

212

212

13

13

320

93

93

227

227

15

15

15

306

306

306

4247

356

356

1114

1114

1636

1636

341

341

10

10

36

36

1

1

14

14

202

202

14

14

427

427

39

39

57

57

1983

19

19

53

53

1911

1911

100

100

100

843

562

562

203

203

78

78

4

4

4

46

36

36

10

10

184

184

184

5

2

2

3

3

1192

283

239

239

7

7

7

7

6

6

24

24

759

340

340

88

88

331

331

150

150

150

537

537

333

333

204

204

13
7233

320

6

6

23

23

291

291

287

287

287

4

4

4

354

344

344

10

10

1933

3

3

66

66

22

22

5

5

19

19

1

1

1224

1224

593

593

628

11

11

68

68

21

21

15

15

52

52

461

461

117

117

117

925

188

188

4

4

733

733

109

109

109

285

206

206

2

2

77

77

31

31

31

66

66

66

8

8

8

271

62

62

56

56

153

153

96

96

96

831

143

143

85

85

1

1

370

370

23

23

3

3

36

36

55

55

115

115

955

2

2

6

6

84

84

679

679

182

182

2

2

27

27

27

27

372

15

15

15

64

32

32

32

32

1

1

1

143

39

39

26

26

63

63

15

15

149

104

104

45

45

31

25

24

24

1

1

6

6

6

120

120

120

120

1613

20

20

20

417

417

417

27

27

27

443

21

21

422

422

2

2

2

704

476

476

2

2

4

4

222

222

14

14

14

14

13552
20

8

8

8

199

53

53

146

146

42

42

42

246

246

246

73

73

73

231

231

231

2442

28

28

238

238

371

371

929

929

264

264

612

612

9

9

9

513

50

50

1

1

1

1

289

289

52

52

120

120

51

51

51

7

7

7

1274

28

28

4

4

809

809

73

73

35

35

89

89

236

236

141

51

51

90

90

103

103

103

232

232

232

3228

207

207

47

47

6

6

17

17

236

236

27

27

38

38

2267

2267

383

383

34

34

34

7

7

7

47

44

44

3

3

353

353

353

1170

1170

1170

152

152

152

1816

69

69

750

750

66

66

931

931

32

32

32

1108

245

245

42

42

820

820

1

1

14

14

14

22936
86

58

58

58

58

105

2

2

2

10

10

10

20

20

20

72

72

72

1

1

1

43

43

43

43

190

140

1

1

40

40

64

64

35

35

50

30

30

20

20

29

29

24

24

5

5

507

48

1

1

47

47

57

57

57

402

89

89

313

313

188

188

188

188

759

62

62

62

430

266

266

10

10

60

60

19

19

39

39

36

36

213

34

34

179

179

54

31

31

23

23

3453

44

44

44

1067

4

4

148

148

3

3

39

39

360

360

7

7

374

374

132

132

111

111

111

2045

49

49

373

373

5

5

40

40

20

20

129

129

1

1

170

170

170

170

18

18

26

26

14

14

736

736

139

139

12

12

100

100

43

43

8

8

8

174

63

63

111

111

4

4

4

137

137

19

19

1

1

42

42

40

40

35

35

210

210

210

210

1726

195

10

10

48

48

129

129

8

8

246

118

118

29

29

3

3

8

8

88

88

193

114

114

79

79

208

208

208

884

884

884

168

168

97

97

71

71

31

31

31

31

133

103

15

15

26

26

62

62

11

7

7

4

4

19

19

19

7486

1419

1320

1320

87

87

12

12

1301

953

953

242

242

102

102

4

4

33

33

33

454

17

17

437

437

2045

73

73

32

32

5

5

424

424

297

297

299

299

101

101

56

56

29

29

214

214

513

513

2

2

118

118

118

47

47

47

1778

114

114

1155

1155

19

19

446

446

3

3

17

17

24

24

52

52

52

239

55

55

21

21

163

163

80

80

80

80

1013

1013

292

292

31

31

250

250

440

440

5086

16

16

16

1002

4

4

195

195

786

786

7

7

10

10

2008

40

40

165

165

118

118

611

611

181

181

24

24

122

122

474

474

48

48

131

131

83

83

11

11

7

7

7

19

19

19

89

89

89

253

54

54

8

8

179

179

12

12

800

565

565

235

235

355

4

4

351

351

138

138

138

399

74

74

4

4

47

47

274

274

1074

1074

1074

1074

71

71

47

47

24

24

8

8

8

8

66

66

66

66

208

208

208

208

3

3

3

3

18

18

18

18

20

20

20

20

20

770091
13043

95
86049

61

61

61

61

2544

725

1

1

371

371

168

168

185

185

591

367

367

224

224

36

36

36

1192

153

153

649

649

124

124

236

236

30

30

10103

10103

10103

10103

68304
320

4
21580

4848

4848

1419

1419

7

7

7

7

1310

1310

10

10

689

689

215

215

3886

3886

562

562

136

136

10

10

88

88

1455

1455

2365

2365

3091

3091

13

13

488

488

977

977

115

115

115

307

294

294

13

13

278

2

2

202

202

60

60

14

14

486

54

54

51

51

278

278

103

103

120

120

120

41

41

41

52

52

52

3161

3159

3159

2

2

1838

318

318

88

88

395

395

181

181

15

15

127

127

3

3

152

152

559

559

9233

645

645

1643

1643

3261

3261

2252

2252

1432

1432

5864

16

16

259

259

1358

1358

103

103

30

30

593

593

1060

1060

2331

2331

114

114

536

518

518

18

18

642

292

292

350

350

10

10

10

94

94

94

278

62

62

162

162

54

54

424

9

9

415

415

241

160

160

81

81

886

64

64

822

822

3503

2011

2011

1492

1492

2720

19

19

831

831

347

347

35

35

148

148

1340

1340

712

41

41

4

4

33

33

634

634

13259

131

131

8556

8556

61

61

1463

1463

501

501

6

6

130

130

163

163

307

307

1421

1421

520

520

347

5

5

96

96

246

246

914

82

82

34

34

2

2

665

665

131

131

26

26

26

171

28

28

143

143

122

32

32

90

90

24

24

24

4918

970

285

285

685

685

250

17

17

115

115

118

118

869

869

869

156

148

148

8

8

216

18

18

79

79

79

79

21

21

19

19

1059

74

74

985

985

1398

140

140

83

83

1057

1057

25

25

14

14

3

3

47

47

18

18

11

11

24

24

24

24

59948
13

51384
972

725

56

56

65

65

600

600

4

4

3

3

3

61

5

5

56

56

29
10007

8

8

316

316

405

405

9

9

39

39

10

10

334

334

142

142

2537

2537

180

180

225

225

43

43

142

142

481

481

52

52

172

172

18

18

207

207

4

4

53

53

3465

3465

2

2

602

602

6

6

128

128

7

7

12

12

23

23

159

159

170

170

27

27

90
29196

28906

28906

2

2

6

6

11

11

181

181

8

8

8

98

95

95

3

3

3161

1206

1206

307

307

66

66

33

33

1268

1268

122

122

2

2

157

157

1
7153

572

572

1291

1291

17

17

137

137

610

610

52

52

1717

1717

149

149

348

348

113

113

229

229

1197

1197

24

24

14

14

1

1

585

585

96

96

920

824

450

450

55

55

319

319

96

24

24

72

72

7631

610

425

425

185

185

256

256

256

87

3

3

84

84

9

9

9

148

8

8

90

90

50

50

627

20

20

538

538

69

69

21

21

21

5873

1876

1876

2906

2906

5

5

89

89

61

61

438

438

494

494

4

4

1748

6

6

6

6

1742

1742

216

216

935

935

93

93

142

142

356

356

3534

3534

3534

308

308

448

448

14

14

320

320

549

549

646

646

42

42

1207

1207

35476
579713

1492

1492

51

51

646

646

24

24

771

771

2239

2239

584

584

396

396

163

163

1

1

481

481

504

504

28

28

68

68

4

4

10

10

189401
5838

11255
641

3

3

9409

9409

395

395

168

168

271

271

26

26

27

27

310

310

5

5

518

228

228

290

290

258
12596

83

83

27

27

614

614

123

123

40

40

594

594

203

203

106

106

3517

3517

135

135

190

190

8

8

68

68

280

280

132

132

83

83

39

39

7

7

392

392

1056

1056

4

4

28

28

3559

3559

86

86

20

20

922

922

22

22

29963
2420

772

772

4

4

41

41

1540

1540

1215

1215

413

413

55

55

2080

2080

502

502

3

3

20

20

80

80

2012

2012

134

134

20

20

5198

5198

968

968

286

286

8447

8447

210

210

23

23

164

164

992

992

2364

2364

120

36

36

56

56

28

28

56
13013

651

651

100

100

27

27

3049

3049

165

165

501

501

9

9

238

238

1913

1913

2

2

235

235

275

275

2

2

4835

4835

429

429

3

3

523

523

825

825

825

746

128

128

15

15

308

308

11

11

111

111

138

138

35

35

3490
92961

671

671

72

72

20

20

528

528

667

667

2135

2135

2

2

39

39

903

903

3624

3624

356

356

3097

3097

190

190

122

122

67

67

204

204

190

190

1051

1051

782

782

914

914

149

149

27

27

21

21

934

934

98

98

5374

5374

59

59

1171

1171

1208

1208

18

18

583

583

497

497

33

33

196

196

317

317

1385

1385

274

274

238

238

190

190

28

28

4098

4098

6164

6164

260

260

323

323

1715

1715

250

250

462

462

19

19

13

13

30

30

833

833

304

304

27212

27212

132

132

38

38

32

32

24

24

9267

9267

96

96

1794

1794

2181

2181

211

211

295

295

1672

1672

217

217

1697

1697

1698

1698

3516

2614

2614

278

278

438

438

186

186

230

230

230

3298

3060

3060

42

42

196

196

17
10476

18

18

721

721

5

5

52

52

31

31

475

475

18

18

29

29

475

475

819

819

1955

1955

1

1

555

555

18

18

110

110

85

85

312

312

1

1

180

180

84

84

51

51

442

442

95

95

865

865

563

563

288

288

17

17

326

326

1

1

3

3

569

569

287

287

121

121

52

52

430

430

215

215

189

189

1

1

4009

3631

3631

140

140

113

113

125

125

37

37

37

59446
31

20

20

20

6058

421

421

63

63

2510

2510

294

294

431

431

341

341

686

686

249

249

12

12

1007

1007

44

44

1113

449

449

664

664

929
45832

1852

1852

16758

16758

145

145

4020

4020

3

3

36

36

9181

9181

6019

6019

2054

2054

142

142

264

264

826

826

2579

2579

1024

1024

652

652

652

205

205

205

1438

119

119

1319

1319

3050

2338

2338

712

712

64

64

64

983

391

391

592

592

276

276

276

276

2
21435

355
21363

1412

1412

239

239

10

10

11

11

4751

4751

32

32

38

38

83

83

61

61

258

258

775

775

3

3

19

19

57

57

2984

2984

20

20

907

907

47

47

93

93

584

584

1760

1760

15

15

80

80

75

75

131

131

984

984

534

534

463

463

1684

1684

1674

1674

26

26

114

114

350

350

48

48

7

7

4

4

165

165

459

459

25

25

26

26

70

70

70

43304
1449

1476

1476

1476

36
27733

15755

15755

76

76

1015

1015

138

138

1997

1997

19

19

484

484

25

25

1713

1713

636

636

3586

3586

244

244

118

118

1367

1367

253

253

271

271

11822

140

140

59

59

471

471

166

166

4695

4695

666

666

350

350

37

37

3

3

564

564

1082

1082

254

254

48

48

2

2

154

154

316

316

107

107

43

43

167

167

4

4

16

16

61

61

64

64

1017

1017

480

480

159

159

55

55

642

642

824

667

667

157

157

70

70

62

62

8

8

229

229

229

229

77193
2

76726
806

301

301

70237

70237

450

450

2782

2782

2133

2133

17

17

465

460

460

5

5

347

347

347

347

554

554

38

38

270

270

246

246

146448
2406

1213
86564

6180

6180

11678

11678

1786

1786

8

8

367

367

533

533

41

41

1027

1027

91

91

273

273

461

461

9324

9324

284

284

196

196

59

59

11047

11047

41872

41872

123

123

1

1

23518
79

17

17

72

72

209

209

1290

1290

79

79

1430

1430

15

15

175

175

186

186

347

347

142

142

54

54

456

456

53

53

1545

1545

6775

6775

300

300

2

2

240

240

20

20

881

881

143

143

537

537

1095

1095

2209

2209

90

90

60

60

158

158

679

679

1186

1186

916

916

2078

2078

37

37

37

383

383

383

1293

1293

1293

189

189

189

775

93

93

17

17

89

89

564

564

12

12

19

19

19

389

389

389

73
19640

17

17

553

553

74

74

233

233

185

185

11896

11896

53

53

5

5

10

10

72

72

16

16

322

322

39

39

28

28

106

106

150

150

239

239

2447

2447

874

874

1

1

2053

2053

63

63

131

131

35

35

35

1

1

1

229
11052

3548

3548

1162

1162

1775

1775

1144

1144

1617

1617

652

652

925

925

34

4

4

30

30

113

66

66

47

47

1803

19

19

19

1784

563

563

10

10

186

186

8

8

4

4

1

1

10

10

234

234

17

17

62

62

689

689

814

92

92

92

92

237

104

104

104

108

108

108

25

22

22

3

3

485

485

253

253

75

75

157

157

20
22327

1781

1781

1216

1216

73

73

45

45

14

14

346

346

87

87

9237
6

4261

58

58

248

248

214

214

155

155

691

691

595

595

910

910

30

30

140

140

10

10

140

140

1

1

40

40

282

282

1

1

603

603

9

9

105

105

29

29

2052

61

61

725

725

393

393

171

171

79

79

135

135

488

488

2918

207

207

268

268

1733

1733

308

308

33

33

304

304

16

16

49

49

159

159

68

68

91

91

1060

1060

403

403

107

107

76

76

230

230

244

244

8420

6961

23

23

19

19

5

5

799

799

96

96

553

553

83

83

42

42

9

9

65

65

1471

1471

1

1

450

450

44

44

3

3

907

907

393

393

340

340

1

1

168

168

691

691

214

214

39

39

403

403

142

142

1459

239

239

598

598

622

622

1650

1650

1

1

1539

1539

110

110

1230

1053

1053

35

35

999

999

11

11

8

8

177

177

115

115

62

62

1685

1025

585

64

64

115

115

86

86

320

320

440

16

16

272

272

124

124

7

7

20

20

1

1

647

634

41

41

26

26

387

387

34

34

8

8

127

127

11

11

13

13

13

13

13

13

13

15

15

15

15

15

15

7692
1160741

71456
1071588

12005
16

312

312

312

1977

343

343

170

170

152

152

1258

1258

54

54

4111

311

311

223

223

4

4

1181

1181

710

710

1642

1642

40

40

5063

86

86

77

77

210

210

2471

2471

435

435

1784

1784

285

285

285

241

13

13

85

85

143

143

1891

1891

485

485

1

1

227

227

1178

1178

2031
94126

20868
20

7

7

1623

1623

1134

1134

894

894

617

617

12460

12460

3495

3495

491

491

127

127

106
31494

743

743

57

57

1565

1565

759

759

3089

3089

16

16

1734

1734

2375

2375

8

8

7

7

295

295

50

50

456

456

82

82

584

584

1744

1744

247

247

485

485

10890

10890

11

11

47

47

3

3

56

56

428

428

4372

4372

1285

1285

843

773

773

70

70

3327

31

31

241

241

1

1

59

59

11

11

223

223

1549

1549

1082

1082

130

130

759

18

18

358

358

33

33

350

350

4016
2

2331

2331

1

1

13

13

815

815

435

435

379

379

1

1

39

39

776

381

381

395

395

151

110

110

41

41

351

103

103

248

248

1018

258

258

739

739

21

21

23

23

23

1348

515

515

635

635

67

67

1

1

130

130

1012
26618

3

3

30

30

626

626

11

11

782

782

445

445

8

8

852

852

1014

1014

2

2

3

3

45

45

449

449

2263

2263

364

364

618

618

67

67

451

451

203

203

228

228

222

222

92

92

72

72

4408

4408

160

160

81

81

304

304

110

110

12

12

73

73

718

718

34

34

240

240

61

61

2924

2924

1441

1441

335

335

1113

1113

109

109

4445

4445

188

188

503

15

15

152

152

336

336

1

1

1

1

6629
385598

315

315

315

1110

1110

1110

315

315

315

91

91

91

1939

163

163

811

811

959

959

6

6

1
8377

3279

3279

18

18

198

198

179

179

2912

2912

110

110

101

101

694

694

262

262

623

623

587

587

587

7371

32

32

1086

1086

2393

2393

172

172

38

38

2425

2425

1183

1183

42

42

3543

740

740

745

745

2008

2008

4

4

46

46

3715

545

545

124

124

434

434

860

860

1752

1752

5081

14

14

1

1

231

231

140

140

236

236

4

4

2492

2492

5

5

1958

1958

4195

2774

2774

1343

1343

78

78

577

577

577

251118
13247

193

193

7975

7975

157

157

183

183

2599

2599

78

78

3829

3829

169

169

53112

53112

55

55

1043

1043

687

687

939

939

295

295

14

14

14691

14691

31

31

3477

3477

261

261

807

807

1216

1216

221

221

209

209

164

164

26

26

381

381

418

418

1222

1222

8734

8734

1257

1257

7104

7104

1289

1289

1393

1393

19

19

381

381

115

115

478

478

100

100

100

100

576

576

977

977

626

626

5

5

312

312

89

89

215

215

94

94

1157

1157

371

371

68

68

636

636

1321

1321

1330

1330

442

442

168

168

81

81

2102

2102

1489

1489

714

714

1544

1544

595

595

75

75

80

80

259

259

29

29

348

348

516

516

44

44

1480

1480

1057

1057

4

4

499

499

792

792

1554

1554

756

756

172

172

56

56

6333

6333

290

290

628

628

78

78

130

130

9235

9235

510

510

51

51

1388

1388

2921

2921

11

11

3807

3807

2083

2083

2319

2319

30

30

79

79

594

594

308

308

1823

1823

217

217

10675

10675

962

962

4360

4360

2

2

127

127

290

290

2074

2074

17

17

28

28

107

107

486

486

2149

2149

87

87

2063

2063

8254

8254

14

14

85

85

578

578

349

349

736

736

2515

2515

122

122

4232

4232

3185

3185

166

166

209

209

7

7

304

304

11

11

805

805

4987

4987

7

7

2168

2168

515

515

20

20

54

54

63

63

872

872

6

6

877

877

4584

4584

63

63

485

485

95

95

171

171

746

746

2740

2740

545

545

2

2

1087

1087

86

86

1104

1104

5

5

700

700

11020

220

220

560

560

407

407

2052

2052

139

139

319

319

3510

3510

44

44

1638

1638

1153

1153

229

229

31

31

129

129

589

589

331

331

331

437

437

437

14663
4

16

16

1549

1549

1663

1663

310

310

1996

1996

5813

5813

3312

3312

954

814

814

107

107

33

33

34

34

34

346

346

346

653

447

447

206

206

2

2

2

227

227

227

80
29652

2

2

3034

3034

9

9

13023

13023

121

121

1353

1353

8

8

408

408

3466

3466

38

38

2433

2433

31

31

3996

3996

297

297

426

426

927

927

23560
309

537

537

252

252

2

2

245

245

10396

10396

551

551

340

340

198

198

685

685

3097

3097

6

6

107

107

27

27

220

220

308

308

2749

2749

138

138

140

140

622

622

2

2

4

4

267

267

24

24

2334

2334

2801
1

3

3

320

320

1

1

218

218

2256

2256

2

2

5955

662

662

248

248

1497

1497

27

27

445

445

1207

1207

744

744

239

239

385

385

501

501

1276

573

573

573

703

703

703

335016
20391

1600

581

581

1005

1005

14

14

84

84

84

11584

200

200

429

429

16

16

515

515

77

77

3237

3237

7109

7109

1

1

4968

1700

1700

5

5

21

21

3242

3242

34

34

34

85

85

85

440

440

440

2894

2463

2463

431

431

4528

887

887

138

138

567

567

1751

1751

29

29

261

261

595

595

300

300

704

26

26

601

601

77

77

2595

2595

2595

543

543

543

240

52

52

188

188

224

151

151

73

73

12

2

2

10

10

1039

1039

1039

141

141

141

4

4

4

1281
60

158

158

170

170

893

893

224

224

224

8

8

8

4
2738

387

387

425

425

388

388

391

391

522

522

105

105

259

259

257

257

91

91

91

87668
1684

2971

2971

26

26

1110

1110

58

58

21

21

79

79

765

765

1660

1660

113

113

1119

1119

338

338

1561

1561

38

38

933

933

431

431

1351

1351

11237

11237

47484

47484

24

24

100

100

50

50

5005

5005

1

1

1252

1252

3

3

3414

3414

455

455

25

25

4232

4232

128

128

119

119

119

9
14009

1200

1200

2154

2154

1833

1833

419

419

673

673

4597

4597

807

807

275

275

132

132

1677

1677

179

179

54

54

118

118

118

662

32

32

30

30

121

121

476

476

3

3

235

235

235

448

448

448

3920

64

64

3856

3856

405

405

405

16

16

16

66

66

66

285

285

285

3717

3717

3717

5036

768

768

1541

1541

1391

1391

34

34

960

960

46

46

3

3

289

289

4

4

1747

1747

1747

33

33

33

463

418

418

45

45

12916

769

769

143

143

1

1

2028

2028

3993

3993

24

24

5359

5359

193

193

406

406

11223
12

6

6

336

336

4741

4741

1922

1922

25

25

944

944

568

568

1286

1286

1383

1383

2592

9

9

10

10

25

25

320

320

709

709

1475

1475

13

13

31

31

1896

549

549

536

536

811

811

180

180

180

2173

2173

2173

190
29335

537

537

20598

20598

1320

1320

1374

1374

333

333

324

324

217

217

2161

2161

1786

1786

495

495

3985

828

828

13

13

2826

2826

58

58

260

260

421

72

72

349

349

8332
126

5

5

2

2

383

383

78

78

1754

1754

12

12

465

465

2328

2328

1433

1433

453

453

638

638

616

616

39

39

1

1

1

852

852

852

14

14

14

25

25

25

274

274

274

324

262

262

10

10

23

23

29

29

1

1

1

13592

177

177

295

295

4037

4037

3105

3105

286

286

1720

1720

272

272

147

147

180

180

1820

1820

1553

1553

1031

634

634

64

64

333

333

267

267

267

547

547

547

1514

148

148

743

743

450

450

173

173

607

607

607

60

34

34

26

26

380

380

380

279
22769

152

152

4610

4610

6179

6179

1752

1752

641

641

1325

1325

79

79

86

86

33

33

4603

4603

50

50

502

502

165

165

597

597

38

38

106

106

1516

1516

56

56

8005

2375

2375

233

233

772

772

431

431

43

43

6

6

466

466

1272

1272

4

4

974

974

96

96

60

60

720

720

484

484

69

69

52

52

52

545

545

545

2819

2035

2035

784

784

84

2

2

65

65

17

17

727

727

727

561

557

557

4

4

351

330

330

19

19

2

2

503
11514

119

119

8666

8666

930

930

8

8

1288

1288

37
19648

143

143

582

582

62

62

27

27

224

224

63

63

26

26

9564

9564

3424

3424

82

82

65

65

40

40

660

660

1085

1085

730

730

938

938

84

84

1031

1031

211

211

570

570

332
103000

195

195

195

1423

310

310

18

18

1095

1095

280

280

280

1397

1390

1390

7

7

267

27

27

44

44

119

119

77

77

2117

184

184

1655

1655

278

278

18

18

18

50
20222

50

50

381

381

2716

2716

5105

5105

87

87

25

25

145

145

635

635

5

5

1132

1132

71

71

709

709

656

656

963

963

710

710

4

4

5

5

47

47

400

400

1307

1307

1317

1317

20

20

50

50

3

3

1850

1850

518

518

365

365

80

80

248

248

32

32

8

8

50

50

478

478

1235
76463

13

13

360

360

565

565

853

853

286

286

6363

6363

95

95

176

176

21

21

760

760

566

566

53

53

1707

1707

958

958

3635

3635

712

712

2032

2032

1154

1154

448

448

840

840

172

172

61

61

626

626

6185

6185

393

393

32

32

117

117

161

161

160

160

2

2

377

377

380

380

1179

1179

451

451

519

519

5501

5501

10086

10086

2186

2186

258

258

358

358

5

5

57

57

337

337

15169

15169

44

44

26

26

1

1

48

48

612

612

680

680

3

3

1757

1757

291

291

89

89

252

252

501

501

1081

1081

3457

3457

17

17

269

269

269

17

17

17

401

184

184

184

217

217

217

1201

233

233

233

968

826

826

142

142

3505

305

305

305

2982

301

301

2353

2353

182

182

146

146

218

65

65

3

3

136

136

14

14

39725
10

421

347

347

74

74

1136
39294

563

563

5

5

19

19

1185

1185

58

58

707

707

12267

12267

189

189

5363

5363

161

161

38

38

42

42

7

7

399

399

11

11

12281

12281

1069

1069

2

2

350

350

1157

1157

2285

2285

22387
115

12556

1382

1382

40

40

7869

7869

3265

3265

3766

3458

3458

308

308

730

130

130

197

197

2

2

401

401

1241

2

2

1096

1096

143

143

2

2

2

3969
17

917

917

3035

3035

8

8

8

4598

9

9

9

9

62

62

33

33

4

4

20

20

5

5

4527

153

153

153

506

175

175

121

121

210

210

219

158

158

61

61

1656

74

74

215

215

15

15

39

39

214

214

24

24

11

11

138

138

35

35

248

248

496

496

140

140

6

6

1

1

1005

403

403

602

602

988

383

383

168

168

404

404

5

5

28

28

35832

25921
29

5680
93

113

113

2124

2124

174

174

44

44

362

362

1599

1599

1171

1171

149

149

149

41
17868

6

6

1232

1232

9

9

97

97

174

174

71

71

9880

9880

346

346

33

33

1124

1124

178

178

984

984

1

1

2

2

2

2

8

8

2570

2570

1110

1110

1912

112

112

193

193

190

190

870

870

547

547

283

278

278

5

5

9911

781

525

525

177

177

79

79

1500

631

631

27

27

669

669

173

173

66

66

66

26

26

26

1451

617

617

153

153

29

29

348

348

300

300

2

2

2

2

2907

2385

2385

37

37

485

485

3180

895

895

758

758

4

4

17

17

139

139

995

995

4

4

16

16

41

41

311

311

84

84

84

84

84

816

816

811

430

430

379

379

2

2

5

5

5

18983

18983

186
17849

3446

3446

255

255

13962

13962

1134

201

201

154

154

3

3

776

776

270

270

270

270

270

37
11462

9830
130

15

15

15

2338
6

154

154

91

91

24

24

156

156

5

5

684

684

50

50

31

31

1

1

280

280

7

7

18

18

253

253

254

254

148

148

83

83

93

93

39
7347

8

8

14

14

13

13

516

516

237

237

344

344

587

587

1681

1681

634

634

179

179

85

85

390

390

367

367

481

481

756

756

2

2

279

279

735

735

149

149

149

149

1446
11

463
14

334

334

115

115

1
972

33

33

270

270

12

12

75

75

349

349

165

165

8

8

59

59

9416
30

2637

2637

2637

2637

16
6722

1163

11

11

851

851

169

169

98

98

2

2

11

11

16

16

5

5

6

6

6

2180

27

27

3

3

873

873

103

103

426

426

228

228

115

115

405

405

1918

15

15

1404

1404

175

175

136

136

188

188

1388

468

468

116

116

1

1

791

791

12

12

51

51

51

27

27

25

25

2

2

364
30424

908

905

33

33

33

872

872

872

3

3

3

3

29152
233

28527
581

11

11

11

319

74

74

23

23

222

222

312

116

116

34

34

162

162

31

31

31

2983
21

108

108

151

151

407

407

492

492

941

941

1

1

66

66

72

72

111

111

613

613

9
4501

14

14

88

88

1

1

4388

4388

1

1

5690

37

37

125

125

22

22

23

23

4559

4559

803

803

18

18

103

103

402

402

402

18
8398

1

1

209

209

16

16

68

68

6337

6337

501

501

289

289

19

19

940

940

865

36

36

829

829

314

17

17

124

124

8

8

165

165

27

9

9

18

18

20

2

2

18

18

12
3964

3952

3952

8

8

8

13

13

13

83

12

12

71

71

5

5

5

392

374

44

44

4

4

5

5

315

315

6

6

18

18

18

122048
3194850

1579088
138373

5

5

5

5

42

42

42

42

779

779

388

388

391

391

1436

1436

1436

1436

5823

1309

1239

1239

70

70

37

3

3

34

34

5

5

5

4472

4472

4472

5454

2960

8

8

68

68

222

222

87

87

707

707

1852

1852

16

16

2494

15

15

254

254

30

30

2098

2098

97

97

10

10

10

10

11

11

11

11

64077
23

5
5062

261

261

190

190

484

484

792

792

3

3

7

7

795

795

2240

2240

5

5

13

13

77

77

190

190

42679
559

594

594

148

148

16609

16609

70

70

1074

1074

73

73

1919

1919

1570

1570

697

697

11551

11551

93

93

1661

1661

293

293

5499

5499

269

269

4094

1287

1287

17

17

632

632

44

44

352

352

755

755

1007

1007

12219

101

101

3426

3426

661

661

792

792

434

434

107

107

68

68

14

14

400

400

4078

4078

1840

1840

291

291

1

1

6

6

46037

417
46037

555

555

652

652

70

70

129

129

27

27

605

605

371

371

3029

3029

167

167

122

122

112

112

49

49

855

855

611

611

122

122

384

384

97

97

1578

1578

517

517

656

656

40

40

6697

6697

156

156

696

696

29

29

653

653

13

13

3861

3861

55

55

1

1

6440

6440

1017

1017

223

223

167

167

7

7

88

88

111

111

467

467

222

222

98

98

850

850

1

1

30

30

1

1

518

518

2699

2699

1354

1354

516

516

406

406

34

34

362

362

215

215

750

750

195

195

1205

1205

21

21

1152

1152

2872

2872

77

77

65

65

548

548

8754

8754

283

283

2072

2072

69

69

230

230

1920

1920

547

547

570

570

778

778

719

719

1409

1409

4

4

153

153

6

6

5

5

1

1

938

534

534

534

394

165

165

2

2

227

227

4

4

4

6

6

6

892

892

892

892

456

456

456

456

3

3

3

3

3356

659

100

100

10

10

39

39

510

510

597

412

412

185

185

2100

566

566

53

53

136

136

3

3

1342

1342

180

180

180

180

17583
17

1

1

1

15

15

15

2
3626

2169

2169

165

165

727

727

54

54

6

6

7

7

145

145

259

259

44

44

48

48

1387

1387

1387

12

12

12

1710

64

64

1646

1646

92
4801

607

607

1038

1038

27

27

26

26

2979

2979

32

32

21

21

21

67

67

67

29

20

20

9

9

3

3

3

355

90

90

87

87

48

48

130

130

102

102

102

3280

49

49

8

8

1754

1754

488

488

785

785

196

196

125

15

15

62

62

48

48

712

5

5

352

352

138

138

50

50

7

7

37

37

123

123

42

42

42

119

1

1

118

118

31

26

26

5

5

839

131

131

699

699

1

1

6

6

2

2

223

178

178

45

45

64

64

64

2

2

2

149353
82

143576
6978

3352

3352

45

45

5698

5698

139

139

704

704

2779

2779

18337

18337

25

25

2833

2833

108

108

1913

1913

84

84

940

940

205

205

1134

1134

3

3

933

933

624

624

2959

2959

31897

31897

74

74

2215

2215

1788

1788

12994

12994

748

748

828

828

4677

4677

80

80

9497

9497

3514

3514

46

46

484

484

900

900

1121

1121

24

24

13

13

213

213

431

431

1085

1085

9488

9488

1381

1381

1937

1937

3

3

151

151

151

151

900

900

3188

3188

122

122

403

403

3430

3430

365

326

326

39

39

3333

1

1

3189

3189

143

143

209

208

208

1

1

1778

1718

1718

60

60

10

10

10

1363

685

35

35

113

113

537

537

678

59

59

13

13

36

36

9

9

513

513

48

48

3072

3072

28

28

1894

1894

1

1

929

929

68

68

4

4

148

148

3

3

3

3

799

799

190

190

420

420

4

4

176

176

9

9

892

892

563

563

329

329

415

415

28

28

387

387

202

202

202

202

1422

1422

792

792

627

627

3

3

13382

11

11

11

1380

15

15

396

396

763

763

206

206

2162

1245

1245

690

690

227

227

25

25

25

12
9657

1213

1213

116

116

329

329

476

476

9

9

491

491

299

299

21

21

72

72

553

553

1299

1299

1985

1985

258

258

1

1

138

138

1250

1250

169

169

63

63

896

896

7

7

147

2

2

145

145

267

267

91

91

4

4

36

36

2

2

45

45

8

8

68

68

13

13

125

125

125

125

19

19

19

19

2918

2918

686

686

2232

2232

265040
7330

1872

10

10

912

912

139

139

811

811

212

212

212

5

5

5

6249

3603

3603

164

164

1406

1406

1076

1076

40742
112

2620

2620

1255

1255

50

50

1672

1672

952

952

159

159

57

57

430

430

107

107

535

535

1163

1163

174

174

1866

1866

13

13

1980

1980

744

744

538

538

3

3

608

608

2514

2514

98

98

17

17

221

221

25

25

15

15

15941

15941

37

37

109

109

2

2

217

217

5614

5614

148

148

141

141

605

605

1241

1241

1241

385

385

385

511

511

511

640

95

95

296

296

118

118

131

131

834

559

559

10

10

265

265

115

115

115

318

318

318

2411

24

24

2387

2387

863

3

3

749

749

111

111

10268

328

328

6

6

3793

3793

643

643

113

113

2295

2295

3090

3090

9801

3530

3530

1853

1853

4405

4405

13

13

1927

520

520

1197

1197

59

59

7

7

1

1

143

143

270

270

270

126

126

126

3

3

3

111780
5499

276

276

181

181

92

92

1056

1056

418

418

60

60

14

14

29

29

65

65

2783

2783

33

33

1971

1971

1927

1927

2

2

23

23

66

66

3930

3930

152

152

333

333

38

38

227

227

7

7

20

20

74

74

4908

4908

672

672

13855

13855

4250

4250

11

11

6631

6631

875

875

1143

1143

91

91

2665

2665

3821

3821

357

357

244

244

1

1

317

317

1093

1093

632

632

138

138

1041

1041

23295

23295

63

63

6261

6261

3

3

845

845

54

54

778

778

313

313

9455

9455

4

4

807

807

62

62

49

49

2746

2746

1006

1006

1362

1362

311

311

433

433

44

44

1028

1028

14

14

265

265

561

561

599

599

599

6776

3920

3920

583

583

932

932

305

305

2

2

387

387

191

191

3

3

453

453

7

7

7

1782

800

800

90

90

505

505

293

293

94

94

2154

109

109

43

43

2002

2002

37153
585

4

4

5134

5134

431

431

281

281

7628

7628

62

62

4700

4700

242

242

2444

2444

739

739

6

6

859

859

508

508

137

137

32

32

475

475

361

361

245

245

1119

1119

29

29

383

383

8403

8403

224

224

1808

1808

46

46

261

261

7

7

229

229

229

1444

1444

1444

4
16516

125

125

90

90

252

252

1159

1159

140

140

13410

13410

1336

1336

477

477

477

14

14

14

14

5876

64

64

64

1373

1373

1373

325

200

200

115

115

10

10

4114

86

86

1

1

441

441

3586

3586

39593
33

6422

1

1

3766

3766

479

479

87

87

2089

2089

1422

198

198

305

305

919

919

5598

715

715

271

271

1426

1426

1301

1301

1175

1175

10

10

700

700

7920

1564

1564

712

712

289

289

702

702

2009

2009

408

408

2167

2167

69

69

49

3

3

44

44

2

2

16

16

16

1

1

1

116

114

114

2

2

3682

1523

1523

19

19

1794

1794

311

311

20

20

15

15

3477

3477

3477

9846

355

355

370

370

1376

1376

3412

3412

25

25

270

270

250

250

53

53

38

38

173

173

14

14

857

857

1493

1493

21

21

1139

1139

84

84

84

487

487

487

440

81

81

359

359

2786

2786

2413

2413

373

373

1694

1694

1694

1694

27

27

27

27

29

29

29

29

3315

88

88

88

521

18

18

503

503

5

5

5

2701

143

143

2531

2531

2

2

16

16

9

9

5265

338

338

338

2355

67

67

29

29

1080

1080

70

70

21

21

975

975

113

113

11

11

11

92

92

92

2469

598

598

22

22

1812

1812

37

37

847
466494

447862
24189

1776

1776

80

80

12474

12474

773

773

3474

3474

4779

4779

280

280

881

881

2409

2409

372598

372598

323

323

14526

14526

400

400

50

50

24

24

1060

1060

53

53

1950

1950

731

731

265

265

759

759

4008

4008

4
17785

1761

1761

51

51

1055

1055

27

27

14887

14887

2161

2161

1789

1789

372

372

1854

1854

1854

1854

3

3

3

3

332

332

332

332

1815

1815

1

1

1032

1032

120

120

270

270

9

9

259

259

51

51

73

73

2037

351

183

183

168

168

1686

415

415

1183

1183

61

61

27

27

193102

193102
7699

1554

1554

1362

1362

78

78

2139

2139

1158

1158

209

209

1450

1450

1657

1657

45

45

22

22

898

898

11

11

356

356

178

178

262

262

8

8

56

56

2097

2097

359

359

49

49

99

99

230

230

36

36

1167

1167

123

123

868

868

25

25

8049

8049

967

967

533

533

64

64

1931

1931

170

170

343

343

33

33

1543

1543

392

392

1156

1156

825

825

63

63

1097

1097

93

93

1760

1760

488

488

317

317

29

29

1321

1321

173

173

10

10

6276

6276

720

720

42

42

106

106

424

424

714

714

41

41

85

85

320

320

314

314

1128

1128

405

405

306

306

187

187

59

59

15

15

50

50

54

54

563

563

320

320

510

510

11113

11113

405

405

285

285

503

503

20

20

423

423

1334

1334

5

5

309

309

269

269

1934

1934

149

149

273

273

8

8

141

141

198

198

5

5

839

839

94

94

27

27

698

698

187

187

949

949

1219

1219

2552

2552

1684

1684

3183

3183

800

800

660

660

38

38

1133

1133

1487

1487

10

10

173

173

48

48

1638

1638

607

607

7160

7160

142

142

322

322

30

30

13

13

390

390

1017

1017

1481

1481

176

176

1116

1116

84

84

278

278

438

438

1172

1172

286

286

35

35

139

139

1888

1888

10434

10434

3234

3234

3

3

4015

4015

20

20

31

31

430

430

790

790

30

30

2256

2256

714

714

48

48

186

186

1101

1101

5

5

471

471

519

519

1037

1037

2

2

646

646

11625

11625

46

46

993

993

339

339

5

5

1048

1048

2692

2692

212

212

2

2

129

129

6808

6808

3

3

2241

2241

879

879

55

55

51

51

79

79

1565

1565

32

32

225

225

1478

1478

2

2

703

703

35

35

565

565

57

57

75

75

815

815

253

253

784

784

320

320

700

700

106

106

268

268

5616

5616

123

123

6

6

699

699

1998

1998

375

375

80

80

3864

3864

9

9

416

416

791

791

10

10

141

141

10

10

1588

1588

315

315

512

512

2679

2679

186

186

16

16

44

44

98

98

4

4

92

92

61

61

33

33

62

62

1

1

37

37

1996

1996

320

320

1036
42784

29827

476

476

112

112

1575

1575

690

690

576

576

43

43

451

451

25904

25904

11921

11921

11921

1383

1383

19

19

15

15

34

34

902

902

6

6

407

407

382

382

382

382

7366

7366

3353

3353

20

20

187

187

2413

2413

1393

1393

262

262

182

182

80

80

553

553

553

553

247

247

61

61

147

147

39

39

1075

1075

438

438

637

637

240

181

181

181

59

5

5

54

54

28780

1354

1354

1354

11808

1410

1410

1579

1579

56

56

2076

2076

727

727

496

496

4063

4063

168

168

60

60

1173

1173

595

260

260

335

335

9
15023

3666

3666

1318

1318

10030

10030

9038

286

98

98

188

188

5905

270

270

3298

3298

2337

2337

2847

719

719

880

880

1248

1248

52

52

23

23

29

29

41

41

41

41

96

96

96

96

6830

2

2

2

95

95

95

175

175

175

4133

235

235

739

739

319

319

3

3

1608

1608

1229

1229

1165

7

7

243

243

915

915

839

364

364

469

469

6

6

180

180

180

241

241

241

33

33

33

33

278

6

6

6

272

272

272

242

242

242

242

1034

974

974

974

60

60

60

330

330

330

330

480

480

480

480

1662

1662

860

860

802

802

69

69

69

69

110

110

110

110

143

143

143

143

638

638

633

633

5

5

70

70

70

70

19

19

19

19

697

673

673

673

24

24

24

350

350

350

350

660

660

660

660

10002

40

40

40

1
9962

5773

5773

2051

2051

3

3

413

413

61

61

94

94

538

538

82

82

885

885

61

61

908

908

555

555

16

16

337

337

328

328

328

328

67

67

67

67

1955

913

381

381

532

532

1042

1042

1042

3295

3295

2

2

2

26

25

25

1

1

2251

376

376

14

14

697

697

1164

1164

1016

959

959

57

57

1486427
131547

90

90

90

90

9

9

9

9

5051

5051

14

14

967

967

9

9

79

79

1661

1661

7

7

133

133

15

15

77

77

53

53

1

1

1896

1896

20

20

39

39

65

65

6

6

9

9

715

715

705

705

10

10

565

565

540

540

25

25

1576

1576

893

893

683

683

748

748

536

536

212

212

2163

2163

2163

2163

1969

1969

1969

1969

205

205

198

198

7

7

791

487

487

487

304

139

139

8

8

157

157

59

59

5

5

54

54

1641

193
1641

76

76

14

14

270

270

883

883

30

30

14

14

87

87

16

16

58

58

4322

874

867

867

7

7

25

25

25

2351

2351

2351

1072

266

266

806

806

18221

6144
1

293

293

93

93

14

14

73

73

9

9

13

13

346

346

115

115

264

264

2844

2844

67

67

58

58

78

78

100

100

470

470

455

455

360

360

308

308

90

90

93

93

12077

67

67

2391

2391

9

9

14

14

13

13

560

560

226

226

188

188

2

2

167

167

6337

6337

1361

1361

33

33

517

517

186

186

6

6

306630
10883

8288

57

57

1968

1968

695

695

1648

1648

2215

2215

1085

1085

192

192

307

307

121

121

15461

2990

2990

258

258

4

4

1467

1467

2252

2252

5953

5953

313

313

2224

2224

24671
504

3

3

1321

1321

392

392

1249

1249

5

5

97

97

657

657

42

42

289

289

76

76

4491

4491

15541

15541

4

4

3912

863

863

89

89

742

742

2142

2142

8

8

9

9

59

59

4

4

4

16

16

16

1185

1185

1185

663

54

54

609

609

24552

1171

1171

159

159

860

860

27

27

19

19

216

216

18086

18086

1449

1449

63

63

65

65

2312

2312

125

125

3964
6

11

11

3947

3947

14

14

14

62

62

62

17379

457

457

310

310

8

8

520

520

47

47

13785

13785

935

935

419

419

898

898

1
8796

7

7

15

15

9

9

123

123

4

4

360

360

381

381

593

593

1506

1506

131

131

88

88

15

15

78

78

2

2

2275

2275

285

285

351

351

81

81

2491

2491

4823
105169

1

1

98574

98574

115

115

212

212

5

5

16

16

1291

1291

132

132

30075

8

8

1116

1116

2412

2412

17

17

1067

1067

720

720

683

683

989

989

1073

1073

427

427

1148

1148

1378

1378

71

71

1758

1758

78

78

197

197

36

36

888

888

13542

13542

644

644

1015

1015

178

178

321

321

309

309

313

313

313

2119

700

700

48

48

1034

1034

337

337

626

626

626

246

109

109

137

137

4053
6

617

617

7

7

103

103

41

41

91

91

82

82

48

48

509

509

244

244

1014

1014

30

30

111

111

1150

1150

83

83

83

1575

1575

1575

67

25

25

42

42

16

16

16

5587

5587

5587

139

139

139

4366

3765

3765

601

601

146
19061

24

24

831

831

8902

8902

14

14

588

588

119

119

295

295

237

237

6

6

7523

7523

376

376

95

95

95

13190
187

11165

11165

318

318

17

17

148

148

59

59

4

4

114

114

18

18

1

1

1

1

1158

1158

346

346

346

346

8

8

8

8

14

14

14

14

13455
465990

2283

1994

1994

168

168

5

5

46

46

70

70

942

942

942

82

82

82

365

365

365

443

194

194

249

249

52430
841

1888

1888

122

122

1038

1038

5200

5200

2396

2396

1322

1322

28

28

820

820

38

38

4847

4847

28

28

73

73

2544

2544

3804

3804

997

997

2083

2083

6164

6164

360

360

1662

1662

1

1

1405

1405

3810

3810

201

201

1042

1042

1252

1252

659

659

134

134

81

81

145

145

584

584

12

12

1685

1685

27

27

124

124

2856

2856

12

12

186

186

11

11

331

331

15

15

1602

1602

156

156

156

20

20

20

264

264

264

15594
93

84

84

291

291

13

13

1136

1136

8

8

9

9

647

647

1291

1291

1

1

179

179

7

7

3523

3523

3956

3956

94

94

3984

3984

57

57

206

206

7

7

8

8

6897

3414

3414

207

207

82

82

86

86

31

31

432

432

2550

2550

95

95

493

493

493

14182
1

1089

1089

5596

5596

2838

2838

4482

4482

176

176

5041

524

524

1881

1881

41

41

755

755

582

582

1258

1258

140

140

140

432

108

108

264

264

60

60

13706

880

880

1

1

40

40

27

27

11308

11308

1046

1046

108

108

103

103

193

193

740

740

740

134

22

22

31

31

12

12

69

69

7601

3025

3025

1650

1650

28

28

611

611

1193

1193

177

177

917

917

324

324

324

1018

968

968

50

50

166
20912

633

633

247

247

64

64

13840

13840

1696

1696

73

73

3659

3659

534

534

2099

420

420

534

534

31

31

495

495

82

82

9

9

1

1

41

41

43

43

239

239

204

204

28

28

28

30

30

30

850

164

164

686

686

929

923

923

6

6

18974
283768

21

21

1555

1555

866

866

1224

1224

269

269

145

145

66

66

2192

2192

725

725

2734

2734

200

200

193

193

47

47

8

8

262

262

359

359

8

8

54

54

155

155

6

6

13

13

329

329

295

295

117

117

88

88

47

47

606

606

108

108

556

556

500

500

720

720

622

622

377

377

514

514

52

52

658

658

1663

1663

186

186

554

554

1855

1855

5013

5013

5068

5068

275

275

16

16

2699

2699

17742

17742

68

68

13

13

202

202

403

403

334

334

42

42

124

124

139

139

171

171

4554

4554

51

51

232

232

18

18

283

283

305

305

5695

5695

301

301

9

9

45

45

33

33

13

13

1464

1464

175

175

803

803

5933

5933

68

68

7961

7961

3316

3316

155

155

41

41

95

95

1410

1410

104

104

614

614

3533

3533

9678

9678

231

231

5

5

536

536

5303

5303

277

277

1119

1119

2

2

1

1

108

108

224

224

1034

1034

2298

2298

27

27

1398

1398

1002

1002

1148

1148

15

15

6262

6262

1226

1226

481

481

6972

6972

38

38

1153

1153

22679

22679

227

227

2931

2931

82

82

2173

2173

538

538

1297

1297

891

891

616

616

7006

7006

188

188

17

17

1600

1600

2278

2278

24

24

136

136

108

108

507

507

14933

14933

73

73

473

473

168

168

60

60

1251

1251

158

158

485

485

108

108

56

56

152

152

66

66

612

612

3

3

2911

2911

94

94

66

66

5874

5874

38

38

7544

7544

9706

9706

4

4

2754

2754

988

988

1052

1052

2211

2211

534

534

516

516

263

263

63

63

435

435

157

157

5541

5541

1175

1175

342

342

222

222

4092

4092

579

579

2277

2277

538

538

1155

1155

974

974

33

33

4769

4769

347

347

128

128

653

653

1441

1441

23

23

44

44

5390

5390

315

315

79

79

207

207

430

430

62

62

888

888

908

908

1823

1823

29

29

134

134

2083

2083

10973
3

150

150

1328

1328

1412

1412

1

1

470

470

12

12

1332

1332

105

105

2207

2207

109

109

628

628

3

3

228

228

1

1

1014

1014

27

27

957

957

388

388

459

459

139

139

9050

3824

3824

456

456

114

114

880

880

105

105

2487

2487

617

617

480

480

87

87

12

12

12

597

29

29

353

353

96

96

119

119

2373

41

41

41

713

713

713

1026

1026

1026

1

1

1

592

457

457

135

135

98

98

98

98

3487

3487

3487

3487

4786

1714

1339

1339

78

78

39

39

243

243

15

15

1740

41

41

265

265

86

86

1161

1161

91

91

96

96

236

230

230

3

3

3

3

1096

140

140

31

31

925

925

1010

1010

1010

1010

644

644

279

279

365

365

600

600

377

377

21

21

73

73

122

122

7

7

445

445

216

216

77

77

136

136

16

16

6141

6141

10

10

520

520

8

8

4172

4172

618

618

47

47

536

536

230

230

334

76

23

23

53

53

258

254

254

4

4

1751

1751

342

342

22

22

663

663

177

177

547

547

12708
197134

5806

50

50

5756

5756

336
2

45

45

80

80

209

209

1348

1348

1348

523

99

99

424

424

1181

1181

1181

47693
4114

252

252

2

2

72

72

3

3

773

773

41

41

129

129

25

25

243

243

100

100

121

121

189

189

581

581

1013

1013

1

1

79

79

2

2

1167

1167

272

272

5

5

566

566

334

334

19

19

2217

2217

595

595

7

7

651

651

862

862

1367

1367

439

439

459

459

329

329

34

34

45

45

2106

2106

171

171

132

132

27

27

5101

5101

123

123

466

466

239

239

1472

1472

318

318

32

32

215

215

110

110

26

26

57

57

1

1

95

95

13

13

118

118

615

615

34

34

961

961

22

22

154

154

1363

1363

77

77

11

11

147

147

1581

1581

259

259

41

41

6

6

90

90

44

44

92

92

213

213

211

211

4

4

205

205

625

625

2296

2296

20

20

509

509

87

87

170

170

58

58

1

1

56

56

1147

1147

1934

1934

22

22

106

106

50

50

73

73

1336

1336

996

996

6

6

1318

1318

625

625

37

37

78

78

644

644

1010

1010

407

407

15

15

7

7

80

80

80

584

584

584

6222

3444

3444

31

31

571

571

983

983

1141

1141

52

52

30661
44

8

8

2800

2800

2818

2818

281

281

2493

2493

64

64

138

138

22015

22015

15

15

15

12833
24

777

777

104

104

89

89

117

117

11707

11707

15

15

48889
294

948

948

25

25

1345

1345

47

47

1625

1625

416

416

3812

3812

238

238

629

629

3736

3736

58

58

1452

1452

286

286

162

162

476

476

1307

1307

6277

6277

225

225

1417

1417

23

23

211

211

154

154

143

143

221

221

1907

1907

40

40

3

3

722

722

38

38

10739

10739

727

727

33

33

5

5

6

6

220

220

8642

8642

280

280

4919

3182

3182

525

525

146

146

55

55

29

29

197

197

38

38

2

2

745

745

20165
897

89

89

7

7

8

8

1747

1747

254

254

388

388

487

487

145

145

102

102

432

432

907

907

2

2

2

2

4567

4567

1386

1386

3474

3474

801

801

781

781

4

4

27

27

17

17

1738

1738

718

718

101

101

159

159

925

925

576

576

576

1671

5

5

115

115

1343

1343

208

208

924

80

80

840

840

4

4

1089

1089

1089

1089

485

485

485

485

2174

1391

152

152

265

265

64

64

710

710

174

174

20

20

6

6

783

104

104

679

679

4032

4032

1469

1469

10

10

297

297

1470

1470

304

304

11

11

471

471

10510

10510
1

67

67

693

693

933

933

745

745

5

5

455

455

33

33

5

5

9

9

23

23

712

712

258

258

862

862

835

835

248

248

2186

2186

142

142

441

441

996

996

208

208

166

166

15

15

415

415

57

57

237

237

237

237

2166

2166

2166

2166

7790
2

4638

4638

4638

2851

74

74

8

8

2485

2485

284

284

24
299

275

275

2304

2304

2164

2164

125

125

15

15

2560

2560

755

755

6

6

739

739

195

195

285

285

580

580

3181

3181
1

1178

1178

948

948

64

64

211

211

571

571

208

208

7

7

7

7

1

1

1

1

2562

2562

45

45

1851

1851

666

666

1399

1399

1259

1259

140

140

5639

5639
206

1601

1601

23

23

2578

2578

1089

1089

142

142

1783

1783

386

386

19

19

75

75

1303

1303

45

45

45

45

12086

12086
16

1854

1854

7581

7581

835

835

132

132

17

17

195

195

197

197

45

45

20

20

672

672

213

213

134

134

154

154

21

21

10

10

10

10

20

20

20

20

49769
367

7948

7948

7948

11470

3418

3418

6766

6766

1286

1286

6485

452

452

32

32

2828

2828

3173

3173

23499
324

891

891

6854

6854

6758

6758

301

301

164

164

514

514

1337

1337

5488

5488

851

851

17

17

1415

1415

1413

1413

2

2

750

750

505

505

77

77

168

168

248

248
46

77

77

125

125

9

9

9

9

7374

896
7374

425

425

57

57

33

33

6

6

11

11

542

542

2

2

297

297

761

761

4344

4344

242

242

242

242

5534

634

517

517

105

105

12

12

209

209

209

2000

878

878

649

649

9

9

392

392

59

59

13

13

623

461

461

106

106

24

24

32

32

1626

745

745

20

20

181

181

131

131

429

429

120

120

442

442

442

7

7

7

7

3329

3329

1690

1690

308

308

653

653

585

585

93

93

1620

1374

1374

1374

246

246

246

242

242

242

242

5031

5031

313

313

232

232

285

285

78

78

643

643

171

171

833

833

482

482

823

823

433

433

559

559

179

179

2578

2578

123

123

18

18

258

258

26

26

298

298

1004

1004

7

7

807

807

37

37

31

31

31

31

7

7

3

3

4

4

8839

99

13

13

86

86

2750

2006

2006

9

9

33

33

14

14

688

688

306

286

286

20

20

5188

2676

2676

470

470

634

634

426

426

759

759

11

11

212

212

154

154

154

87

87

87

131

131

131

124

124

124

665

665

12

12

653

653

2116

2116

2116

2116

7

7

7

7

750

14

14

14

736

736

736

333

333

313

313

20

20

8

8

8

8

6958

391

4

4

11

11

376

376

6567

716

716

7

7

2373

2373

3471

3471

372

372

372

372

238

238

238

238

269

269

269

269

860

860

860

860

1223

1223

87

87

1062

1062

74

74

608

608

373

373

70

70

6

6

82

82

77

77

3

3

3

3

21

21

21

21

442

442

442

442

841

841

841

841

25

25

25

25

1136

1136

153

153

527

527

281

281

175

175

1465

1465

1

1

1464

1464

478

478

478

478

1

1

1

1

3712

53

53

53

127

127

127

2357

1021

1021

169

169

1020

1020

7

7

19

19

113

113

8

8

1175

1074

1074

101

101

68

68

2

2

66

66

1938

1843

966

966

27

27

310

310

540

540

95

95

95

177

177

10

10

167

167

83342
663

6360

155

155

4494

4494

22

22

1689

1689

1394
45101

4649

4649

589

589

5362

5362

4

4

2399

2399

5778

5778

4687

4687

310

310

14331

14331

814

814

1294

1294

24

24

1356

1356

13

13

5

5

1105

1105

13

13

47

47

565

565

362

362

19886
225

168

168

806

806

723

723

241

241

4448

4448

1712

1712

8824

8824

138

138

2333

2333

48

48

162

162

58

58

15

15

15

842

842

842

1953

1953

1953

897

897

897

7326

33

33

811

811

90

90

145

145

7

7

1325

1325

1586

1586

3329

3329

299

14

14

26

26

243

243

16

16

247

114

114

114

133

133

133

907

907

907

907

2175

2175

2175

2175

11427

7602

12

12

3

3

38

38

2461

2461

1367

1367

66

66

134

134

967

967

489

489

665

665

1400

1400

3705

302

302

3403

3403

13

13

13

107

58

58

49

49

2061

555

6

6

27

27

14

14

3

3

505

505

1506

488

488

114

114

104

104

800

800

527

527

527

527

758

758

421

421

254

254

51

51

32

32

4369

4369

960

960

905

905

2504

2504

35161

2325

52

52

80

80

2

2

62

62

11

11

1375

1375

35

35

8

8

14

14

95

95

210

210

158

158

223

223

38

38

38

29799
47

311

311

603

603

1161

1161

4

4

632

632

1304

1304

1075

1075

146

146

5936

5936

1303

1303

1016

1016

17

17

75

75

1635

1635

1367

1367

3849

3849

906

906

280

280

4495

4495

69

69

59

59

153

153

3356

3356

898

118

118

780

780

1413

1413

1413

37

37

37

651

651

651

6239

5097

12

12

387

387

3318

3318

274

274

19

19

167

167

670

670

143

143

107

107

1142

1142

1142

986

986

176

176

405

405

405

405

16

16

16

16

4800

1050

5

5

1045

1045

3750

291

291

531

531

1055

1055

98

98

1707

1707

68

68

130

130

130

130

3992

251

251

251

251

3741

3429

45

45

155

155

378

378

382

382

14

14

72

72

426

426

196

196

420

420

1341

1341

312

312

312

3766

3766

185

185

117

117

68

68

1622

1622

1622

1622

111

111

21

21

2

2

55

55

33

33

1297

1297

1297

1297

551

551

551

551

2894

1328

1328

1079

826

826

253

253

249

1

1

161

161

87

87

1566

1566

1560

59

59

1

1

2

2

430

430

1068

1068

6

6

6

1435

1435

539

495

269

269

210

210

16

16

44

27

27

17

17

896

592

6

6

505

505

78

78

3

3

304

4

4

300

300

4229

4229

679

679

674

674

5

5

3550

3550

3529

3529

21

21

6840

186

186

126

117

117

9

9

60

60

60

298

298

298

282

282

16

16

65

65

65

65

65

4164

4164

4164

150

150

4

4

478

478

437

437

524

524

1725

1725

25

25

821

821

2127

2127

12

12

12

2115

134

134

1753

1753

228

228

778

778

778

778

587

587

191

191

35126846

3547460
352310

39

39
8

2

1

3

1

1

23

1

11

7

1

3

122
2476

25

25

229
1201

207

57

560

128

6

89

3

174

160

148

829
1128

11

1

3

4

17

7

11

7

172

1

33

16

84

3

4

1

30

39

3

19

5

34446

514

20

92

2

15

220

11

1

1

1077

5

3798

115

53

63

251

73

49

85

1956

519

13

56

8

54

5

47

11

175

265

50

16

302

26

6

28

269

5

10

8

17

29

186

442

1

649

1

13

2

1

36

4

1

11

10

6

26

4

1

1

3

10654

2

8

10

33

373

1

9

11568

8

3

2

1

1

2

1

178

8

58

193

40

9

1

1575

2

11

7

10

3

4

268

82

49

79

18

183

3

2

4

154

17

2

16

2

1

124

15

4

7

27

275

2

8

13

1

5

5

5

3

1

2

6

268

8

92

60

60

5

18

37

24

13

526674

333

333

333

333

333
8

26

26

34

11

23

142
26

35

43

38

123

97

26

526341
494

17070

6409

156

156
2

119

11

3

13

49

8

35

1

1

32
34

2

10

10

10

9

1

70

70

42

42

9
28

19

19

6173

423
6173

1281
425

65
95

30

55

104

104

44

44

63
71

8

58

58

2

97

97

37

37

12
19

7

90

90

91

7

21

1

2

18

42

74

19

19

187

3

39

13

2

30

1

13

1

3

65

1

1

3

1

1

7

1

2

20

20

3376
1151

51
58

2

2

2

1

272

1

2

3

1

48

54

37

1

3

48

12

27

35

60
68

2

6

37
51

8

3

3

86

86

85
114

3

6

12

2

6

47
36

1

8

2

6

6

21

21

27

27

79
92

5

7

1

1
10

3

6

41

41

21

21

44

44

45

45

22
35

13

13
43

30

20

20

29

12

6

3

2

1

3
2

1

2

55
64

9

34

34

30

30

25
11

14

68

68

8

8

17

37

162
139

9

1

1

2

2

6

1

1

61
24

37

6

44

36
30

6

45

45

35

35

4
156

152

128
53

23

49

3

3

5

5

32

32

30

30

22

18

4

50
23

5

22

54
179

49
39

10

7

7

13

13

22

22

10

10

24

3

1

17

3

89
707

230

103

94

1

31

31

1

1

68

68

11
7

4

68

68

1

74
73

1

32

31

1

59
58

1

3
75

71

1

10661

10661

250
10661

10248

51

545

1036

1849

1316

6

1149

220

83

642

745

818

1

1026

397

364

163

163

508777
2156

2
20882

17623

593
17623

266

13

80

165

2

5

1

14001
1010

1576
300

117

1

116

93

2

917

147

173
414

241

241

30
164

1

133

214

214

7

2

5

3

2

97

97

116
133

15

2

748
33

240

240

475

240

240

178

368
130

5

233

1706
814

74

221

11

203

104

1

98

1

112

79

11

8

102

7

63

101

101

125

125

246

246

358
612

32

32

222

3512
6062

736

1062

36

458

95

163

105

58

93
2763

921
469

13

386

46

7

18

1

1

16

444

444

1287

1287

3257

3257
208

2584

2584

465

465

36268
485739

44414
639

2520
26178

1114
3309

74

2121

529

625

108

128

69

25

36

69

532

354

19995

3802

2685

6537

3944

3027

3169
16268

5016
192

1132
1844

540

195

27

2

316

171

1

492
1700

33

6

27

496

679

521
1280

32

11

50

136

107

2

13

14

494

35

1

5513

65

437

30

1

4734

246

2570
194

285
325

12

22

12

10

6

1176
2051

9

527

41

408

74

4

339

28
1329

538
152

386

386

763
427

216

55

10

31

14

65

215178

23860
215178

148447

12386

7

3378

21387

1155

3181

37913
1471

6600

6600

4549

4549

6288

6288

19005
105

8914

9986

4700
9423

2172

2551

12862

7422

5403

3761

3440

13837

6605

4429

1858

2

2

15106
4141

1500

9464

3981

5

231

36

91

136

800

776

4

3391

13

1

27763

27763

189879

189879
29249

76565

42307

4584

2

1678

34

6

5882

1672

11432

4519

105

4344

2352
536

22

915

879

17680

4203

23

6488

6946

20

1570

1570

11223
17668

368

3

20

4645

563

19

72

4

74

59

94

398

45

8

158

15

10

7

9

54

5

71

26

10

17

3

74

76

4

13

25

669

29

89

132

1075

83

443

33

179

769

413

225

2

1097
2

1095

1

6

63

1008

17

14471
41738

1051

25884

1

650

1062

286

2122

1181

1164

355

142

497

2058

5185

53

2908

3961

1862

33

293

163

1908

332

1960
1324

636

110

236

34

1

22

22

14

151

34

8

3

1

57

57
20

37

30

7

572
11

25
412

367

61

306

4

9

7

23

9

14

126
2

15

109

860

118

95

478

33

12

29

13

82

2
142

8

8

132

2154
159940

1

1

1

92067
313

116

23
43

10

10

10

73

18

20

7

8

17

3

15
68

53

6

4

4

6

17

9

7

14543
67

2
7965

1828

1828

111
1827

315

3

5

9

2

6

6

3

10

13

13

15

1

4

1

6

11

1

5

6

20

8

10

1

33

1

10

8

20

1

10

6

17

4

7

2

5

7

11

1

5

8

22

22

29
124

22

32

41

6

18

10

2

5

56

56

122
8

101

5

4

3

7

17

9

5

3

5

13

14

16

7

6

1007

882
1007

4

9

1

62

16

3

2

3

2

8

6

2

2

3

2

8

8

62

1

1

61

1

1

6135

423
6135

132
923

58
34

24

4

11

9

102

1

39

8

2

2

2

1

1

20

8

18

3

3

1

2

15

15

4

11

43
83

40

2

22

2

9

5

84
548

17

8

9

1

13

2

4

4

11

6

3

8

11

12

20

22

9

6

7

2

16

49

8

17

16

103

8

6

13

6

14

5

9

32

9

1

1

1

363

9

3

37

5

4

6

11

3

6

25

11

2

15

9

2

9

7

3

3

7

3

5

21

5

4

2

16

6

5

5

4

1

3

1

8

7

13

8

2

4

1

7

6

2

8

6

3

2

9

2

3

2

12

8
232

19
224

107

11

10

6

2

8

3

6

10

13

2

15

5

16

12

3

22

14

14

7

1

25

166
7

139

1

14

2

8

12

11

1

5

6

2

3

6

7

8

2

5

8

16

7

8

7

20
4

5

11

175
1221

221

10

5

11

11

13

5

3

3

10

4

5

8

3

12

2

12

3

5

17

20

18

5

13

2

12

1

8

9
81

11

39

6

11

9

13

9

6

6

1

14

14

14

2

348
621

5

9

235

6

6

1

7

1

2

5

5

2

11

6

2

3

3

8

21

7

22

13

2

8

14

4

16

5

1

35

19

19

9

3

7

5

43
4

1

17

6

11

21

2

62

62

1

22

4

19

2

14

34
119

41
68

9

3

1

1

1

7

5

17

12

3

1

1

8
595

114
554

428
241

1

2

23

8

6

5

1

5

2

14

8

111

5

88

3

2

3

2

24

1

2

7

3

4

1

1

7

9

2

4

13

1

4

3

10

1

7
1

3

2

1

3

1
5

4

15

15

15

15

18

15

3

1470
200

495
784

73

10

5

4

2

1

2

6

5

12

12

14

8

3

3

9

9

1

40

11

5

1

2

7

10

8

5

5

11

20

9

3

7

5

16

4

12
13

1

1

4
6

2

476

1

24

13

7

6

8

13

7

5

1

10

7

11

3

8

26

7

19

6

12

7

2

6

6

9

5

8

5

33

23

6

8

18

3

23

9

5

9

16

3

18

18

3

15

2

6

16

4

622
13

14

1

1

12

595
104

13

1

90

30

52

37

15

39

36

11

52

145

11

5

9

18

14

16

16

15

9

1

4

17

2

8

22

25

5

7

13

6486

6409
24

6232
38

1
9

7

4

3

1

3281
304

14
244

9

96

4

19

5

16

15

25

12

25

15

21

11

10

26

14

3

1

1

1

2
21

14

5

5

47
68

8

13

96
28

68

49

2

17

152
9

7

14

6

21

21

37

1

2

3

6

15

10

20

17

40
13

1

14

12

47
59

7

7

5

81
213

8

110

1

7

33

11

10

30

13

1

1

3

14

4

8
9

1

81
49

6

20

4

2

9
50

17

24

46
32

7

7

11

68
258

2

14

6

13

5

1

30

30

85

1

1

1

6

4

8

12

1

126
58

13

6

6

12

15

2

10

4

2

2

9
2

7

1

15

1452

14

10

11

1

6

4

17

30

3

1

16

5

2

19

5

2

15

14

2

15

11

12

38

6

4

16

12

13

2

18

22

11

22

12

17

8

11

23

25

32

20

3

12

16

11

5

8

37

17

8

9

21

36

1

19

9

1

19

1

3

12

2

14

7

11

17

25

22

16

24

28

9

16

10

12

5

14

35

12

20

8

12

46

4

7

2

9

18

13

15

19

10

10

26

8

16

8

24

25

20

3

6

14

8

11

8

14

14

8
20

8

4

350
1537

198

21

82

14

3

2

32

1

6

7

4

1

1

1

2

1

1

2

5

3

5

1

3

68
440

8

3

5

174
6

4

117

19

19

9

20

20

14
170

16

7

7

4

6

27

8

14

14

60

134
4

19

111
33

3

14

22

4

14

7

14

4

9
411

1

267
38

13

40

81

17

33

4

4

37

4

1
5

3

3

1

2
10

8

38
11

13

14

81
23

18

9

9

5

2

4

8

21

83
27

9
34

4

1

7

13

4

18

18

48
272

12

12

7

7

93

27

53

13

17
112

13

7

19

15

10

31

62

62

62

1

14

1

16

6

13

11

80
402

26
231

110

80

15

15

4

4

1
87

15

64

7

10
252

15

15

20

20

100
2

98

15

70

13

107

10

30

45

22

27
1

2

2

2

24

4

17

3

72

72

72

7

15

7

11

12

20

197

15

4

16

10

6

16

10

12

61

6

11

6

2

1

7

8

6

153

153
1

19
26

7

7

126

9

1

5

9

1

2

14

3

4

4

10

22

14

16

11

1

64

64

64

64

15

6

7

10

8

3

15

13

13

13

4

3

1

9
3

1

1

1

3

15
218

6

3

3

106
16

62

24

24

4

49

49

3

3

1

2

24

8

9

4

1

2

15

15

8
7407

718

718

28
718

68

4

8

16

1

2

7

4

2

10

1

13

622

606

6

1

5

1

2

4

2

4

10

14

2

6

1

2

4

3

4

3

2

36

5

5

2

4

2

7

1

25

7

15

2

8

2

16

3

5

3

9

1

6

13

1

1

5

2

1

6

14

10

1

8

2

2

5

5

11

7

7

4

3

6

4

4

16

3

5

1

1

9

1

5

3

1

2

1

7

4

4

4

13

1

7

1

10

1

4

2

4

13

4

4

17

7

5

2

9

3

8

1

3

6

17

6

1

2

11

8

7

7

9

1877

1877

1877

1877
9

1862

1

1

2

13

3

1

2

3

3

4

3

4

2

2

11

3

1

1

4

2

1

2

4

2

1

3

2

7

8

2

5

3

8

14

1

1

6

3

1

4

2

2

2

1238

10

1

10

1

4

1

2

8

2

13

3

5

2

4

2

1

2

1

3

2

9

3

1

3

2

2

2

1

9

1

18

6

4

4

9

2

1

6

1

4

8

47

3

6

2

2

4

18

2

8

11

2

1

3

2

1

1

11

3

1

4

4

2

2

4

1

2

1

2

1

1

1

4

2

5

6

1

1

6

4

4

16

2

5

12

2

1

4

7

3

1

1

2

15

2

7

3

10

2

3

7

3

1

4

1

1

1

2

3

4030

4030

10
4030

41
2

24

15

6
88

2

40

11

2

3

11

13

40

3891

3854

5

6

13

13

774

774

774
27

3

3

400

1

5

5

16

2

3

2

23

1

2

2

5

1

2

3

5

2

10

3

3

3

8

16

3

1

6

6

6

5

5

7

10

3

18

12

6

4

3

2

13

15

6

20

1

13

14

5

1

14

17

1

6

2

12

3

1

6

3

12

15

1
344

328

3

4

12

1

3

1

22

2

5

10

8

4

12

2

9

6

13

6

3

3

3

15

1

2

1

3

5

4

10

2

5

1

4

11

2

1

3

5

9

5

12

2

8

9

2

2

13

3

4

2

8

8

4

30

11

4

35
34748

6227

2773

2773
621

1349

9

8

17

2

2

1

1

17

2

6

5

1

1

1

258

19

1

6

18

9

25

13

10

3

12

8

1

4

21

9

15

10

2

9

3

10

12

7

1

20

1

17

16

9

11

10

15

36

5

11

7

5

9

1

26

2

6

15

14

11

15

5

33

4

5

16

9

1

9

1

6

3

2

2

13

4

13

26

1

1

2

2

20

17

26

17

7

14

4

9

15

2

13

29

3

4

2

26

8

2

2

17

42

6

1

6

9

12

4

20

2

31

253
124

15

112

3

22

1

5

1

32

47

1

2

212
550

2

6

5

2

3

7

45

1

249

12

2

1

40

18

3

2

6

6

5

1

5

7
20

1

2

6

1

1

2

26

57

6

20

8

3

8

15

3

3454

3454
193

16

16

2
20

9

9

2

98
48

10

1

5

5

5

1

1

1

8

8

6

14

14

58

8

10

40

4

4

11
73

9

6

17

5

4

21

1
123

10

8

41

44

5

5

9

9

12

45
2

5

32

6

520
243

12

12

16

17

86

27

10

6

103

42

19

23

2167
723

8

3

1

3

7

5

9

7

4

7

3

1

1

2

13

8

6

7

2

12

4

2

2

6

60

6

1

3

3

2

2

2

21

3

18

4

4

2

5

13

8

4

8

9

7

2

42

11

27

1

3

8

34

9

4

2

2

18

12

1

11

3

2

1

2

7

7

4

31

2

9

3

8

2

30

62

4

9

5

31

4

13

46

4

6

6

11

3

8

4

4

5

3

3

138

1

1

2

10

3

3

5

2

1

1

7

5

12

4

1

2

2

3

24

10

26

1

1

10

1

97

9

4

1

1

2

3

7

5

1

2

5

7

16

19

7

13

39

3

9

20

9

4

7

2

1

14

39

1

3

13

6

4

1

2

4

31

33

2

7

5

3

8

67

2

6

3

1

9

2

1

4

1

9

24

5

3855

3855
1

1091
107

32
839

1

3

44

759

57

3

3

30

1

12

108

63

7

8

3

5

2

4

1

17

1

1

16

2

3

9

2

1

1

1

4

1

1

2

4

4

377

1

1

7

145

1

1

5

3

1

1

1

2

4

6

5

1

5

3

1

1

1

1

1

2

2

1

9

3

1

29

3

1

1

4

2

23

3

2

2

1

3

1

2

2

1

2

1

147
14

98

6

2

1

2

2

8

3

4

10

4

1

14

1

1

14

1

2

3

7

6

6

5

5

30

4

6

6

3

8

3

103
925

137

5

30

16

4

9

17

2

20

19

11

4

685
18

18

574

1

1

31

19

19

22

22

20

21

12

29

27

1

16

55

30

18

10

17

2

7

29

17

25

13

28

16

21

29

2

14

41

34

1691
126

61

8

7

2

1

1

4

17

2

3

5

1

5

1

1

5

4

11

16

1

5

2

1

4

3

11

11

22

1

7

7

7

3

2

2

1392

1

2

7

4

2

5

2

8

6

1

1

3

3

2

1

2

2

7

1

4

1

1

24

4

1

8

5

4

3

7

6

5

7

3

3

3

3

2

4

9

3

5

2

15

8

2

2

2

1

2

2

14

2

1

4

7

3

3

4

2

11

2

1

1

12

8

2

2

7

16

7

3

5

4

3

3

472

7

5

15

2

11

16

2

1

3

1

3

3

3

5

2

1

1

1

6

3

1

2

1

13

26

4

3

3

1

2

5

7

21

3

7

3

2

3

2

5

4

1

5

1

10

3

2

1

1

4

4

6

4

22

3

1

8

1

2

3

1

22

2

10

4

4

3

1

2

1

3

1

1

1

4

14

3

8

1

1

4

5

4

7

1

2

1

9

1

3

2

5

2

6

6

1

2

2

7

2

1

3

5

3

3

10

1

8

5

6

3

2

5

2

4

10

3

1

3

13

9

6

1

4

9

9

2

1

8
63

1

1

11

2

1

4

3

4

11

2

8

7

1

2

4

24631
7

358
9279

177

1

6

3

76

4

87

4255

4255
2090

217

217

132

2

2

26

1

1

52

1

41

36

5

43
1907

31
415

313

313

14

14

57
54

3

198
32

42

40

2

16
94

9

45

11

13

5

12

2

2

1

23

15

15

15

15

31
414

127
16

25

32

20

12

22

32

20
1

19

19

25
104

2

11

11

11

54

1

42

1

2

5

1

3

5

24

1

47
15

19

13

9

4

2
43

5

5

25

11

45

45

118
792

29
54

25

111
48

57

2

4

4

13

13

13

52
6

46

10

36

40
1

6

2

31

11

20

17
8

9

75

62

13

7

6

177

64

1

7

1

1

1

2

2

12

31

9

4

8

9

1

1

7

9

3

1

1

2

3

3

23

1

22

20

2

40

3

37

25

7

5

69
6

63

2
3066

3064
931

2

2012

30

41

3

38

44

13

1779

64

53
119

2

52

52

12

877

877
26

27
35

1
8

7

7

490

35

35

100
455

189

43

28

61

57

157
28

123

6

9

26
326

259

259

41

36

36

5

5

341
22

24
86

62

62

62

62

23
233

162

37

67

5

53

48

48
19

29

29

199

8

8

8

191

191

191

191
84

45

62

6

14749
494

41

6

14

3

18

545
4531

11

11

30
38

2

2

6

24
11

13

14
122

12

31

34

31

4

6

3

7

1

6

4

1
27

26

9

9

3

5

33
3

30

5

25

47

36

4

7

5

8

3

9

11

21

21

18

3

7

7

7

9

9

143
5

11

16

111

6

6

19

16
19

3

11

11

96
44

27

17

8

1

8

8

11

6

5

113
26

17

17

2

6

9

53

61

38

23

3

3

1

2

180
123

43

13

16

1

13

14

108
55

28

23

5

15

1

4

4

1

5

5

1

1

1

1

2

2

2

77
59

1

1

17

4

4

30
6

24

24

922
1387

8

16
57

6

4

1

4

5

21

167
72

3

1

1

3

5

2

4

1

18

1

5

2

3

2

1

3

2

1

6

8

4

2

3

1

2

6

1

1

2

2

26

4

11

1

5

4

1

15

16
44

1

3

4

1

15

1

3

26

21
14

1

2

3

1

20

15

15

28
29

1

18
7

7

4

19
15

1

1

2

19

19

916

5

2

20

8

4

3

22

9

3

6

19

7

11

4

6

6

2

5

1

18

4

5

3

9

2

7

6

2

8

5

2

23

13

10

8

2

4

6

2

2

22

16

565

10

7

6

6

119
33

24

23

23

39

49
95

4

3

1

42

21
2

19

196
99

66

1

5

13

12

8

2

4

1

15

4

1

4

14

2

5

6

2

2

21

667
59

4
56

5

2

17

6

11

10

10

3

5

443

2

1

3

8

4

4

9

3

13

27

128

11

4

6

5

18

16

10

1

12

14

18

22

11

5

8

4

11

3

5

9

4

3

6

1

34

46

23

15

8

6

10

5

2

3
63

1

53

9

6

1

4

2

9

22

4

2

3478

13

37

4

1

2

9

8

9

6

14

2

14

18

2

66

11

18

4

6

2

5

3

14

4

7

8

4

5

14

1

6

1

4

11

11

3

17

2

1

21

11

13
135

18

7

4

6

10

9

54

1

7

5

1

10

4

10

8

55

1

7

25

5

10

5

2

22

8

4

6

5

7

7

6

6

17

17

16

8

8

8

10

17

4

7

16

13

11

33

13

2

9

6

3

22

7

9

19

15

10

3

15

9

26

10

5

15

10

3

2

14

8

16

13

4

4

5

3

3

3

12

5

2

18

6

1

7

1

2

2

9

5

22

7

28
53

25

25

18

11

5

29

2

5

22

1

25

4

17

6

8

4

17

12

9

26

1

9

15

15

2

6

1

1

38

47

21

4

20

13

9

9

14

9

3

24

15

4

1

3

9

30

23

3

2

14

8

17

78

36

35

5

2

1

17

6

1

10

5

15

5

1

10

10

17

29

13

10

10

16

17

7

8

2

10

4

1

11

9

1

5

1

1

7

7

7

7

17

4

1084

1

11

14

7

7

981
39

355
31

180

2

10

18

3

11

1

2

17

10

21

2

12

3

11

2

1

4

15

5

9

3

11

7

7

27

5

15

12

10

7

12

12

22

6

9

587

2

14

2

16

9

18

12

8

6

10

19

7

6

8

8

16

22

8

13

1

17

9

6

6

8

6

21

7

4

13

208

9

7

8

19

3

12

14

5

8
165

1
74

8

3

5

18

10

9

20

5

15

83

7

33

14

5

24

4
262

93

93

36

22

22

14

2

2

2

1

1

126

126

19

60

22

25

1912
31

48

11

15

16

5

1

33

15
33

6

12

2
1233

153
899

7

140

1

7

1

14

17

15

15

10

24

2

1

7

26

53

35

10

8

1

60

42

17

12

18

25

9

47

27

14

9

17

22

14

4

19

23

14

39

20

25

15

220
39

5

2

10

64

4

5

15

6

16

18

9

18

11

10

7

19

26

112
19

16

10

6

8

11

9

6

27

12

6

9

42
194

22

71

49

7

4

11

15

17

5

20

2

84

35
2

16

17

18

18

24

24

7

7

98
6

14

11

21

16

11

19

19

1
158

14

20

77

46

4

6

13

23

273
2197

36
194

79

68

11

1

1

9

40

6

11

29

29

10

19

88
354

191

75

11

5

1

1

1

8

11

1

9

27

45
11

34

18

9

7

715

17

9

28

11

19

10

3

11

1

10

7

6

1

18

1

14

2

1

14

18

11

8

1

2

174

10

1

4

16

1

14

40

2

6

8

11

13

18

9

5

31

24

3

12

4

2

1

4

56

3

20

92
55

32

25

1

6

5

495
179

145
284

4

2

9
11

2

35

7
16

1

8

61
71

6

2

1

1

32

13

18

1

21

10

10

11

11

596
1

486
1

19

19

95

1

28

9

5

3

2

2

5

5

14

8

6

4

3

371
12

22

10

5

7

20

13

5

11

12

13

8

26

17

9

10

134

23

11

1

1

5

8

1

8

5

2

14

4

9

36

6

8

18

11

37

37

37

72

72

14

58

14

8

2

3

18

13

3
27263

285

285

18
230

13
2

4

1

1

2

1

6

179

23

4

13

1

7

4

3

11

2

1

8

9

9

9

6

1

1

1

4

5

9

6

2

7

12

7

12

1

2

20
1

7

5

2

9

1

1

1

55

55
24

4

27

2

19

3

1

2

12783

12783

12783
636

773

23

2

5

10

1

31

26

19

2

33

34

5

42

16

11

8

5

8

6

26

8

58

4

6

20

16

38

29

9

1

17

25

20

14

3

29

2

17

13

8

53

1

12

23

3

14

10

7

1631
627

7

26

13

10

9

8

14

10

2

7

9

26

7

25

12

14

10

9

2

25

54

10

6

3

26

5

3

54

8

356

21

2

25

12

11

24

11

4

14

12

28

4

4

21

9

18

1

7

14

11

11

15

17

5

3

8

19

25

30

23

59

2

5

14

72

1

28

747
427

8

99

24

151

25

2

76

6

25

12

5

20

18

511
1020

165

60

119

60

11

18

4

26

1

88

76

7976
361

57

37

11

6667
6649

5

1

7

3

2

3

801

15

58

46

35

28

17

4

29

45

28

17

21

47

21

40

11

6

7

18

163

22

16

52

23

32

39

12106
62

1828

1332
128

292

12

20

29

2

8

8

5

11

2

44

6

12

17

6

9

62

6

1

18

7

7

912
77

691

13

2

11

57

14

60

19

19

10

4

5

139

101
8

24

12

3

17

37

12

17

8

38

6

19

13

338

8
338

242

57

117

68

88

206
4836

86
1011

7

451
44

28

20

19

21

8

19

9

24

31

18

12

10

102

14

7

12

24

9

5

11

1

19

15

19

1

21

18

12

97

78

24

15

25

14

19

126

1

5

18

19

2

5

12

21

21

11

11

244
40

12

7

18

15

60

9

2

11

16

14

8

19

13

1

32

27

2464
101

38
1303

171
840

6

32

4

3

1

6

4

8

16

13

1

2

11

21

188

38

10

5

14

2

2

10

4

11

5

11

3

4

10

27

1

6

16

2

2

5

18

1

18

8

9

7

9

18

11

4

7

40

15

13

6

7

29

15

13

22

5

7

20

11

14

3

14

9

28

28

4

283
49

52

11

9

13

53

14

43

14

15

2

3

3

6

34

5

108
31

23

21

10

7

4

3

16

2

231

1

8

1

9

14

3

8

9

22

18

10

61

20

14

2

4

27

829
13

1
236

6

3

2

1

119

23

84

240
20

10

5

2

9

9

4

7

28

25

5

6

7

8

68

12

6

3

35

3

5

4

3

24

8

8

2

2

1
8

1

6

7
25

8

8

4

4

2

7
40

18

12

8

4

3

29
169

7

2

43

27

34

24

3

7

6

14

7

6
33

17

10

3

2

1

4

6
55

25

6

6

12

6

816
14

471
58

9

16

8

8

10

17

38

19

5

17

12

8

7

30

6

3

3

10

28

24

10

15

9

2

2

11

2

3

6

7

4

1

20

29

5

2

13

5

2

1

1

12

2

7

7

41

41

74
219

9

1

6

7

9

33

5

8

14

5

1

6

7

4

17

5

15

26

31

1

7

8

7

2

1

5

33
13

1

9

8

1

10

87

66

15

4

11

8

3

3

3

2

17

21

21

13

13

13

13

239

6

3

13

12

10

2

12

3

13

39

4

3

3

19

8

8

1

1

5

16

45

11

2

10
5380

619
3

12

12

58
283

15

28

1

41

1

4

1

3

4

4

1

2

1

3

4

10

3

6

6

4

3

4

4

23

10

11

15

6

7

24

5

9

3

2

2

21
55

10

4

20

14

6

62
3

7

12

4

19

2

15

7
202

13

126

56

692

295
692

35

13

8

28

3

8

8

6

11

3

17

5

6

1

136

1

6

7

4

18

6

7

6

16

4

9

3

13

8

11

10

7

2

6

6

13

10

12

4

43

13

1670
39

24

45
283

43

10

32

13

9

22

2

16

18

13

11

27

12

3

1

11

4

1

17

66
480

3

24

8

11

29

37

16

17

10

103

5

20

21

54

2

1

11

132

13

10

445
47

37

7

10

15

35

8

30

37

88

61

8

2

5

16

2

8

7

13

12

58

35

59

4
166

70

14

14

6

3

36

12

6

15

19

5

3

11

110

42

42
14

4

24

16

1

7

28
780

244

6

3

9

3

27

11

1

3

5

8

12

1

6

5

18

3

2

9

1

3

1

6

8

15

5

20

8

3

9

4

19

10

5
65

33

5

4

4

18

9

4

5

32
51

7

12

25
12

2

2

5

4

23
83

11

11

7

10

10

8

14

44
2

10

26

6

63
231

3

10

3

4

11

6

3

1

7

5

63

1

17

2

1

2

9

2

3

4

10

9

3

4

8

10

5

6

2

2

2

2

11

69
1456

397
24

7

42

17

6

31

24

19

19

37

39

25

38

3

3

9

9

30

19

15

898

45

15

2

1

2

26

10

1

41

31

3

24

2

11

3

70

22

7

14

24

8

9

20

33

59

16

3

40

24

17

36

4

24

44

26

17

3

10

24

43

12

15

37

1

19

92

28

4

23

19

18

111
1

43

22

21

67
16

10

10

15

26

2086

2
2086

58
884

172

2

1

7

9

1

6

3

1

1

6

7

2

15

3

11

2

3

2

6

3

7

1

4

9

3

5

14

3

1

2

12

7

11

2

24

10
24

3

11

11

172

11
172

19

67

4

8

17

4

1

5

5

9

4

10

22

7

7

7

14

5

13

9
458

59
25

5

16

1

12

25
4

11

2

1

4

4

2

1

50
10

12

1

1

3

14

9

6

3

48

6

6

6

30

3
113

1

2

1

3

1

2

11

88

4

2

27

1

1

9

39

3

2

1

65

4

26

26

9

2

2

13

13

1
23

9

2

11

21
8

3

4

4

2

4

10

1

1

1

1

7
18

9

1

1

85
1167

119

4

1

5

2

19

4

1

5

3

2

13

1

3

14

7

12

9

3

4

7

9
185

18

22

13

89

22

31

12

24

34

8

2

109
597

5

42

18

9

1

7

241

2

9

15

1

3

12

11

2

1

4

12

5

3

10

1

40

7

13

17

25

16

9

23

29

1

9

23

3

13

1

23

5

2

16

1

15

12

2

10

171
15

8

25

4

14

20

47

5

1

9

1

2

6

3

7

13

33

33

33

7391

4508

4508

128
4508

634

4

5

3

5

9

5

24

10

14

9

48

27

7

71

1

4

6

25

14

76

2

11

4

12

5

27

2

5

4

2

11

2

3

18

17

27

26

16

2

2

44

7

7

11

2461

1021
272

44

141

303

57

4

92

31

13

54

7

19

2

11

2

1

12

10

10

10

1258
138

19

45

33

218

7

15

237

8

13

1

5

3

10

27

23

2

10

27

14

10

1

1

1

16

4

1

4

1

1

12

18

1

11

8

4

10

7

36

28

121

3

12

54

148

56

13

18

19

21

9
69

16

10

17

11

6

17

103
3

29

22

16

5

11

33

1285
3

16
3

6

7

7

31

31

80

17

63

26
119

35

24

7

4

17

1

18

4

4

14

57
35

8

14

14

24
34

10

208
32

17

3

5

151

86

7

3

7

27

1

3

17

230
28

5

186

38

3

40

21

8

36

40

8

3

123
507

28

177

6

84

3

11

2

2

45

3

18

42

29

18

208

208

208

186

186

16
22

2

4

4

2675

8
2675

26

26

19

7

7

2158
132

209
7

2

3

11

175

2

4

2

1

2

9

6

2

2

4

16

5

14

3

2

11

4

1

2

26

2

3

2

8

21

2

10

2

3

1

1

2

8

3

16

7

7

9

2
85

10

28

29

16

29
261

3

7

4

19

7

136

1

6

7

5

7

5

2

8

6

4

9

1

11

1

13

5

8

8

10

19

15

6

8

1

7

4

14

1

1430

12

29

12

7

4

2

9

7

22

9

8

13

13

5

12

2

7

10

2

4

2

24

9

5

2

6

1

3

2

5

20

3

1

6

2

5

2

6

27

12

2

22

1

15

18

8

10

3

14

3

12

14

15

50

12

13

4

17

25

4

19

4

4

7

2

40

7

5

9

14

8

20

7

7

6

4

3

1

1

281

1

14

4

6

7

35

8

20

6

23

5

3

1

19

1

4

16

3

2

14

33

7

8

3

1

4

3

9

2

2

2

14

5

4

1

16

2

28

2

2

6

8

18

4

11

9
25

2

3

2

1

11

87

87
1

86

3

14

7

14

2

2

24

20

49
396

108

8

15

53

11

21

101

99

1

6

14

36

1

6

3

21

8

2

1

2

19
129

16

16

3

17

13

4

27

19

12

9

9

32

32
1

7

4

3

5

10

9

17381

6

7

12

5

7

5

5

2

13

9

2

22

10

9

4

32

1

20

7

3

2

2

5

2

1

1

3

2

12

3

6

6

1

9

5

1

20

7

24

35

3

1

7

12

4

2

9

5

35

6

10

16

28

11

5

3

2

10

4

3

1

7

1

3

1

4

7

13

10

20

7

11

1

1

7

4

11

8

7

6

2

4

23

1

11

6

3

6

6

27

10

3

5

15

3

8

3

21

7

2

1

4

6

10

18

1

2

7

5

18

11

15

8

3

4

2

15

1

11654

6

15

5

2

15

8

10

1

18

4

3

31

2

7

2

1

1

22

16

6

2

10

10

9

2

13

3

9

23

1

31

6

9

12

1

3

2

8

6

12

3

31

2

6

9

8

8

13

14

3

6

10

12

15

23

7

2

15

17

2

6

9

4

15

8

3

38

8

1

22

3

10

5

9

3

7

3

12

4

16

10

12

11

1

3

1

1

11

1

7

10

4

2

4

20

2

5

1

8

4

2

20

1

5

4

4

17

1

43

2

9

4

1

13

4

4

5

5

14

2

13

4

13

1

6

7

12

14

8

4

5

2

10

17

8

2

8

11

18

7

1

13

7

8

4

6

6

6

21

4

19

1

11

3

2

8

22

3

1

13

10

6

13

6

6

5

10

4

8

3

6

11

1

7

6

10

7

1

4

8

4

3

7

1

5

1

1

1

5

7

14

6

5

11

16

27

5

13

4

12

13

2

10

10

2

8

12

14

5

6

9

8

24

5

4

5

2

4

13

10

6

18

9

3

3

15

7

7

3

13

2

10

4

2

4

17

31

7

5

10

13

4

1

7

13

14

5

7

1

9

20

22

16

5

3

15

13

32

3

1

4

8

9

41

7

7

1

4

5

4

5

2

12

20

13

8

12

18

10

7

6

25

5

5

8

7

5

32

8

9

3

16

11

13

8

11

10

6

9

3

10

3

13

2

1

5

3

9

3

7

12

10

4

21

2

3

6

8

3

21

13

40

2

2

1

9

10

8

11

7

16

5

11

7

18

9

3

6

3

1

2

7

12

6

51

6

3

11

3

11

18

1

4

7

11

1

4

6

8

17

7

6

2

9

9

36

8

13

6

27

8

4

2

2

20

1

4

10

29

2

6

2

4

5

21

25

5

17

2

13

9

12

20

2

23

2

4

31

28

5

5

2

10

5

7

15

4

3

18

19

2

2

11

4

2

4

7

7

3

7

23

2

32

11

20

4

4

9

14

4

12

13

20

1

2

4

11

10

4

22

5

19

5

8

1

7

19

5

2

4

4

25

10

5

19

3

6

4

4

6

11

16

5

5

7

31

1

18

5

11

9

6

9

4

15

15

22

9

1

38

3

14

9

3

7

5

8

6

8

5

14

6

3

2

12

10

3

35

11

6

8

16

14

42

8

5

10

1

10

2

7

6

4

9

23

16

1

5

20

30

2

10

2

4

31

2

5

3

25

1

45

3

17

9

4

22

9

9

4

21

3

26

1

4

6

12

11

10

7

20

11

17

15

8

3

10

11

14

20

5

11

6

16

11

10

6

1

9

6

11

6

6

4

7

1

6

7

4

2

4

6

4

12

6

3

16

8

4

1

3

18

32

10

2

6

4

8

14

1

1

3

2

2

17

17

4

3

9

10

6

16

1

3

7

2

6

10

6

20

2

3

12

11

7

1

18

17

4

4

10

3

7

9

9

3

9

5

7

2

2

6

31

2

2

5

9

2

21

9

4

19

12

1

3

10

5

1

1

9

11

19

23

36

17

11

2

9

6

3

2

15

7

15

37

2

3

10

15

9

1

3

14

9

28

7

14

11

2

5

5

5

8

9

2

24

5

19

17

4

16

5

7

4

22

8

20

26

6

2

5

25

15

6

4

1

8

22

1

24

5

9

2

15

53

8

23

5

42

9

1

6

5

7

3

5

22

13

6

33

36

6

17

7

1

12

14

28

4

17

19

1

4

10

4

21

9

5

14

24

11

3

8

6

6

17

8

3

6

4

7

32

3

1

4

6

8

8

4

3

9

1

6

14

27

4

1

2

4

5

9

3

7

26

11

4

4

3

12

5

4

26

14

13

5

12

6

4

1

8

5

7

5

1

22

10

9

1

10

6

4

27

11

1

11

16

3

6

6

3

13

1

15

7

21

1

7

25

16

8

38

6

5

1

31

3

1

2

6

3

9

22

3

15

18

4

10

5

7

5

14

16

4

4

16

7

21

1

10

12

9

8

2

25

11

8

1

8

8

3

14

13

2

2

2

8

22

6

9

4

13

21

10

19

3

9

2

4

8

33

4

1

6

3

5

1

5

14

5

13

32

5

4

11

5

7

3

2

15

4

9

15

4

3

7

7

9

25

13

7

3

7

4

5

6

2

1

10

14

6

5

8

2

10

10

21

7

8

3

3

2

9

6

31

4

8

2

16

17

10

3

20

12

6

8

37

17

9

8

6

4

3

13

9

8

13

14

5

9

7

14

4

5

7

14

2

11

5

1

2

8

6

9

4

4

1

7

1

20

12

2

18

6

1

14

10

10

5

12

38

16

5

12

2

5

12

8

5

8

9

43

7

8

7

8

1

13

3

14

8

1

20

8

17

6

1

4

18

4

3

8

16

15

49

3

10

3

12

14

2

13

8

6

2

7

10

23

18

10

2

8

20

2

22

28

4

13

19

7

2

6

3

7

1

2

6

22

13

2

9

40

13

6

4

4

2

3

10

3

2

7

5

5

4

1

6

36

3

19

16

9

3

5

9

1

26

9

23

6

20

9

3

9

12

19

4

5

11

17

36

20

22

8

9

7

17

3

2

8

15

7

4

11

13

6

7

4

14

7

19

20

5

3

2

3

5

7

1

16

20

4

18

5

8

5

9

15

7

3

18

4

20

18

8

8

4

11

5

4

11

8

11

10

11

5

21

1

5

4

6

10

7

7

9

3

1

9

2

4

3

5

8

16

17

11

20

2

13

9

2

10

2

4

15

9

6

2

6

5

8

9

10

1

11

6

10

27

15

16

7

23

2

27

10

7

8

4

1

8

3

9

3

32

11

14

10

4

3

3

8

20

9

6

21

7

2

8

4

2

1

5

10

11

2

13

20

4

12

16

13

13

1

1

4

1

9

5

2

2

2

6

12

3

2

1

9

16

2

2

4

19

19

9

2

1

3

17

1

10

4

22

4

1

68

2

2

19

4

2

6

1188

9

9

4

5

13

83

1

1

2

2

5

4

24

5

4

7

9

4

5

17

6

2

6

1

1

1

10

9

16

4

2

14

3

2

2

3

2

5

6

1

5

1

7

3

1

5

10

15

52

1

1

12

3

4

2

9

4

17

1

8

27

5

7

8

11

8

4

12

6

12

4

10

4

1

2

6

13

4

4

2

15

4

2

1

2

12

2

3

1

6

15

5

15

12

12

1

7

1

1

14

2

1

6

8

3

2

59

3

4

4

14

3

7

7

23

14

2

6

12

15

5

61

2181

12
326

2

24

33

16

8

6

10

17

10

5

12

13

9

2

11

2

3

4

15

11

11

25

6

6

9

12

13

9

10

12

4

22

1055
10

8

15

12

3

26

3

3

10

10

11

1

32

4

24

48

1

7

20

42

4

83

5

17

1

3

26

112
6

1

28

9

1

8

5

4

1

6

16

11

5

4

9

6

4

4

12

29

2

21

23

7

7

18

55

15

9

1

5

12

17

13

55

30

6

12

2

1

10

11

35

10

40

35

5

1

27

10

11

12

3

5

80

6

14

4

2

1

3

20

3

22

274

10

30

4

16

25

3

1

1

2

6

28

5

8

2

3

5

13

4

4

1

3

2

7

14

2

6

7

3

23

2

3

24

3

1

3

5

3

4

3

10

64

4

7

50

1

5

7

3

16

26

43

51

6

84

19

13

5

47

47666

47666

47666
9

3186

3186
274

31

12

12

7

19

19

8

8

57
19

2

2

12

1

23

2797
184

2524

3

24

26

5

3

18

15

4

17

968
44471

1331
336

788
196

365

227

227

207

60

147

37630
1081

1067
35295

8010
27944

57

616

75

5

37

33

6

66

550

264

15934

48

1903

324

61

61

30

31

30

30

30

2405
569

63

24

13

4

4

4

17

64

146

2

3

14

26

101

17

1

8

24

24

1

23

1455

153
440

125

93

86

7

30

8

31

182
93

32

18

39

92
255

8

6

31

108

32

2

20

2

2

3

5

40

2

10

141
2845

117

2400

96

95

1

63

3

25

157

157

785
348

77
24

7

32

1

13

26

26

200
24

7

13

13

156

38
34

4

16

16

80

80

469

1

3

1

6

6

29

14

1

120

10

10

17

37
6

3

27

1

23

1

1

59

17
50

30

3

21

31

19

18

1

15

1

4

980

980

980

2956
451

30

30

60
13

8

3

12

13

11

3

8

21
5

7

4

5

5

29

29

62

9

53

1882
685

2

1

4

17

15

8

2

11

7

52

4

13

4

1

1

40

21

6

14

15

26

7

61

3

9

28

28

1

8

3

17

10

5

4

13

21

14

18

648

15

2

2

9

13

59

10

4

1

13

7

2

53

274

12

1

4

2

6

35

8

11

16

34

22

10

8

9

6

6

10

6

1

8

4

23

23

55

55

38

38

65
305

2

15

15

7

6

36

93

10

14

4

65

15

14

3

18

10

6

606

593

12

1

635

45

45

590

10

3

4

20

12

53

3

1

15

4

24

7

4

3

2

32

2

19

4

1

10

2

2

1

10

8

6

1

12

32

12

3

5

13

7

2

20

10

1

7

5

1

11

3

21

1

11

9

3

17

13

2

2

6

1

8

26

1

6

2

17

8

15

3

7

4

3
4

1

14

14

14

1912036

1912036
187

1878022

1878022

182489
1878022

3204
619

224
707

120
166

46

18

14

4

1

9

198

119

65

54

167
154

13

13

122

144
285

141

1

1

7

108

13

1

10

357

1

152

105

99

89

317
835

283
185

1

20

77

13

5

2

6

8

8

11

1

10

3

1

1

2

3

3

100
235

1

1

133

3

2

2

2

7

1

26

10

3

23

1

3

1

49

23

1794

376

593

250

44

2

529

1090978

74

403

1079385

9

72

23

131

18

417

260

183

4

1

840

270

304

236

9

1149

38

42

104

100

21

152

256

438

232

1231

123

282

3

1301

101

221

311

447

1232

480

75

12506
38181

77

25

31
71

40

6

25

2

7

52

1676
304

56
74

18

1

12

5

140
84

56

8

1

8

1

1

7

4

2

2

16

6

17

131
169

38

5

17

12

4

66
329

14

249

239

2

4

4

12
11

1

1

94
101

7

4

3

20

23

47
54

7

2

5

77

178
145

2

31

8

2

3

1

1

15

1

39
42

3

3

22
44

22

22

32

23
15

8

8

37

13

1

16

6

1

32

108
151

43

33

10

99

563
2803

2240

57

355

301

180

134

1032

181

35
54

19

9

10

361

361

660

660

257

257

156
43

4

109
104

5

1

2

2

27

315

315

640

8

600

28

4

18
154

93

43

7

3

2

28

3

14237

5

3

61

2891

19

95

7

115

2309

49

237

31

35

99

178

24

930

1068

2130

2

209

671

69

187

687

154

30

120

224

98

6

39

116

1092

74

38

135

23
401

320
218

102

4

7

6

5

3

15

2

13

10

30

7

58
33

25

3

22

36
269

77
127

50

50

40

42
66

24

7

17

49
1706

1657

1372

285

84

90
431

74

74

51

42

174
124

50

27

23

87
323

57
56

1

1

32

5

27

25
18

4

3

52

39
24

1

8

6

2

4

31

67
41

26

6

1

19

74
267

22

16

30

30

51

22

40
52

12

24
223

51

51

148
64

84

5

12

2

7

12

25

9

10

2

6
57

51

51

83260
280100

718
720

2

2

345
131

214

214

49
251

202

66

15

3

1

35

82

47
124

77

58

19

633
620

13

13

4

4

2

2

29
2

27

27

2308

202
241

1

38

10

1

10

3

2

3

8

1

167

2184
389

238

641
514

127

58

69

648

30

132

118

6

19

12

3

276

11

34

7

140
268

128

1

3

1

1

9

17

9

2

3

6

12

1

63

68

818
577

241

240

1

1
426

425

425

54
189

135

15

64

39

15

2

16867
7272

9595

5314

4281

112799

1

6

1043

274

5

1

200

1

60

29

32

9

1

186

149

148

16

10

368

36

47

34

28

350

203

4

22

27

357

6

7

190

54

24

1267

112

4

8

258

10

1662

23

87

1

706

68

108

44

184

89

2

264

62

25

1127

21

1263

3

318

7

67

194

16

8

155

336

10

524

5

234

20

4

21

1

11

387

60

4

151

33

64

345

1

20

3

1

8

3029

31

87

201

30

7

23

2236

1

109

411

7

4

9

11

279

9

293

60

11

581

309

41

3

387

221

1

88

28

82

239

29

2505

2

183

522

32

119

200

287

8

1

332

58

9

27

36

190

618

8

43

156

72

113

1069

98

12

111

5

297

4

87

5

27

12

105

961

211

26

7

4

1

342

940

7742

1

135

207

1639

103

119

29

30

27

416

62

229

46

51

977

43

5

167

10643

50

9

64

98

2

36

59

11

2

173

4

17

14

579

15

34

14

20

4

52

16321

106

17

17

109

14

105

102

137

52

115

270

61

283

20

2

14

7711

173

2

149

125

36

120

3219

17

2

387

247

6

283

191

2175

12

8

405

61

1

14

41

143

238

92

211

6

14

4195

8

48

119

9

2590

8

11

17

627

143

35

1

1

90

1025

30

32

3

13

90

24

17

19

30

7

37

74

380

113

4

36

53

102

1

1

15

3

191

119

1

311

3

10

31

842

140

1044

16

1346

368

291

189

96

1231

3

80

6

5

1

12

179

4

544

1

25

23

71

2

77

3

13

42

5

177

8

87

26

112

59

4

63

3

14

24

88

93

290

43

1

101

215

11

1

7

56

55

697

14

1

3

463

13

830

93

6

344

13

326

324

1724
1186

538

538

36
588

552

75

45

432

1001
1391

16

374

3

8

97

181

15

1

24

1

14

2

15

4

9

392
261

131

131

297
2882

2585

8

201

7

2369

1981
472

18

1491

298

18

5

17

395

370

388

334
377

36

4

2

5

5

15

5

7

6498
6499

1

1

15

162

246
152

69

49

20

25

226
249

23

1

5

2

1

3

4

4

3

90

16

739
754

15

15

105
1

104

83

1

16

4

350
531

181

40

54

86

1

342
332

10

10

25

21

4

4

254
207

13

14

20

20

447
518

71

71

12

12

79

194
192

2

2

110

49

46

2

44

3

787
24

288
131

50

66

37

37

4

432
475

1

40

6

4

30

2

31

128
286

158

18

140

100
248

17

1

2

1

2

1

1

2

4

1

2

131

185
359

174

4

32

105

25

8

45
151

106

106

155
28

127

204
150

54

54

635
678

43

3

27

13

6792

499
1461

962

962

784

28
598

174

174

91

5

1

47

6

1

2

1

1

18

1

34

34

28

28

8

4

4

4

1

37

13

24

24

1

1

141
37

104

104

1

1

90

84

2

4

12

12

8

8

1

6

1

34

34

71
61

10

10

73

38
50

12

3

9

232
150

82

26

56

216

13141
3013

147
131

16

10

6

15

144
55

89

4

36

18

3

5

20

2

1

33
13

20

3

1

4

1

3

3

4

1

66
57

9

2

7

75

24

1613
2738

1125

25

7

6

7

19

982

48

18

13

133
177

44

44

8

361
292

69

28

4

1

3

5

1

11

8

1

4

2

1

157
196

39

6

13

1

1

10

8

92

217

1992
243

1646

92

1

1

1

1

1

1

1

2

3

6

3

3

2

1

6

1

1

1

54

1

1

11

11

173
125

48

9

39

3670

21

19

159

4

5

10

6

1

1

1

2

16

6

7

1

2

45

30

184

5

16

2

14

125

13

143

1

2

356

1

5

7

51

1

144

1

142

223

32

777

65

3

25

3

12

152

161

149

19

13

34

13

6

22

7

6

158

37

142

2

2

19

4

18

1

16

416
426

10

10

2351
58

2293

1543

750

205

4

799
999

200

4

181

15

3
124

121

24

97

97
80

17

5

1

11

1958

7

406

17

1378

150

250
257

7

7

373
131

242

11

3

76

97

55

257
496

239

24

103

1

111

183
374

191

177

14

88

2312
1635

677

505

172

190
209

19

19

218
197

8

8

5

3

5

226

33

33

6

27

155
116

4

20

15

779
327

56

19

25

7

5

14
11

3

2

1

105
136

31

1

11

3

3

6

4

1

2

138
132

6

1

2

2

1

108
74

34

32

2

146
213

67

67

21
15

1

5

2

1

2

14

14

14

750
573

177

2

164

11

83

66194
2177

94
80

14

1

3

3

7

151
110

41

10

4

2

1

3

6

15

76

76

297

96

8

345
311

34

6

28

585
263

322

92

64

46

57

2

35

6

20

196
174

22

12

10

90
17

73

73

95

13
3

10

10

108
4

88

2

38

2

16

3

2

2

3

11

1

3

1

4

2

14

5421

1

16

3

596

456

2

14

301

23

898

196

4

68

1997

628

3

37

178

3
14

7

4

50908

1

1

118

49

14

995

44

63

106

41

2

3

6

8

7

104

11853
3780

108

205

221

159

187

141

744

482

1

194

266

859
1918

307

170

52

42

155

10

249

74

273

1468

28

8

8

9

1

11

110

140

81

56

706

399

154

3

142

2

1

14

33

92

237

29

6

9

109

24

1

159

1

97

14

24

8

4

4

9

2080

152

3

166

5

1

8

3

548

11

8

168

1533

17

11

3

93

2

7

204

1

41

278

435

1

1

20

163

2

2

197

5

37

1

2

177

47

7

275

78

21

10

6

12

30

1937

3

71

63

21

24

8

6

9

10

189

11

98

14

34

182

101

89

1

6

8

13

17

70

19

3

55

479

37

148

15

727

2

15

667

1106

2

1

2

1

139

25

2

3

34

157

66

13

104

10

9

14

35

14

9

1461

1

16

23

76

1

96

1170

6

12

96

19

169

577

87

16

19

75

16

47

2

3

88

130

86

2

97

1

27

1

1511

2886

1

168

16

216

112

2

68

10

148

57

119

1

1

1978

1

51

7

187

91

102

13

226

8

1

19

6

12

3

187

7

14

1

21

1534

1

108

73

305

136

127

3566

1

131

33

606

5

28

26

4

193

14

1

3

33

75

73

28

108

4

45

332

3

10

80

3

26

238

12

139

12

165

607

432

24

102

120

438

29

306
179

25

102

16

1

2

7

13

6

4

1

44

8

215
9

161
16

6

106

33

14

19

12

2

2

24

7

17
36

19

2

3

2

4

4

4

1331

79

19

82
70

2

10

2

1

5

2

827
871

44

1

43

37
177

5

5

5

129
135

6

2

2

2

130

8

8

202
208

6

6

108
51

57

2

5

9

38

3

202
91

104

104

7

154
152

2

2

194
411

49

49

60

59

70

70

63

7

19
28

8

8

1

1

70
98

28

19

9

2

671
382

289

187

51

51

8

184

20

4

16

164

122

122

53

35

34

5040
526

107

107
49

2

56

4

2

1

8

1

2

9

3

4

5

7

5

5

176
217

25

16

6

10

59
696

23
10

6

3

2

1

7

531

531

24
83

59

13

41

5

3477

9

9

2

24

70

38

15

109

24

461

24

34

15

502

239

1902

17

1252
274

126
29

3
22

19

19

46

14

28

4

6
29

11

3

4

4

12

199
149

38

1

1

2

1

5

9

2

2

1

7

7

12

371
49

73
24

33

25

2

6

1

15

173
138

35

5

9

2

2

2

15

19
76

57

13

5

8

8

2

19

1

1

6
88

15
21

6

6

52
56

4

1

1

1

1

5

20
49

29
19

2

8

99

22

48

9

20

17
46

29

29

200133
34916

1012
1295

3

148

5

2

15

1

11

1

1

9

1

1

26

21

5

1

4

44

33

99

86
117

31

5

9

1

8

8

112
175

63

32

31

276
243

1

1

25

6

19

2

4

800
324

476

57

150

138

131

1
117

64
63

1

1

47
52

5

5

198
80

118

2

4

70

42

511
107

81
102

21

3

1

2

2

13

302
200

102

25

2

68

7

70

73
37

36

3

20

4

2

2

4

1

134
97

37

29

8

70
342

272

65

92

10

87

18

202
114

88

79

9

155
51

104

19

6

4

18

26

5

26

40
76

12

12

23

1

23

132

59
26

32

1

5

2

7

4

1

6

4

2

1

264
159

105

1

18

23

10

23

30

103

5
41

36

11

25

51

51

68
64

4

4

146
132

14

1

9

3

1

45
126

1

72

1

1

1

4

11

1

3

5

7

1

1

8

1

3

1

1

3

6

3

1

1

2

1

4

1

2

5

1

94
207

113

15

12

6

80

3
74

71

2

42

27

27
7

20

5

9

1

1

4

81

66
81

4

1

3

8

3

69
76

7

7

609
277

332

117

140

2

4

69

20

7

8

5

52

77
200

31

35

57

56
234

92

68

24

44
23

21

4

1

3

13

41
42

1

1

131
115

6

6

7

3

187
213

26

26

4

22

3

1

18

3

5

1

3

1

2

3

346

55
87

32

3

1

7

2

19

375
210

165

40

47

53

11

14

8
135

52

52

51

24

128
249

121

121

79

5

1

2

2

1

1

205

133

153
133

20

2

1

3

3

1

4

1

1

4

28

2
73

71

3

3

60

5

124
65

56
59

3

3

353

353

14
15

1

292
255

17

20

17

3

173

26
13

8

5

5

132
251

119

70

1

48

2

417
394

23

5

17

1

107
331

224
170

54

2989
263

1368

435
467

2

3

27

13

1

3

5

5

891

129
187

58

27

31

177

35
60

25

8

9

1

7

636
585

51

51

890
539

351

30

1

1

51

31

118

13

78

28

659

659

69

679

74
70

4

4

429

429

11
31

20

18

2

456

456

456

44
56

12

12

23

48

92

92

158
106

52

19

5

6

12

2

2

2

4

89
93

4

4

61

65

2
3

1

1

238
178

60

10

10

40

220
221

1

1

5145
3311

7

4

9

1

3

1

1

3

4

1

1785

13

2

3

14

1

14

7

4

12

8

1

3

8

2

1

1

2

6

6

15

1

9

1

3

6

12

5

3

2

3

12

1

19

29

7

72

1

1

8

98

27

2

10

4

3

6

7

2

1

9

4

12

1

2

1

2

6

3

2

3

1

6

11

33

5

1

16

8

8

1

1

1

29

3

39

3

11

37

3

17

6

3

30

5

6

12

1

2

25

4

3

3

1

2

2

3

1

1

3

1

7

66

1

1

11

6

95

3

3

9

18

13

2

2

13

8

18

2

5

4

12

64

30

7

1

19

10

5

46

1

3

2

4

1

3

22

31

69

9

5

24

15

1

2

3

13

64

3

1

22

3

1

1

1

1

2

7

2

5

28

27

56

1

1

4

2

7

1

12

12

71
24

3

42

1

9

32

2

252

252

316
912

6

8

582

275

12

295

205
74

131

60

71

206
400

194

194

33

30

3

148
2368

77
46

31

1

5

7

2

2

5

1

4

4

381
506

2

34

1

4

13

16

89

210
251

41

1

7

1

1

31

188
120

4

64

62

2

1016

167

25

82

132

163

20

156

100

171

172
182

1

2

7

1

6

197
141

56

29

27

52
27

25

25

55
16

39

36

2

1

7

127
188

61

2

5

2

21

7

12

6

3

3

1

1

107
211

104

1

38

63

2

2795
1511

40

30

1214

4

68

2

10

1

54

23

8

19

25

1

6

1

94

48

7

36

1

39

6

28

1

19

2

24

46

18

6

3

1

1

2

310

41

20

18

1

11

49

4

102

23

1

1

1

9

6

13

308
154

54
49

5

1

3

1

80
55

25

1

24

20

596
159

430

15

38

4

27

35

1

19

20

13

22

8

15

3

2

7

10

2

3

2

2

66

5

30

32

1

48

7

108

108

276
70

206

19

2

4

13

9

3

20

19

12

27

48

30

140
389

249

1

4

7

3

5

8

14

1

14

122

15

6

49

101127

70

37

6

8

40

46

297

85

1

51

18

1

172

22

16

35

224

367

1

157

1

2

1

9

2

3

1

39

38

3

10

12

1

1

4

169

14

23

64

56

27

26

5

318

8

9

9

4

284

2

8

1938

11

9

2

4

12

46

7

30

7

9

47

1

4

10

2

115

96

3

45

21

88

56

49

73

1

7

4

5

104

24

239

6

1

4

62

17

133

8

49

33

39

206

8

29

75

9

29

3

6

74

1

13

2

11

11

13

1

32

20

151

3705

4

129

7

2

121

6

607

7

5

1

68

28

2

8

23

29

115

13

1

13

15

1

2

175

2

13

1

259

81

1

33

12

37

14

7

433

5

38

2

6

8

3

1

3

55

101

13

37

2

84

16

3

158

97

135

37

7

3

19

2

12

20

16

100

7

12

26

20

4

2

505

27

30

8

2

7

6

6

26

7

23

394

1

3

7

25

10

122

8

3

3

5

11

8

11

20

4

13

4

18

1

41

12

690

86

1

42

50

51

9

122

20

46

75

11

8

16

1

127

89

1

11

445

222

3

7

7

3

22

3

3

184

5

248

35

240

24

76

1473

2

10

248

1

160

4

19

21

185

1

9

3

42

107

29

3

55

44

301

245

143

1

144

34

1

3

1

20

5

1

6

304

1

41

2

119

144

1

155

23

15

23

72

6

20

64

106

1

71

35

14

101

83

92

37

3

195

93

60

2

980

149

17

8

373

6

23

1

8

5196

1

29

28

150

21

1

14

183

138

9

11

36

21

65

197

5

9

2

31

5

16

20

26

210

59

450

424

11

57

3

54

72

1

5

4

83

330

65

6

98

30

5

19

3

3

8

48

10

2

89

82

2

5

560

11

1

46

30

37

8

125

393

5

95

5

133

10

29

52

2

104

144

116

44

118

292

6

2

12

25

50

126

37

21

22

16

2

1

4

85

5

6

25

14

8

144

192

22

2057

23

50

18

3

91

12

47

2

23

1569

6

278

49

7

3

11

1

29

155

2

47

192

1

1

1

4

68

16

44

30

17

5

85

55

2

23

29

1

27

12

2

3

20

166

6

60

429

2

141

1

61

17

5

103

1

3

23

14

163

45

201

1

17

5982

1

109

64

4

24

15

16

37

2

32

29

5

58

29

224

1

23

2

28

185

246

1

207

17

120

73

129

5

31

201

12

7

2

227

5

80

4

239

94

18

100

27

43

9

9

10

1

1

98

239

59

20

43

3

3

5

4

3

5

14

3

28

95

56

187

1

92

1

28

26

29

164

9

1

87

21

103

104

147

30

17

1237

38

7

45

21

9

243

6

32

170

49

66

13

245

147

2

13

4

59

162

27

1

303

4

4

162

31

3

16

6

79

8

9

28

1

75

3

1

38

56

43

16

6

144

10

36

196

55

1

59

107

32

8

171

40

2

48

68

1

50

1

60

23

16

114

212

244

7

1

7

3

231

2

7

164

15

38

273

1

2

8

410

2

3

16

77

23

37

97

9

6

17

22

1

62

23

106

1772

2

22

11

1

1

2

4

14

5

6

8

40

353

67

9

5

102

155

498

5

495

2

1

1

6

4

12

278

196

2

154

784

88

36

80

1

1

9

134

166

16

2

41

80

2

13

1

72

1

1

9

2184

498

107

2

107

239

54

2

18

8

13

44

1

18

7

68

33

32

1

3

3

1

4

5

11

1

519

46

1

61

105

2

6

101

1416

1

95

8

55

2

262

13

23

7

8

3

1

2

3

10

1

5006

21

347

1

18

47

1

112

1

27

1

26

177

1

4

2

14

245

61

75

135

51

95

3

49

3

517

12

25

2

3

719

1

2

7

1

1

36

6

35

1

51

449

1

12

3

6

5

21

1

291

1143

1

69

459

11

1

6

28

26

5

51

1

96

80

33

287

89

2

6

2

31

16

268

14

87

16

20

62

2

62

220

7

154

16

89

74

1

239

20

4

21

4

28

520

19

269

255

17

89

44

22

11

213

2

13

2

27

62

17

6

135

52

121

1

26

8

516

12

31

120

2082

1

81

175

17

73

4

55

70

30

132

2

35

16

2

35

14

28

8

34

86

4

157

37

17

68

16

17

63

87

4

109

35

51

11

46

1

2

195

22

10

3

53

29

1

23

42

1

75

224

4

78

17

35

23

111

26

9

240

28

26

16

7

5

1

52

2

1

10

405

1

3

195

2

3

6

25

1

74

4

10

2

13

27

3

21

6

47

162

11

3

1243

15

15

67

1

15

3

1

336

4

11

1

1

56

14

358

1

2

13

11

7

31

124

2

13

9

41

9

1

9

3

11

49

1

31

101

134

27

1

3

19

76

39

9

471

1

28

1

1

14

58

2

118

24

22

139

13

14

15

58

56

6

1

73

23

1

365

1

259

20

4

21

30

3

50

1

83

197

91

136

25

6

74

5

3

42

162

1

50

144

81

1

32

1

1

1

4

10

126

183

12

1

7

17

24

22

8

21

27

30

39

15

51

2

14

26

147

1

139

1

143

11

85

74

51

17
73

54

9

29

14

1

1

1

1

125
44

80

36

8

5

6

6

18

1

1

98
130

32

32

15
21

6

6

47
90

43

21

22

11

11

11

173
359

186

72

114

145
123

22

22

333
359

23

2

2

8

2

2

5

2

3

282
125

157

157

218
308

90

77

13

177
85

92

1

91

13
236

1

220

9

2

53

21

1

48

12

1

70

3

2

67

59

8

8

168
62

106

2

12

15

33

44

135
98

37

22

1

1

13

577
154

37
139

102

6

37

14

45

29
84

55

5

1

1

4

17

5

1

1

3

1

1

9

1

2

2

1

105
200

95

22

3

2

4

11

2

4

19

7

1

15

5

281
288

7

7

115
78

37

190
211

21

21

132
831

699

9

40

25

456

125

44

266
117

149

149

34

123
121

2

2

249
258

9

3

1

5

38
36

2

2

21

307
26

115
241

126

1

1

10

11

3

100

40

40

40

144
143

1

1

1196
2112

14

1

22

4

25

803

2

1

6

3

11

48

68

17

4

1

76

11

1

5

1

4

5

2

9

8

51

5

5

44

1

6

39

1

59

12

14

5

1

4

1

3

19

5

143

3

20

3

9

12

2

14

5

19

1

1

2

8

2

1

30

3

2

3

5

4

21

4
26

1

8

12

3

4

3

1

1

1

370
240

130

13

117

4

4

4

201
169

32

4

1

1

4

1

4

1

7

2

1

6

87
93

6

2

4

110
111

1

1

332
323

9

9

52
81

29

7

22

5
164

2

10

6

2

2

105

91

4

3

3

4

31

1

15
62

47

47

68
39

29

2

7

17

1

1

1

488
749

261

93

117

51

69

262
261

1

1

37
438

401

209

7

14

97

74

75
155

1

73

1

1

5

1

5

1

4

4

1

8

4

1

1

1

1

2

1

1

3

2

2

5

2

3

1

1

1

2

8

1

5

1354
438

328
68

260

46

16

2

2

61

4

15

31

2

35

19

7

11

4

5

313
249

64

6

1

25

6

4

1

8

4

7

2

104
275

171

38

36

13

38

21

25

196
21

175

6

2

33

123

11

5
26

21

21

74
31

43

11

30

2

13
128

2

2

111

5

63

5

1

6

31

9
55

46

5

2

1

38

270
387

117

117

2

2

165
492

307

3

139

97

68

20

228
229

1

1

402
369

33

14

5

2

7

3

2

250
104

146

47

5

7

1

86

47

47

6

4

1

36

19
457

5

433

1

13

1

23

386

4

2

1

1

1

142
189

47

6

41

186
354

168

87

81

98
1

97

97

23
168

55

43

42
47

5

5

310
173

137

14

106

5

12

121
125

4

4

99
63

36

36

110
145

35

4

31

219
330

111

7

8

11

4

4

5

7

6

7

1

8

4

2

10

1

5

7

4

2

8

40
41

1

1

25
113

88

68

20

260

180
68

112

28

12

10

24

38

412
323

89

7

18

1

2

5

2

1

3

3

10

2

1

19

12

2

1

43
12

31

28

3

16

16

4

12

11480

76

44

148

1818

135

547

164

376

106

1202

92

277

9

120

366

211

139

211

627

100

57

3

63

159

1090

73

73

53

205

2936

2
29

27

1

6

15

4

1

62
396

56
24

32

32

47
65

18

15

3

53
9

44

44

37

98

65

33

25

8261
570

55

149

149
147

2

93

981
3469

153
128

25

343
197

146

39

1

26

26

54

400
521

121

1

20

100

144
170

26

1

4

3

2

6

8

2

671

109

70

14

309

169

178
168

10

2

5

3

372
281

91

13

2

4

53

19

80
76

4

1

3

715
384

153
79

74

6

4

64

131

8

123

22
47

25

17

7

1

1640
100

284

821

300

302

219

435
319

8

108

100

8

1151
170

163
160

3

3

174
257

83

14

10

2

9

14

14

1

1

2

2

5

1

8

112
102

10

10

60

60

23
51

28

3

20

5

79
154

75

75

111
184

71

9

5

56

1

2

419

121

49

63

33

126

27

33827

33827

476
33827

204
239

8

8

25

25

2

2

7
4363

21
33

2

2

8

2

22

5

15

2

2951
553

578

1

1

537

623

659

739

360

379

611
210

43

254

71

71

33

309

1

1

299

1

7

28440
512

1262
14002

280

7

1

4

1

14

2

137

22

1

80

1

10

6648
703

29

278

213

325

123

2580

247

316

61

50

50

43

1680

1568
121

1

1

216

216

114

233

239

428

3368
612

67

217

110

41

491

12

9

203

241

180

156

81

31

7

798

13

99

10
645

253

382

231
3

31

197

309
5855

1677
348

186

58

64

50

398

105

230

186

5

1

2

2

1

1

5

5

6

8

2

5

1

238

1119

1

1

1

264

15

4

203

2

1

2

2

3

1

146

2

1

2

5

324

3

2

1

2

126

1

4

729
172

201

94

144

2

116

61
508

255

2

190

338
1273

151

16
60

4

1

6

4

1

24

4

9
3

1

5

5

75

2

174

36

475

99

240

7896
111

799
3477

262

168

435

264

74

2

11

1

12

6

2

1

1

1

2

1

7

14

1

6

3

3

453

1022

391

384

5

2

869
108

537

168

6

34

26

302

1

224

300

561

1091
73

201

772

19

26

26

1096
294

91

127

9

146

429

130

3

3

293

175

13

3

2

8

10

2

3

122

3

1

1

6

1

34
29589

21099
7

26
12191

8461
2

1297

417
1297

38
127

9

2

2

3

12

8

4

8

3

7

4

5

1

2

11

2

4

1

1

24
65

11

1

12

7

10

10

418

4

1

3

6

7

2

2

5

20

5

4

5

9

5

29

12

3

8

1

1

2

6

4

4

9

12

4

8

14

5

4

11

5

15

7

1

8

7

32

3

4

4

12

5

3

9

13

18

6

3

4

14

17

3

2

4

4

3

12

5
51

10

22

5

3

6

6

3

178
36

10

10

13

4

7

10

24

3

7

6

19

8

7

2

6

1

2

2

3

13

7162

7162
2520

3271
144

16
300

9

9

6

2

4

6

6

29

8

2

19

3
7

4

7
1

6

1
15

1

9

4

4

4

8

4

4

13

11

2

1

1

7

7

3

3

34
17

2

2

2

8

3

1
7

4

2

11
15

2

2

1

1

20
3

2

1

1

3

10

19
4

2

13

1
5

1

3

7
2

3

2

1

1

30
1

5

1

4

12

1

1

5

1

1

29
13

3

11

2

1
36

35

133
11

11

11

21

4

17

9

9

1

25

6

21

28

24

24

24

22

22

13

9

23

1
23

14

8

2
26

11

11

13
4

7

2

52

52

12

40

25

25

25

35

28

13

15

7

4

3

12

12

12

7

7

7

20

20
4

9

7

27

27

27

3

3

3

15

15

15

5

5

13

13

13

5

5

5

32
1

10

10

21

21

18

1

1

5

5

12

12

1
21

9

11

11

20

2
20

1

17

10

10

10

68

30
2

17

11

38

16

22

7
236

12
98

16

16

29

25

20
106

13

15

9

6

9

3

31

25
12

13

19

19

45

2
30

21

7

15

3

7

5

24

24

24

88

24
88

18

16

30

113
2

19

19

18

1

17

21
3

9

9

48

15

5

28

5

5

38

9

9

15

7

8

1
14

13

23

1
23

5

6

3

8

5
26

12

9

4

5

145
7

9

9

3

3

54
22

1

4

1

7

1

1

6

1

3

1

6

3

1

2

2
4

2

13
8

2

3

12
52

7

3

1

2

1

2

1

1

1

5

1

9

5

1

866
62

20
9

1

5

5

27
5

7

3

12

80
29

1

1

7

5

10

3

9

4

11

14

14

2
39

4

1

4

7

4

10

7

69
47

2

1

13

6

20
53

25

3

2

3

15

6

9

29

29

4
30

8

4

14

90
5

4

33

9

5

20

6

8

137
52

6

14

4

4

10

7

1

17

2

3

17

43
130

9

8

12

4

3

15

6

11

2

11

6

34
5

10

13

6

6
37

12

6

13

21

21

21

20

1
20

2

4

13

7

7

7

26

26

26

5

5

5

13

13

13

7
65

12

12

16

16

6

6

24

24

10

10

10

71
4

23

15

8

1
6

5

2

9

9

11
27

14

2

18

18

18

26

26
8

11

7

91
7

6
36

2

7

2

13

6

25

11

5

5

4

7

7

4

4

3

3

9

9

41

9

9

22

22

10

10

14

14

14

20

20

20

104

34

34

8
43

12

11

12

27

27

1362

49
2

11

11

7

5

4

4

2

3

353
20

5

9

2

2

5

1

11

5

1

15

16

17

2

19

44

2

3

15

1

8

2

2

17

2

26

6

3

2

2

10

13

6

3

44

1

4

2

6

1

28

25
33

4

3

1

35
899

2

823

6

6

8

15

2

2

9

9

9

9

26

26

26

13

2

11

13

13

13

3678

3678

391
3678

202

202
2

200

2

5

88

46

6

7

2

39

5

85
770

68
3

7

58

44

6

8

2

2

13
16

1

1

2

30
10

20

19

1

320

6

14

14

2

14

8

2

6

10

42

4

7

2

1

1

17

3

5

2

19

12

11

50

5

2

3

2

7

1

32

10

4

2

249

249

1490
246

27
3

3

21

6

8

7

30

1

29

11

10

8

27
3

11

13

13

51
214

5

9

10

3

136

5

28

3

1

1

2

9

32

2

26

9

5

4

2

1

4

2

42

236

1

2

145
3

7

9

6

114

9

19

2

14

10

7

32

11

3

7

6

9

1

1

1

6

35

4

10

3

16

2

32
36

4

2

2

144
63

43

15

12

3

4

2

2

1

4

4

5

4

8

9

8

488
301

8

1

158

6

1

5

2

2

6

11

4

10

11

6

12

2

30

7

1

2

8

3

6

14

1

2

4

2

5

6

9

825

1

658

2

8

1

6

7

1

3

5

4

11

4

4

3

5

6

1

2

12

8

22

13

1

1

3

22

8

3

8901
300

2899
7

112

5
112

9

3

6

37
11

19

4

3

15
58

7

1

2

3

2

6

1

2

3

15

5

2

3

221

12
221

131
25

2

1

2

5

3

3

87

2

1

3

1

4

23

7

6

1

6

6

5

14

3

1

4

2

1

7
6

1

1

59

6

4

3

2

25

18

1

12

2

10

90

90
10

1

1

21

21

21

21

21

33
1

8

1

9

14

4

4

1

1

1

1

2468

132
2468

87
40

4

19

1

4

2

1

1

5

1

1

3

1

1

3

3

5

1

1

2

1

1

3

2

1603

2

1

1

3

5

2

3

15

1

30

2

1

44

1

4

5

2

2

2

4

2

20

16

3

6

30

2

1

1

4

5

2

1

8

1

6

2

8

2

2

1

1

5

1

6

3

1

4

5

5

9

1

6

1

4

2

1

8

6

1

5

4

3

1

1212

1

5

1

6

4

2

3

2

2

2

12

1

8

6

70

3

6

1

4

4

4

4

3

2

2

5

7

1

2

10

2

10

49
576

1

5

42

63

10

261

3

2

2

2

4

3

3

2

6

1

5

95

2

11

6

5

6

18

2

5

3

2

5

2

13

7

7

39

2

14

1

1

1

4

41

4

1

1

1

2

3

4

14

2

41

7

1

1

3446

10
3446

147
2746

13

6

7

7

191

7

5

1

5

44

7

17

5

8

9

4

3

7

4

2

4

3

5

1

34

4

2

10

20
1

1

3

12

3

20
5

12

3

8
1

7

2

2

1019
2030

1

3

1

4

2

37

28

3

1

2

1

3

1

3

2

1

2

11

1

2

1

4

4

2

6

10

3

10

3

1

3

4

1

2

1

3

4

1

1

6

9

3

3

4

2

3

1

1

3

1

6

2

15

1

2

2

1

3

5

7

5

4

1

13

4

2

7

4

2

3

8

10

7

3

1

1

3

7

1

84

2

2

1

2

6

1

3

5

4

9

1

8

1

3

1

1

1

3

1

1

3

1

8

4

5

4

2

1

1

3

14

3

1

1

1

3

3

2

2

1

3

18

2

1

3

2

4

4

3

8

5

3

1

1

2

2

7

3

1

9

7

9

5

1

26

2

13

10

17

5

2

1

1

19

5

8

4

7

1

1

1

9

2

8

1

3

3

3

5

14

1

3

1

3

1

1

3

5

1

1

1

1

18

4

3

2

3

1

14

7

1

2

16

7

1

5

1

8

1

3

63

1

18

3

3

2

2

30

4

5

2

3

2

1

1

2

8

1

8

9
14

3

2

16

261
49

4

1

7

8

2

2

2

2

3

3

3

47

1

4

1

4

19

3

5

2

3

1

2

1

1

6

2

2

6

7

10

4

1

10

5

5

8

11

9

17

7
2

5

690
248

158
28

98

1

7

62

1

2

2

1

1

3

1

4

1

9

2

1

2

1

1

1

7

2

1

5

12

7
3

2

2

2
7

5

5

244

3

4

8

2

3

1

2

2

2

3

1

1

1

2

4

1

2

3

1

1

1

2

1

1

8

1

3

3

1

1

11

1

2

4

6

3

6

2

2

2

4

1

1

5

3

1

7

14

1

2

1

12

27

4

2

3

10

1

9

1

2

3

6

2

1

1

1

1

4

1

1

1

3

26
1

4

2

2

1

2

14

3

10

1

2256

2

4

12

6

16

20

3

2189
7

3

6

19

5

1

3

2101

4

1

9

6

1

4

3

16

1

3

805

805

805

805

805
140

9
4

5

2

4

4

4

3

3

631

547

4

2

1

14

9

5

3

1

7

38

1

10

7

3

5

5

263

263

263

263

263

36

227
3

224

2

222

7388

7388

7388

7388

543
7388

99

17

12

70

17
3

3

4

1

3

3

7
5

2

2

316

2

2

17
13

4

297

4

9

33

6

2

183

50

3

4

1

2

6413

10

1

5

13

4

4

11

13

5

25

9

3

6

10

5

10

2

16

1

3

4

3

6

3

4

20

2

18

5

4

5

3

2

491
1

417

73

6

1

6

1

3

1

10

4

7

4

1

7

4

6

3

1

2

2

4

4

12

5

3

11

2

4

1

3

1

3

128

1

2

19

3

2

6

6

1

6

1

4

2

12

7

28

13

13

2

2

2

3

13

1

3

4

3

1

16

13

2

2

18

5

6

1

4

1

20

2

2

11

5

5

5

9

1

12

5

2

10

1

4357

4

8

2

4

3

2

1

1

8

16

3

4

2

13

2

5

1

13

1

1

12

2

4

3

2

1

3

4

3

9

1

2

14

2

1

8

9

3

16

6

2

8

5

3

8

4

9

1

20

6

2

3

1

5

4

3

9

9

9

2

5

3

12

4

9

1

5

8

4

2

6

1

7

3

17

13

1

4

7

3

1

16

9

6

4

6

4

3

2

13

20

5

3

4

5

3

1

2

4

1

5

1

10

2

2

19

6

3

4

2

6

3

4

1

11

4

1

5

1

3

1

5

1

6

2

1

3

4

10

4

5

3

3

3

6

1

8

8

5

2

2

2

1

4

2

8

3

2

3

14

1

3

4

2

6

5

7

2

5

1

4

6

2

7

3

3

7

9

4

12

12

223

223

223

16

16

16

19900
30

108
1931

856

130

216

189

213

108

338
967

108

108

521

62
17939

16263
1842

36
146

2

108

108

977
3608

182

145

116

123

121

88

97

170

1

124

97

38

151

155

2

35

167

105

133

229

75

135

4

1

13

1

154

176

22

28

1

7

1

3

2

11

1

2

1542
957

311

311

274

9097
3827

57

2

85

36

45

119

114

23

63

171

103

8

86

24

118

182

50

153

150

98

2

41

4

87

3

4

13

98

22

25

141

109

60

391

105

105

178

309

142

66

127

103

43

103

159

2

37

51

69

926

22

115

8

10

22

96

3

1

3

23

1

138

1

9

42

1

129

9

89

1

70

44

2

20

61

6

58

1612
20

324

224

100

979

137

71

78

84

1

100

86

66

60

2

89

205

289

289

2

515

515

515

515

479

86
283

93
197

6

10

15

13

43

17

196

66
196

11

101

6

7

6

23

7

7

1

1

14

28

1

11

7

36

24
36

4

4

8

8

3825
639

4

4

12

12

2058

15

564

3

22

37

7

5

13

6

42

1

21

22

1

17

5

3

19

3

2

4

6

37

2

21

5

17

5

163

27

10

5

758

11

3

123

5

23

12

11

2

13

4

9

165
13

108

27

17

17

56

6

3

9

3

8

6

4

2

10

3

2

5
437

2

3

10

3

2

14

14

2

30

14

289

1

6

6

2

12

214

3

5

11

14

4

11

1

1

6

1

37

3

4
180

1

105

84

5

5

11

9

14

1

5

4

27

3

4

3

4
168

134

14

19

5

3

7

14

1

17

1

15

2

14

2

20

30

30

22

8

2

5

1

13
63

37

13

144883

62978

260

10

4

63

32

14

6

3

7

1

21

24213

57271

104

84

2

1

1

1

4

3

2

12

6

54

4

4

31

4

27

2
19

2

15

20

7

3

1

3

1

12

9

6

3

392
2

21

21

369

369

1
500

390

10

223

99

28

12

8

10

109
88

1

20

20

31

31

3
149

20

11

2

1

6

116
35

46

1

1

5

2

8

6

11

4

1

2

5

2

3

3

7

6

1

23
1

9

13

10
1

3

3

2

2

1

1

1

1

2

2

357686

2

7

1653

2

30

5

7

1

5

1

7

2

15

29

12

1

1

3

4

1

1

4

5

36

5

1

5

9

5

9

11

2

11

13

6

8

1

4

8

22

1

22

57

5

4

18

5

2

1

1

16

11

108

11

2

43

7

10

4

4

9

9

3720

1

566

8

2

3

1

12

5

8

2

3

11

3

4

4

3

2

834

5

12

3

3

2189

9

1

12

2

3

15

9

3

2

7

76

6

1

6

5

7

18

10

7

2

3

1

9

4

13

1

3

29

2

3

4

6

1

2

6

3

5

5

10

1

42

1

12

185

5

3

2

8

2

1

3

17

1

5

8

3

1

4

6

4

601

1908

21

27

1

228

5

1

10

6

2

2

5

1

1

9

4

6

5

1

8

3

6

2

3

4

65

5

4

6

4

1

2

760

6

2

14

3

11

4

3

5

25

3

38

4

11

3

11

1

2

5

1

4

1

33

5

3

2

3

8

6

13

8

11

4

23

4

13

3

3

20

431

1

4

12

3

14

2

3

2

28

6

1

47

7

2

10

3

43

2

321263

1

48

1

2

5

270

11

2

2

150

11

2

3

1

10

2

41

41

5

710

151

369

21

136

1

12

20

18

2

2

1

23

3

327

3165

142

19

82

299
2885

41

233

233

121

162

751

8

2

2

85

9

3

10

33

149

11

39

3

68

7

287

5

11

19

99

192

24

55

170

738

37

3

1

1750

6

7

1

5

4

6

3

1

2

13

1

1

1

9

5

2

3

14

3

2

5

2

2

9

4

2

10

44

2

4

6

5

1

4

2

5

2

1

4

3

2

2

6

23

2

6

1

7

8

4

5

3

4

1

2

8

1

3

2

8

3

1

1

13

1

8

2

4

3

1114
3

1014

4

1010

2

3

8

5

2

1

9

1

2

4

8

4

11

3

5

4

1

12

2

3

8

4

3

7

4

3

31

2

4

2

3

5

8

1

6

2

15

16

13

19

12

3

5

7

4

1

6

1

11

3

1

3

1

2

2

1

590

1

2

1

1

10

15

16

5

31

4

2

1

3

15

4

4

92

1

1

1

3

9

2

7

10

5

3

1

2

5

3

11

9

4

1

14

6

5

23

4

9

1

4

1

2

2

4

2

4

2

2

5

2

1

2

3

5

4

3

1

3

1

6

3

2

13

2

4

2

1

1

10

1

2

2

8

3

1

2

2

25

1

3

20

3

1

3

7

131

11531

2

302502

12

7579
36726

7226

658

868

83

1214

558

2422

1637

1637

720

2951

67

388

872

788

667

1001

561

84

2292

564

1146

1913

467

456

5849

36

5440

52041
22512

7387

4357

639

7757

4271

1299

3819

1010
290

222

41

39

56

323

39

77
74

3

4

200300

3

500
7

60

5

50

18

12

66

12

66

14

13

34

5

29

41

23

36

36

2

7

27

21

6

47

2

4

3

3

1

2

3

41

35

65

70

230

3

1

6

3

3

60

4

10

5

1

26

3

1

7

23

2

10

8

8

3

130

12

1

7

4

16

1

5

11

16

3

27

3

4

5

22
61

1

4

2

7

16

9

16

1

13

1

4

13

19636

15

7

19

3

2

7

2

9

12

1

6

1

1

2

2

2

5

2

4

6

4

11

48

8

1

11

3

12

1

2

5

18

2

2

7

2

5

11

2

6

4

810

1

2

45

7

8

11

30

4

2

5

1

8

11

1

17

12

11
